# Hypoxia and inactivity related physiological changes precede or take place in absence of significant rearrangements in bacterial community structure: The PlanHab randomized trial pilot study

**DOI:** 10.1371/journal.pone.0188556

**Published:** 2017-12-06

**Authors:** Robert Šket, Nicole Treichel, Susanne Kublik, Tadej Debevec, Ola Eiken, Igor Mekjavić, Michael Schloter, Marius Vital, Jenna Chandler, James M. Tiedje, Boštjan Murovec, Zala Prevoršek, Matevž Likar, Blaž Stres

**Affiliations:** 1 Department of Animal Science, Biotechnical Faculty, University of Ljubljana, Ljubljana, Slovenia; 2 Research Unit for Comparative Microbiome Analysis, Helmholtz Zentrum München—German Research Center for Environmental Health, Neuherberg, Germany; 3 Department of Automation, Biocybernetics and Robotics, Jozef Stefan Institute, Ljubljana, Slovenia; 4 University of Ljubljana, Faculty of Sport, Ljubljana, Slovenia; 5 Department of Environmental Physiology, Swedish Aerospace Physiology Centre, Royal Institute of Technology, Stockholm, Sweden; 6 Center for Microbial Ecology, Michigan State University, East Lansing, Michigan, United States of America; 7 Laboratory for Artificial Sight and Automation, Faculty of Electrical Sciences, University of Ljubljana, Ljubljana, Slovenia; 8 Department of Biology, Biotechnical Faculty, University of Ljubljana, Ljubljana, Slovenia; 9 Center for Clinical Neurophysiology, Faculty of Medicine, University of Ljubljana, Ljubljana, Slovenia; Medical University Graz, AUSTRIA

## Abstract

We explored the assembly of intestinal microbiota in healthy male participants during the randomized crossover design of run-in (5 day) and experimental phases (21-day normoxic bed rest (NBR), hypoxic bed rest (HBR) and hypoxic ambulation (HAmb) in a strictly controlled laboratory environment, with balanced fluid and dietary intakes, controlled circadian rhythm, microbial ambiental burden and 24/7 medical surveillance. The fraction of inspired O_2_ (F_i_O_2_) and partial pressure of inspired O_2_ (P_i_O_2_) were 0.209 and 133.1 ± 0.3 mmHg for NBR and 0.141 ± 0.004 and 90.0 ± 0.4 mmHg for both hypoxic variants (HBR and HAmb; ~4000 m simulated altitude), respectively. A number of parameters linked to intestinal environment such as defecation frequency, intestinal electrical conductivity (IEC), sterol and polyphenol content and diversity, indole, aromaticity and spectral characteristics of dissolved organic matter (DOM) were measured (64 variables). The structure and diversity of bacterial microbial community was assessed using 16S rRNA amplicon sequencing. Inactivity negatively affected frequency of defecation and in combination with hypoxia increased IEC (p < 0.05). In contrast, sterol and polyphenol diversity and content, various characteristics of DOM and aromatic compounds, the structure and diversity of bacterial microbial community were not significantly affected over time. A new in-house PlanHab database was established to integrate all measured variables on host physiology, diet, experiment, immune and metabolic markers (n = 231). The observed progressive decrease in defecation frequency and concomitant increase in IEC suggested that the transition from healthy physiological state towards the developed symptoms of low magnitude obesity-related syndromes was dose dependent on the extent of time spent in inactivity and preceded or took place in absence of significant rearrangements in bacterial microbial community. Species *B*. *thetaiotamicron*, *B*. *fragilis*, *B*. *dorei* and other Bacteroides with reported relevance for dysbiotic medical conditions were significantly enriched in HBR, characterized with most severe inflammation symptoms, indicating a shift towards host mucin degradation and proinflammatory immune crosstalk.

## Introduction

The structure and function of the gut microbiome is closely linked to human health and disease. Recent studies have clearly demonstrated that modern humans in general lack a substantial portion of natural diversity in their intestinal microbiome rendering them more susceptible to dysbiosis [1,2]. The close affiliation between the triad of diet, metabolism and exercise was clearly outlined recently as increased exercise and dietary extremes shaped microbial diversity in professional athletes [3,4]. Most studies in this field have focused on comparing groups with already developed symptoms and the consequent effects of restoration of physical activity in (previously unfit) population. Reduced risk for several metabolic, inflammatory and neoplastic conditions has been observed as a consequence of exercise introduction [5–8]. For example, exercise was recently linked to modifications of the gut microbiome [3], increased vagal-nerve tone at rest [8], gene expression of transport proteins [9], as well as exercise related immunological responses [10–12]. However, the relationship between exercise, intestinal microbiota, human physiology and diet has not been explored during acute cessation of exercise in healthy test participants leading to deconditioning of various subsystems, similar to medical conditions such as fractures, chronic heart failure or obstructive pulmonary disease and obesity related syndromes.

**Fig 1:**
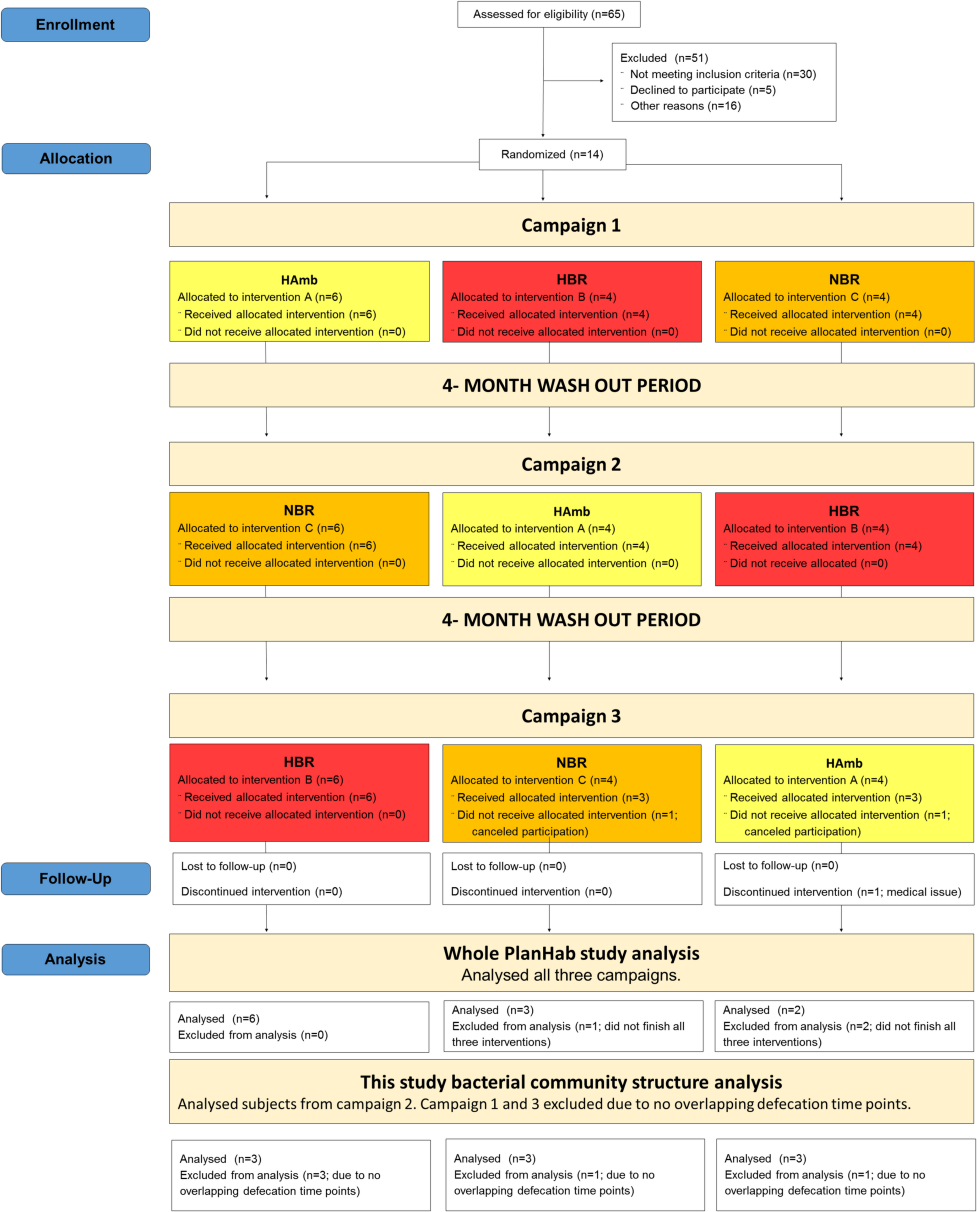
CONSORT flowchart of participants recruitment and the cross-over designed PlanHab study trial flow. Interventions: normoxic bed rest (NBR), hypoxic bed rest (HBR) and hypoxic ambulation (HAmb).

Our recent study Sket et al. [13] took advantage of the experimental setup of the PlanHab project (Fig 1, S1 Fig) [14,15]. This study was designed to investigate the combined effects of prolonged (21-day) complete inactivity and hypoxia on healthy-participants under strictly controlled conditions according to ESA/NASA core bed rest data collection SOP [14]. This approach included controlled laboratory environment with balanced daily nutrition intake between groups, controlled exercise, circadian rhythm, microbial ambiental burden, and 24/7 medical surveillance [13,14,16]. Bed rest period induced system wide negative modifications in host physiology [13–19], in addition to constipation and increased intestinal inflammation whereas a number of parameters such as gut permeability, metabolic markers, abundance, diversity and community structure of butyrate producing microbial communities remained unaffected [13].

Here, we examine the structure, composition and diversity of microbiome and additional parameters describing intestinal environment in the same PlanHab participants to elucidate the outcomes of physical inactivity in healthy male test participants during the run-in and consecutive experimental phases. We hypothesized that the cessation of exercise and hypoxia would result in quantitative and qualitative differences in microbial community structure that would enable elucidation of the first responding taxa linked to the inflammation of the host. Secondly, based on observation that significant changes in a number of intestinal parameters developed over time, we hypothesized that correlation of changes in microbial community structure and environment would enable identification of the key structuring environmental parameters. Our third hypothesis was that the timing of physiological changes coincided with concomitant changes in microbial communities over the course of 21-day experiment. Last, using the observed patterns in human physiology, intestinal tract microbiota and environment observed in this study, we searched for matching multiscale patterns reported to take place in other mammalian species within comparable timeframes in nature.

## Materials and methods

### Study design and setting

In the frame of the present substudy conducted within the PlanHab project (registration number NCT02637921 at http://cordis.europa.eu/project/rcn/104127_en.html) the dynamics and diversity of the gut microbiome was studied in response to reduced physical activity and hypoxia using stool samples as proxy. The study had received approval by the National Committee for Medical Ethics at the Ministry of Health of the Republic of Slovenia. The detailed outline of the PlanHab project is given elsewhere [13–15] (S1 and S2 Texts) and is briefly summarized below.

The PlanHab study experiments were performed between June 2012, when recruitment started and January 2014, when the last follow-up periods ended (Fig 1). This study was performed within the premises of the “Hypoxic Facility” of the Olympic Sport Center Planica in Rateče, Slovenia, located at 940 m of altitude and was conducted according to the European Space Agency's standardization plan for bed rest studies (ESA, 2009) [13–20], including sample size calculation. For this study, each participant underwent 5 days of baseline data collection during which participants were ambulant, 21 intervention days and 5–14 days of medical follow-up [14]. The participants underwent the following three protocols: (1) normobaric normoxic bed rest [NBR; fraction of inspired O_2_ (F_i_O_2_) = 0.209; partial pressure of inspired O_2_ (P_i_O_2_) = 133.1±0.3 mmHg]; (2) normobaric hypoxic ambulatory confinement [HAmb; F_i_O_2_ = 0.141±0.004; P_i_O_2_ = 90.0±0.4 mmHg; ~4,000 m simulated altitude]; and (3) normobaric hypoxic bed rest [HBR; F_i_O_2_ = 0.141±0.004; P_i_O_2_ = 90.0±0.4 mmHg; ~4,000 m simulated altitude]. Altogether 11 healthy men underwent all 3 campaigns in randomized crossover design of PlanHab project. Sample size was determined based on previous reports of bed rest studies to obtain sufficient predictive power ≥ 0.80 [14,15,19]. Subjects were enrolled by project manager and randomly allocated between campaigns using latin square design method (Fig 1) [14,19,21]. Due to the lack of overlapping defecation time-points the number of participants was decreased to 9 with a mean age (± SD) of 27.4±5.6 years, a height of 180.2±5.0 cm, a mass of 75.1±10.3 kg and a body mass index of 23.1±2.7 kg/m2 (S1 Table, Fig 1) [13]. Participants were given concise explanation regarding the experimental procedures and potential risks before giving their written informed consent. Exclusion criteria included history of any cardiorespiratory, musculoskeletal, neuro-logical or vascular disease. Except for transient headaches and backaches, all 9 participants concluded the PlanHab experiment without any significant injurious health-related issues.

### Acquisition of clinical, exercise, dietary and ambiental data

Environmental conditions were controlled (ambient temperature: 24.4±0.7°C, relative humidity: 53.5±5.4%) or assessed (ambient pressure: 91.2±5.3 kPa). The light to dark cycle was set to 16:8 h, with bed rest time between 23:00 and 7:00 for all participants.

Capillary oxyhemoglobin saturation (SpO_2_) was measured daily at 7:00 using a 3100 WristOx device (Nonin Medicals, Minnesota, USA) and also as part of a sleep polysomnographic study. To the latter purpose, full ambulatory polysomnography (Nicolet One, Viasys, Healthcare, Neurocare, Madison, WI, USA) was performed using standard setups [19].

During the bed rest phase of the NBR and HBR campaigns, the participants were confined to bed in the horizontal position for 24 h/day, and all activities of daily life took place in bed. One pillow was allowed for head support. Showers were taken on a specific gurney, and hospital equipment was used for bowel movements and urine collection. No physical activity was allowed during NBR and HBR campaigns, except for changing position between supine, prone and lateral. During HAmb, participants were confined to the hypoxic area, but remained ambulatory during day. In order to replicate their habitual bone loading during the confinement periods, participants performed low-level physical activity in two 30-minute bouts per day. Telemetric heart rate monitoring was used to achieve the targeted heart rate (123±4 beats/min) during the exercises.

During HBR and HAmb campaigns, normobaric hypoxia was generated within the hypoxic area by a vacuum pressure swing adsorption system (b-Cat, Tiel, the Netherlands). Regulation of O_2_ concentration was actuated within each room at 15-minute intervals. For safety reasons, participants carried portable O_2_ sensors (Rae PGM-1100, California, USA) at all times [14,19].

The participants were provided with an individually tailored, standardized and controlled diet throughout the intervention. Energy requirements were assessed using the Harris-Benedict method, and correction factors of 1.4 and 1.2 were used to account for activity levels in the ambulatory phases and the bed rest phases, respectively [14]. In addition to a controlled intake of fat (30%) and protein (1.2 g per kg body mass), sodium intake was set to 3500 mg per day. Participants were supplemented with 1000 IU vitamin D3 per day. Fluid intake was *ad libitum*, but participants were encouraged to drink at least 28.5 mL per kg per day. Based on dietary data of ingested amounts and types of food the collected PlanHab Carbon to Nitrogen ratio was calculated to be 16±0.47 and thus resembled a typical Central European diet.

### Sample collection

Fecal samples were collected using asseptical approaches at the time of defecation in order to prevent cross-contamination. Longitudinal sampling was performed with sampling at days -5 and -1 before the onset of experiments and days 3, 10, 18 and 21 of the intervention. Altogether 54 samples were collected and immediately frozen at -20°C. Samples were further aseptically aliqoted under frozen conditions (-20°C walk-in room) for the analyses of metabolites, immunological markers and microbiome.

### Establishment of the PlanHab database

Clinical, metabolic, inflammation, immune, human physiology and nutrition data next to experimental design and characteristics of the individual participants were integrated into a novel in-house database comprising all measured variables in the PlanHab experiment (n = 231; S2 Table) [13–17,19]. Together over 12,000 entries of examined factors were compiled and critically assessed. A comprehensive PlanHab database with entries corresponding to samples used in this study was used for data normalization, extraction and interpretation of statistical significant features. In addition to parameters recorded within the past PlanHab substudies [13–19] such as body mass and composition, menu composition, water intake, micronutrients, miRNA expression profiles, morning resting heart rate, capillary oxyhemoglobin saturation, ratings of perceived appetite, daily dietary intake, inflammation markers (n = 167), new data were measured. In this study 64 variables spanning electrical conductivity, fecal polyphenols and sterols and their diversity, as well as analyses of various dissolved organic matter (DOM) spectral characteristics (see below) were obtained and integrated within the PlanHab database.

### Intestinal electrical conductivity (IEC) as measure of fecal ionic strength

Samples (10 g) were suspended in distilled water (25 ml) and homogenized at 4°C. Measurements took place after samples equilibrated at 28°C using WTW electrode Cond 330i (WTW, Germany). External standard solution (1 M KCl) and series of its 10 fold dilutions were prepared. Aliquots of prepared standard solution and its dilution series were immersed in the same water baths and their conductivity determined in triplicates (R^2^ = 0.998). In addition, conductivity of standard solutions were measured after each five samples in order to check for electrode fluctuations. The aliquots of standard solution used for control measurements with samples, were measured again and compared to unused aliquots of standard solution. Intestinal electrical conductivity (IEC) served as a measure of ionic strength of intestinal environment that is a measure of effects of innate immune system, influencing the complex interplay between mucus thickness, porosity, its crosslinking and hydration state.

### Fecal polyphenols and sterols as determined by HPLC

Methanol (Sigma) was used to extract polyphenols and sterols from fecal dry matter in (vol. to wt) ratio 10 to 1. Resulting extracts (1 ml aliquots) were filtered through 0.22 μm filters into 2.5 ml HPLC vials (Varian) and stored at -20°C until HPLC analysis. The HPLC method was performed under isocratic conditions at 30°C on Waters HPLC system (2995 with 2998 PDA detector). Analyses were performed on a reversed-phase C-18 column: Spherisorb ODS1 RP-18, 5 m, 250 x 4.6 mm from Waters (Miliford, USA). Bile acids were separated using mobile phase composed of 0.5 M acetate buffer with 0.02% sodium azide (Sigma), adjusted to pH 4.3 with o-phosphoric acid. The flow was set to 0.5 ml/min (isocratic conditions) with the detection of PDA detector set at 205 nm. The injection volume was 10 μl. Polyphenolic compounds were separated using the two-component solvent system: A: 1.5% phosphoric acid and B: methanol (Merck)—acetonitrile (Merck)—water, 1:1:1 (V/V/V). The separation gradient of the two solvents used was the following: 0 min: 100% A; 0–20 min: 100–60% A; 20–35 min: 60–0% A at 1 ml/min and the signal of phenolic compounds was determined at 280 nm using PDA detector. The injection volume for polyphenols was set to 100 μl.

In analogy with fast fingerprinting of microbial communities, separate chemical fingerprinting of sterols and polyphenols was used to determine the total content and relative distribution of peaks within each class of compounds that were further used for calculation of chemical diversity of compounds in samples using program mothur [22].

### Deconvolution of dissolved organic matter (DOM) spectral derivatives of biological importance

Fecal samples were centrifuged at 13.000 x g and dillutions (1:10; 1:50; 1:100) of supernatants in MQ were prepared. The 200 μl aliquots of each dilution were transferred into 96 well of transparent UV resistant microtiter plates (Greiner UV-Star®, Greiner, Germany). Spectra spanning from 200 nm to 800 nm with 5 nm step were recorded using a photometer (Multiscan® Spectrum # 1500; Thermo Fisher Scientific Inc., USA). Absorbance measurements were transformed to Napierian absorption coefficient (a) using equation: a = 2.303* A/l, where l represents the length of the beam = 2/3 cm. From the absorption spectra various parameters were calculated according to Helms et al. [23] to characterize chromogenic dissolved organic matter. The following parameters were determined: (i) indole level index calculated as the ratio between two characteristic wavelengths of 217 nm and 365 nm and 287 nm and 365 nm [24]; (ii) specific ultraviolet absorbance (SUVA_245_), a measure of the aromatic character of dissolved organic matter, was calculated as a ratio between absorbance at 254 nm and total soluble organic carbon per gram of dry matter in feces (TSOC) [25]; (iii) specific visible absorbance (SViA_420_), provides a measure of nonaromatic DOM and was calculated as a ratio between absorbance at 420 nm and TSOC [26]; (iv) cDOM index, was calculated as the ratio between colored DOM (measured as the absorption coefficient at 350 nm) and TSOC [27], as an indicator of anaerobic degradation of plant polymeric substances producing tannin-like compounds stable in the water soluble fraction of DOM; (v) the sum of nine p-hydroxy, vanillyl, and syringyl lignin phenols (TDLP_9_) calculated using the model ln (TDLP_9_) = −2.282 · ln (*a* (350)) − 8.209 · ln (*a* (275)) +11.365 · ln (*a* (295)) + 2.909 [28]. Various phenols exhibit important roles in the initiation and/or progression of intestinal permeability leading to “leaky gut” and increased inflammation.

### Amplicon sequencing of bacterial 16S rRNA genes using V1-V2 and V6-V7 hypervariable regions

Total genomic DNA was extracted from fecal samples using the MOBIO Power Fecal DNA extraction Kit (MOBIO; California, USA) according to the manufacturer’s instructions using triplicate homogenized subsamples of 0.25 g fresh weight. Concentration and purity (A_230_, A_260_, A_280_) were determined spectrophotometrically. Additional external DNA standards were used for verification of quantification. The resulting DNA extracts were stored in 25 μl aliquots at -20°C until processing. The extracted genomic DNA (gDNA) was used as template for amplification of the V1-V2 hypervariable regions of the bacterial 16S rRNA gene by PCR using primers S-D-Bact-0008-a-S-16 (27F: 5’AGAGTTTGATCMTGGC 3’) and S-D-Bact-0343-a-A-15 (357R: 5’CTGCTGCCTYCCGTA 3’) [29] with appropriate Illumina adapter sequence. In addition, second set of primers was used to target V6-V7 hypervariable region of bacteria and archaea: S-D-Arch-0519-a-A-16 (5'-CAGCMGCCGCGGTAA-3') [29] and Pro805R (5'-GACTACNVGGGTATCTAATCC-3') [30]. Primers were first in-silico assessed comparatively with other primer sets for their performance in SILVA [31] and Ribosomal Database Project II (RDP II) [32] databases and iteratively improved in order to effectively extend their bacterial and especially archaeal coverage in analyses of anaerobic environments.

All PCR reactions were performed in triplicates. Total volume of 25 μL comprised: 10 ng of template DNA; 12,5 μL NEBNext® High-Fidelity PCR Master Mix (New England Biolabs, USA), 0,75 μl (10 pmol) of each primer and DEPC treated water to 25 μL. PCR cycling conditions included a hotstart (98°C; 5 min), 30 cycles of denaturation (98°C; 10 s), annealing (60°C; 30 s) and elongation (72°C; 30 s) followed by a final elongation step (72°C; 5 min). After PCR samples were purified using Agencourt® AMPure® XP (Beckman Coulter, Inc.). The correct amplicon size was checked on a Bioanalyzer 2100 instrument (Agilent Technologies, USA) using the DNA 7500 kit (Agilent Technologies, USA). The concentration of the purified samples was measured by the Quant-iT PicoGreen kit (Life Technologies, USA). For library preparation the Nextera XT v2 Index kit set A was used (Illumina Inc., USA). The Indexing PCR was performed in 25 μl reactions containing: 12.5 μl NEBNext® High-Fidelity PCR Master Mix (New England Biolabs, USA), 2.5 μl of each Indexing primer, 10 ng of purified amplicons and 6.5 μl DECP treated water. The amplification procedure included an initial denaturation step (98°C; 30 s), 8 cycles of denaturation (98°C; 10 s), annealing (55°C; 30 s) and elongation (72°C; 30 s) followed by a final extension step (72°C; 5 min). The amplicons were first checked on a 1% agarose gel then purified by cutting from a 1% agarose gel and DNA was eluted with the NucleoSpin^®^ Gel and PCR Clean-up (Macherey-Nagel GmbH & Co. KG). Quality and quantity of amplicons were determined as described above. Amplicons were pooled equimolar to 4 nM and sequenced using the MiSeq Reagent kit v3 (600 cycles) (Illumina Inc., USA) for paired end sequencing using an Illumina MiSeq Sequencer. The V1-V2 sequencing run resulted in 5.67 × 10^6^ high quality reads, whereas V6-V7 sequencing run resulted in 8.07 × 10^6^ high quality reads, after chimeric sequences, singletons (< 0.05%) and sequences shorter than 150 bp were removed. Total count of high quality sequences was equivalent to 105300±39200 and 149600±28200 (mean±SD) per sample for V1-V2 and V6-V7 sequencing runs, respectively. All obtained reads were deposited on the MG-RAST database server (http://metagenom-ics.anl.gov/) [33] under accession number (mgp80027; http://metagenomics.anl.gov/linkin.cgi?project=mgp80027).

### Statistical and bioinformatic analyses

Correlation between sequencing of V1-V2 and V6-V7 hypervariable regions was conducted using Mantel test based on respective dissimilarity matrices. To follow the general microbiological and ecological notation, the Bray-Curtis and Morisita-Horn distance [34] were used (R^2^ = 0.58, R^2^ = 0.78, respectively; both p<0.001). Analyses were conducted utilizing data from all samples (as rows) based on genus level sequence abundances of bacteria (as columns), normalized to the equal number of sequences in all samples [34–36] and 9999 permutations of underlying data matrices.

A number of ecological indices were used to assess α-diversity of samples: Taxa_S, Individuals, Dominance_D, Simpson_1-D, Shannon_H, Evenness_e^H/S, Brillouin, Menhinick, Margalef, Equitability_J, Fisher_α, Berger-Parker, Chao-1 (S2 Fig) as implemented in mothur (version 1.35.1) [22]. One-way NP-MANOVA with 10,000 permutations was used to determine significant differences between samples and experimental variants relative to baseline data collection.

To explore the differences between groups of samples, the Bray-Curtis (community membership, species abundance and matching zeroes adjustment), ThetaYC (community membership and relative abundance) and Jaccard (Jclass; community membership) indices were adopted. Three methods testing independent hypotheses, i.e. UniFrac (weighted and unweighted), AMOVA (analyses of molecular variance) and HOMOVA (homogeneity of molecular variance) were used to address specific ecological questions concerning differences between microbial communities as described before (S1 Fig) [35,36].

Multiple-group comparisons were performed using Benjamini-Hochberg false discovery rate (FDR) multiple test correction [37,38]. Permutation tests were conducted using 999 permutations.

The distribution of samples into a defined number of community types (i.e. metacommunities or enterotypes) was assessed using the minimum Laplace value linked with Bayesian and Akaike information criteria [39]. A non-parametric tests LEfSe [40] and metastats [41] as implemented in mothur were used to identify microbial OTUs or genus level taxa that consistently explained the differences between microbial communities. In addition, classical indicator species analysis was conducted as implemented in mothur [42].

The *Firmicutes* to *Bacteroidetes* ratio was calculated for each type of intervention over time in addition to the recently suggested rate of changes over time [43].

Core microbiomes were characterized as fraction of taxa that are found in varying numbers of samples for different minimum relative abundance using Corbata (CORe MicroBiome Analysis Tools) [44]. To measure the difference between taxonomic profiles of two cohorts, a test statistic Abundance-Weighted Kolmogorov-Smirnov (AWKS) was used in order to capture the difference of ubiquities between cohorts across abundances, for each taxon. Major and minor cores were defined and tested for significant differences using AWKS and presented in Abundance-Variability plots that included ubiquity data [44].

Characterization of genus *Bacteroides* sequence data was accomplished using two distinct approaches, Picrust tool [45] to characterize the predicted genus *Bacteroides* related metagenomes and the Ribosomal Database Project Toolbox (http://github.com/rdpstaff/) k-mer search strategy using SequenceMatch utility. First, for analyses within Picrust, sequence IDs belonging to *Bacteroides* were exported from mothur to biom format (http://biom-format.org) [46] and dereplicated using Greengenes 13.5 database (version gg_13_8_99, August 2013) which contains 202,421 bacterial and archaeal sequences. The OTU maps were provided within mothur in order to make the necessary biome file compatible with Picrust tool [45] to predict major functions of *Bacteroides*. Second, in addition to Picrust analysis, k-mer search was conducted using only the high quality sequences of all *Bacteroides* type (T) and cultivated strains. Sequences (length of the 16S rRNA gene > 1200 bp) were retrieved from RDP II database in fasta format. The Ribosomal Database Project Toolbox was used to map *Bacteroides* sequence from this study to sequences of *Bacteroides* retrieved from the databases using k-mer search strategy (n = 7; S_ab_>0.87) using SequenceMatch utility. The distribution of identified strains was linked to existing published physiological and medical reports on particular *Bacteroides* strains.

Metastats [41] was used to identify groups of sequences binned into *Bacteroides* species based on k-mers, that differed significantly between experimental variants (p<0.05).

The variation partitioning approach was used to determine the hierarchy of most important metadata from all measured variables (n = 231) (S3 Table) associated with the dispersion of bacterial communities at 97% OTU and genus levels to determine the extent of explained and stochastic variation in microbial structure. Variables were distributed into three groups: (i) metabolites and immune markers (n = 109), (ii) experimental design (n = 12), and (iii) diet (n = 110) and analyzed in R [34]. A step-down procedure was adopted for each group of variables to test for univariate association of variables with the structure of microbial communities and co-correlated variables were removed from further analyses to decrease dimensionality. This yielded a smaller set of variables in three secondary explanatory matrices significantly associated with microbial communities (n_permutations_ = 5000) that were used in variation partitioning. Heatmaps were constructed using heatmap.2 function as implemented in R package gplots v3.0.1 [47].

## Results

### General microbial diversity and composition are largely unaffected by 21-day inactivity and hypoxia

Unweighted unifrac metrics based either on Bray-Curtis, ThetaYC and Jaccard indices at the level of 97% OTU or genus did not result in significant differences in the microbiome composition between NBR, HBR and HAmb participants of the PlanHab experiment over time (p = 0.16). There were also no significant differences in microbial diversity over time based on analysis of numerous ecological diversity indices (p = 0.44) (S2 Fig). There were no significant differences in enterotype assignment, the *Firmicutes* to *Bacteroidetes* ratio nor in the rate of change in that ratio over 21 days of experiment. The phylogenetic tests used in this study congruently showed that microbial membership at the level of 97% OTU or genus did not differ significantly between experimental variants over the course of the PlanHab study.

**Fig 2:**
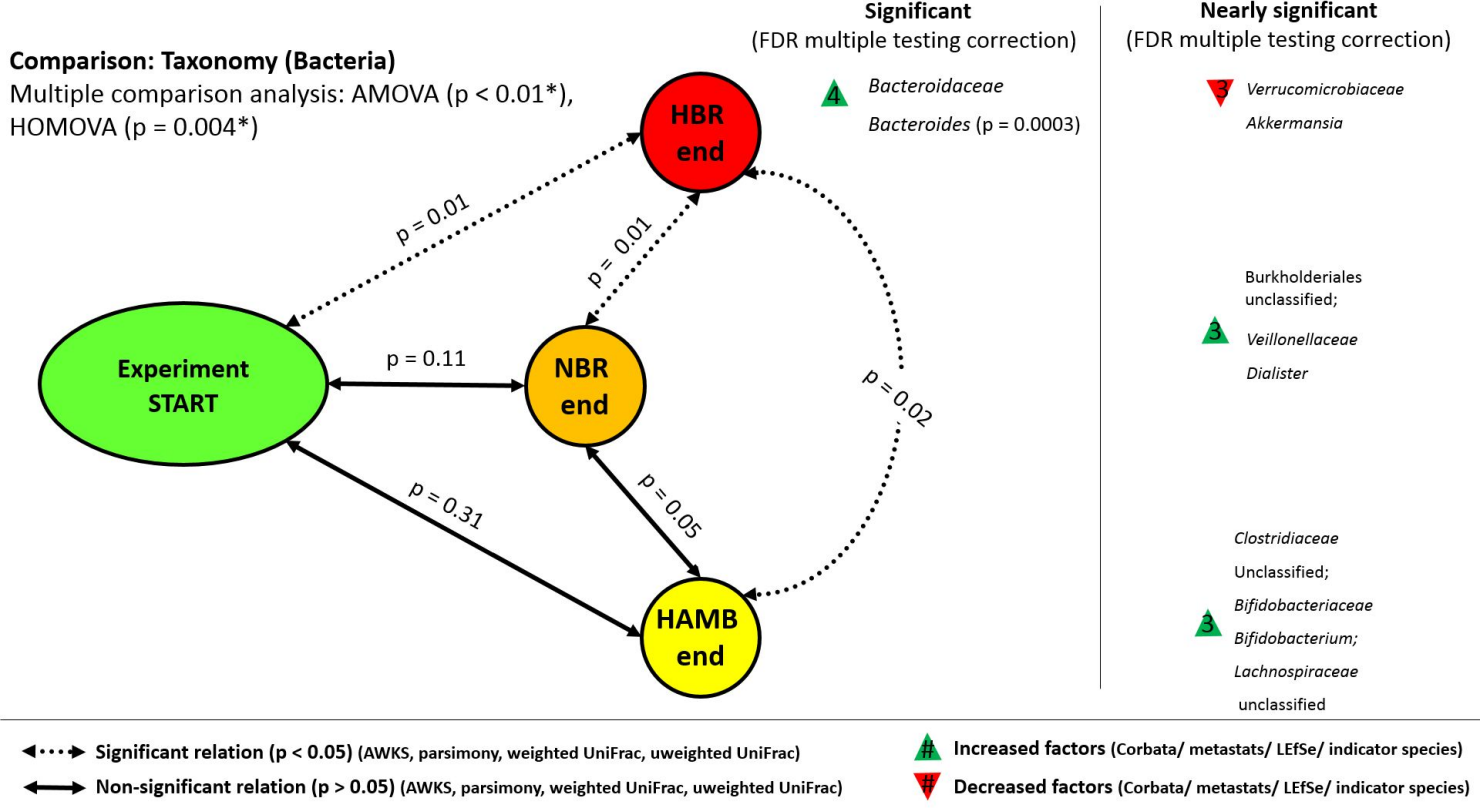
Schematic overview of the detected changes in microbial communities. The congruency of four statistical tests was used to detect significant differences in the structure of microbial community. The additional four statistical tests were used to congruently identify the first responding taxa over the course of the PlanHab experiment. The end-points of the PlanHab experiment with the significant changes are shown (p<0.05, FDR corrected). NBR–normoxic bed rest, HBR–hypoxic bed rest, HAmb–hypoxic ambulatory.

Weighted unifrac, AMOVA and HOMOVA congruently identified that the significant differences in relative abundance, group centroids and group variance size existed between specific groups at the end of experiment after the correction for multiple comparisons (p<0.01, p<0.01 and p<0.004 of tests, respectively). All three tests confirmed that microbiomes of HBR participants became significantly different from NBR and HAmb by the end of the study (p = 0.01, p = 0.01, respectively) (Fig 2). However, microbiomes of NBR and HAmb participants did not differ significantly over the course of PlanHab study (p = 0.11 and p = 0.31, respectively) over multiple time points.

**Fig 3:**
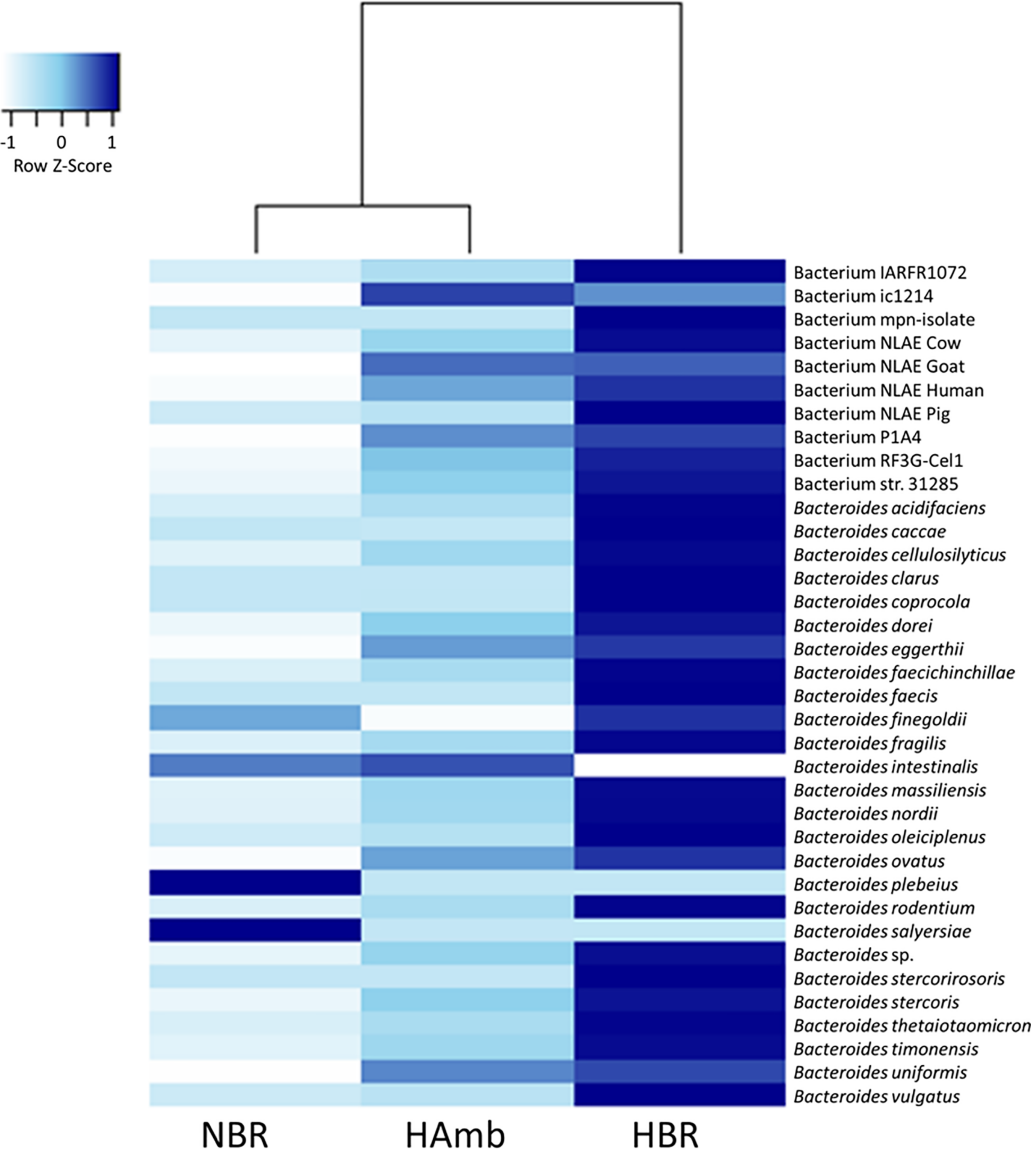
Heatmap plot of the genus *Bacteroides* sequences. NBR, HBR and HAmb sequences were classified at species level and their number normalized to their mean at week four (the end of PlanHab study). The specific and significant increase in the levels of various *Bacteroides* species in HBR can be seen (p<0.05).

Further, the Corbata, LEfSe, metastats and indicator species analyses (Fig 2) congruently identified that members of the genus *Bacteroides*, encompassing many uncultivated strains, became significantly enriched in HBR participants relative to NBR or HAmb (p = 0.0003) in the last week of experiment (Fig 3). However, a large proportion of *Bacteroides* species (75.7%) was shared between experimental groups (S3 Fig) and also matched the physiologically characterized strains described in published literature exhibiting many detrimental and inflammagenic characteristics related to mucin degradation, bile acid resistance, intestinal tract inflammation, opportunistic infections or those preceding autoimmune disorders in type 1 diabetes in youngsters (S3 Table).

Picrust tool was used to impute genus *Bacteroides* related metagenomes based on the availability of 16S rRNA sequences from sequenced genomes. *Bacteroides* collection of full genomes is available at Joint Genome Institute Integrated Microbial Genomes & Microbiomes (JGI IGM/M; http://img.jgi.doe.gov/cgi-bin/m/main.cgi) (n_Genus =_ 275 genomes JGI/IGM). Most of the listed species (n = 61) contained up to two genomes, in contrast to the few most well and easiest to culture (n = 16), that covered 72.7% of all *Bacteroides* genomes. In addition, 33.8% of *Bacteroides* genomes were represented by single species whereas 60% of genomes were represented by 6 *Bacteroides* species only. The obtained Picrust tool derivatives describing the imputed *Bacteroides* metagenomes were not informative, i.e. did not differ significantly, reflecting the low coverage of *Bacteroides* intragenomic diversity within the genome sequence databases.

**Fig 4:**
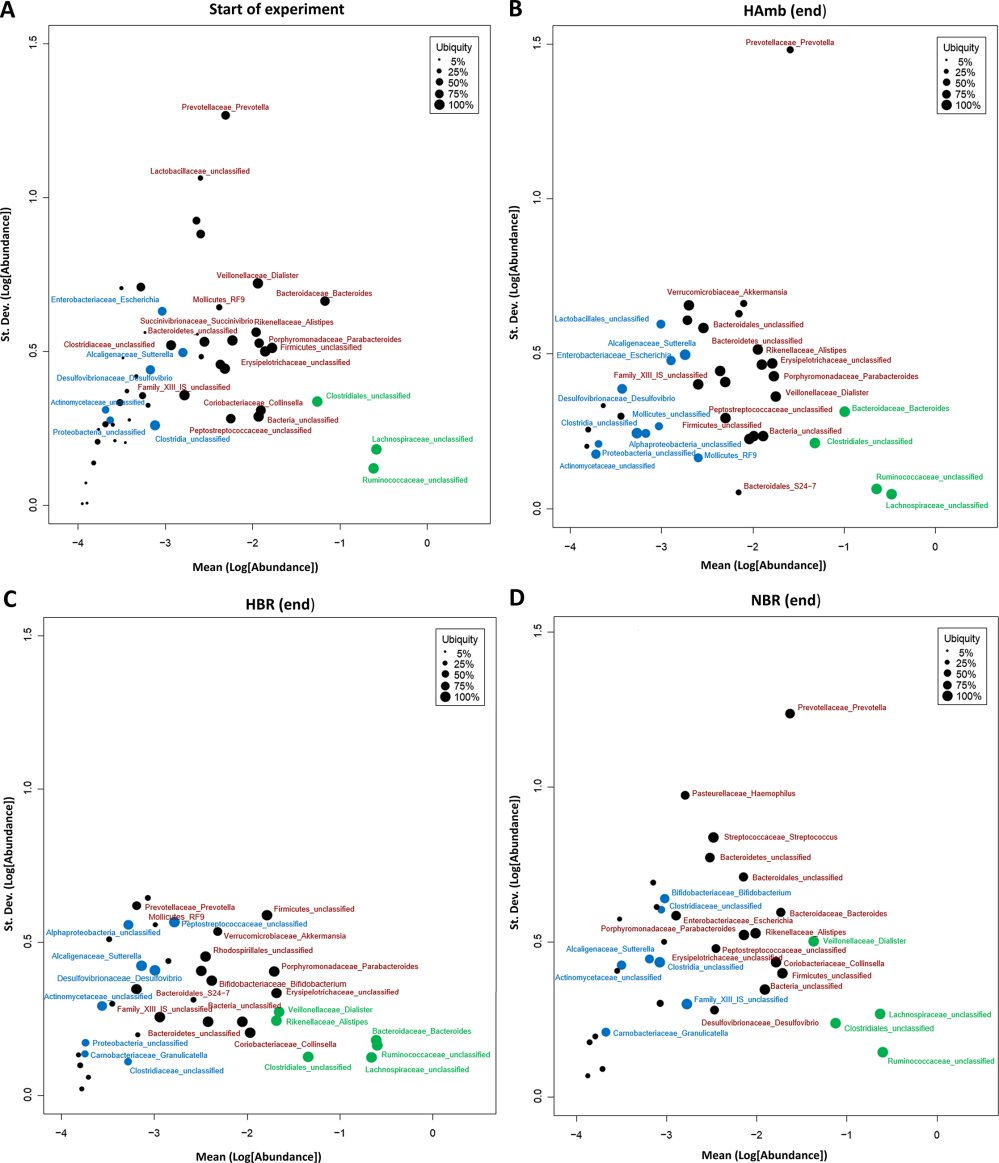
The overview of core microbiomes at the start-up and endpoint of the PlanHab experiment. The Corbata plots of abundance (x-axis), variability (y-axis) and ubiquity (circles). Each circle represents a single taxon with its size proportional to the ubiquity of the taxon, thus the larger the circle, the more ubiquitous the taxon across the cohort. Taxa were classified and divided into major (green) and minor (blue) core microbiomes next to other bacterial taxa unclassified as either major or minor core (black). Taxa towards the top of the plot have greater variation. Abundance increases towards the right of the plot. **(A)** Microbiomes at start-up of PlanHab experiment; **(B)** hypoxic ambulatory (HAmb); **(C)** hypoxic bed rest (HBR); and **(D)** normoxic bed rest (NBR).

The composition of major and minor cores of microbiomes as identified by abundance-variability-ubiquity analysis in Corbata (Fig 4) largely corresponded to the microbiome composition observed in Human Microbiome Project (HMP) [48]. The limited detection of Archaea by V6-V7 prokaryote primer set (Archaea: Bacteria ratio < 1: 10^4^) in this study further supports this observation. The most abundant phyla were *Firmicutes* and *Bacteroidetes*, with the family of *Prevotellaceae* showing the highest variability. Other bacterial taxa apparently responded to inactivity and hypoxia over the 21-day experiment (Fig 2). However, because of inter-individual variation over the 21-day study, these shifts did not reach statistical significance with all four statistical tests (p>0.05) (Fig 2,) used for identification of taxa that responded differed significantly between the variants (S1 Fig). For example, members of the genus *Eubacterium*, a commensal causing opportunistic infections of soft tissues were increased in HBR participants, whereas mucus dwelling *Akkermansia* were reduced. Opportunistic, proinflammatory members of *Dialister* characteristics of pre-type 2 diabetes were apparently enriched in NBR participants. In contrast, members of the probiotic genus *Bifidobacterium* were enriched in phenotypically healthy HAmb over time.

### Intestinal environmental parameters are congruent with human physiology markers

**Fig 5:**
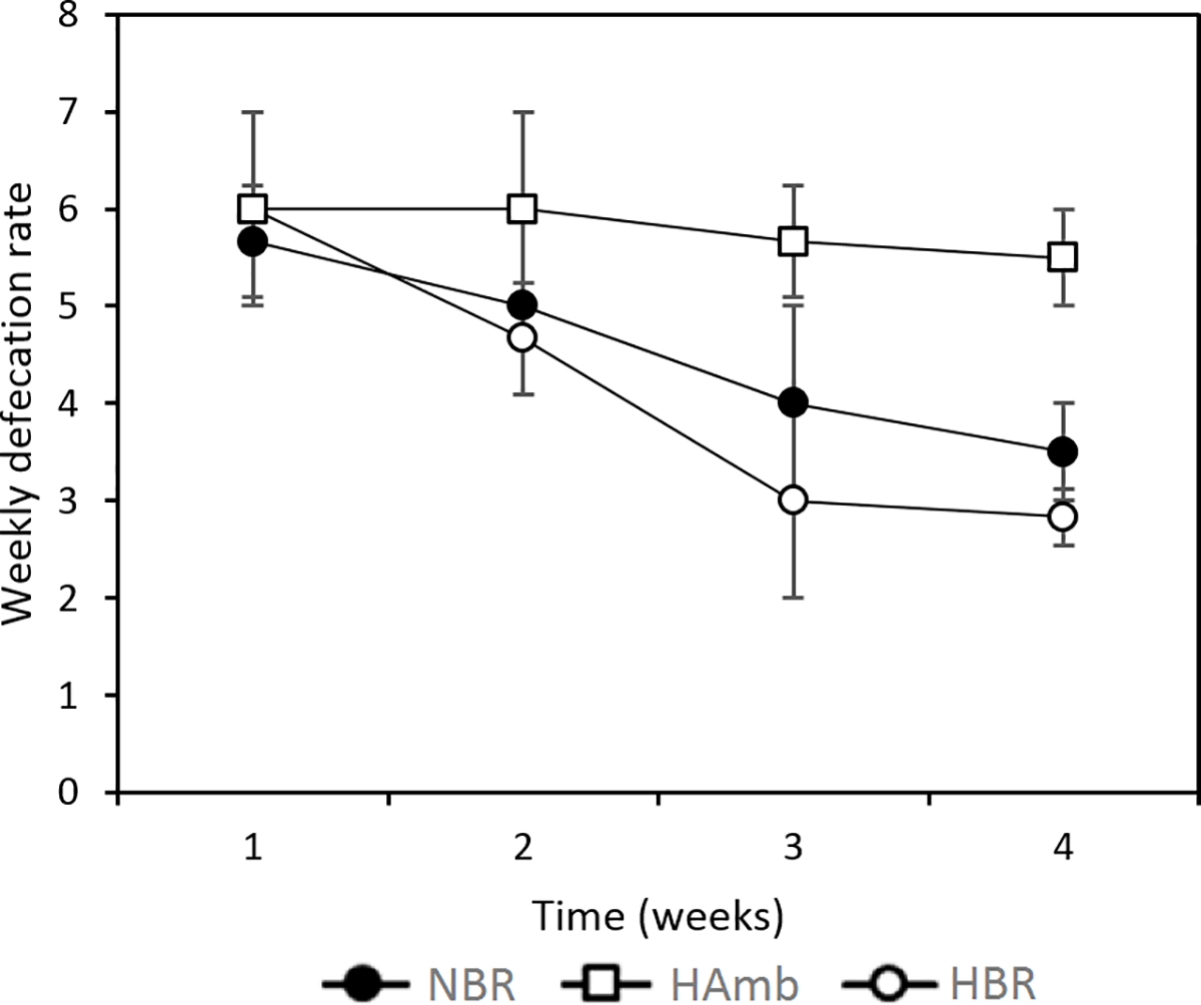
Weekly defecation rates as a direct measure of constipation due to increased retention times of organic matter in intestinal system throughout the PlanHab experiment.

**Fig 6:**
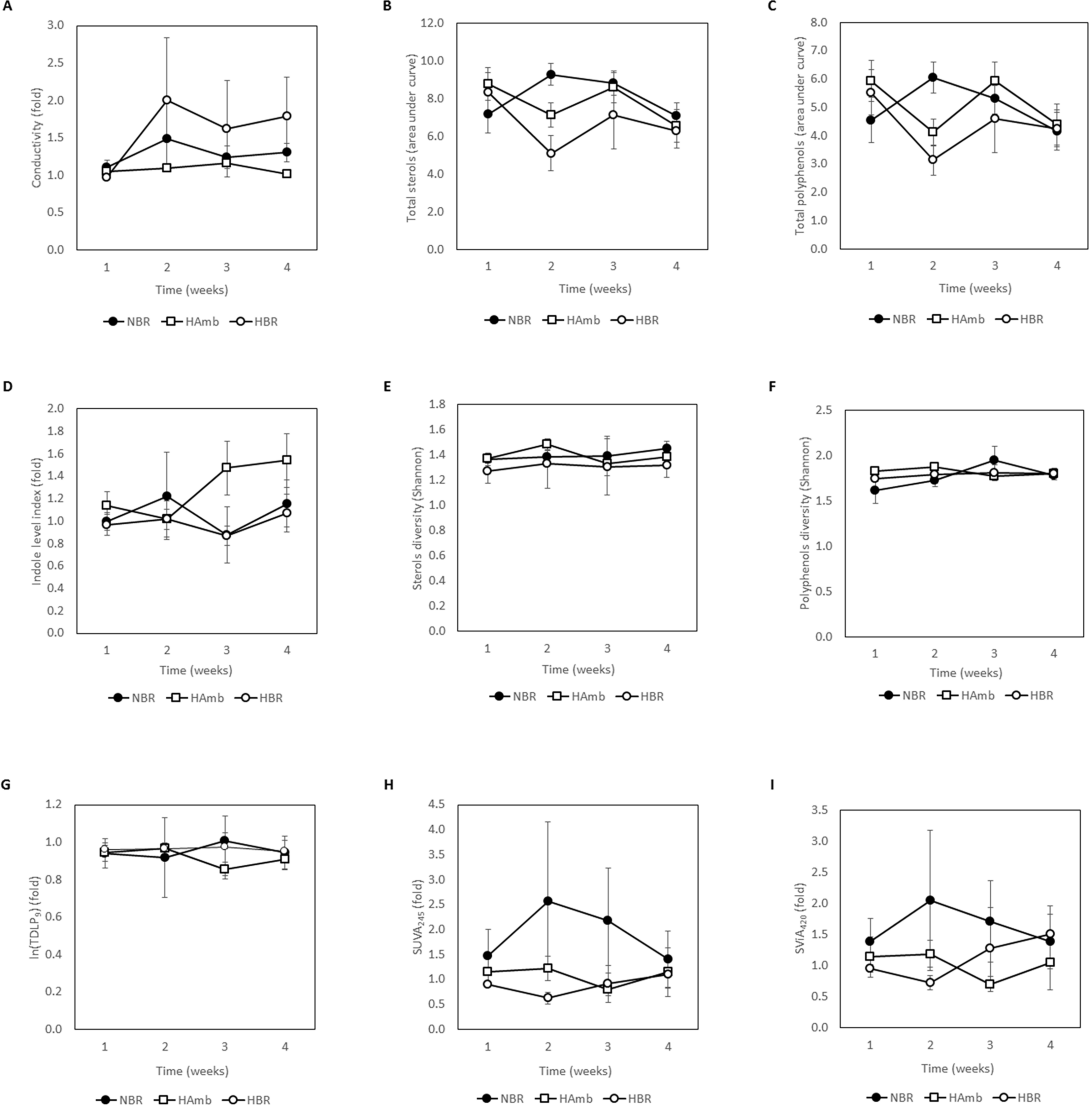
Fluctuations in the intestinal parameters over the course of PlanHab experiment. (A) Electrical conductivity of fecal matter as a measure of ionic strength of intestinal environment; (B) total quantities of sterols, and (C) polyphenols; (D) indole level index; (E) diversity of sterol and (F) polyphenol peaks; (G) the sum of nine *p*-hydroxy, vanillyl, and syringyl phenols; (H) specific ultraviolet absorbance (SUVA) of dissolved organic matter (DOM) as a measure of overall DOM aromaticity; and (I) specific visual absorbance (SViA) index as a measure of colored DOM.

A number of novel parameters not considered before were explored in this study to describe gut environment and its biochemical characteristics over the course of 21-day experiment and their relationship to existing data from past PlanHab sub-studies was explored. The frequency of defecation as the number of defecation events per week progressively decreased over the course of bed rest in NBR and was more pronounced in HBR under hypoxia (Fig 5). The defecation rates were also highly correlated to Bristol stool scale (BSS) and weekly retention time in addition to bile acid content (R^2^>0.85) reported before for the same participants [13]. IEC of fecal matter, a robust measure ionic strength and hence of innate immune system activity, increased within the first week of bed rest in HBR and NBR participants for 67% and 32%, respectively (Fig 6A) (p<0.05). In contrast, IEC of HAmb participants remained stable despite the synchronized and controlled diet in all variants. The increased values of IEC were also highly correlated to a number of negative human physiology symptoms recorded before [14,16] (e.g. insulin insensitivity) and intestinal parameters such as constipation (BSS), weekly retention time, bile acids (BA) and eosinophil derived neurotoxin (EDN) (R^2^>0.83) reported in our recent study [13].

The concentration and chemical diversity of sterols and polyphenols reported in this study were not significantly affected over time based on non-parametric PERMANOVA (p>0.25) (Fig 6). Total polyphenol content was only transiently increased in NBR participants with large fluctuations between participants whereas remained largely stable in HBR and HAmb. Detailed analyses of dissolved organic matter (DOM) spectral data revealed that the sum of nine p-hydroxy, vanillyl, and syringyl phenols (TDLP_9_), levels of DOM aromaticity (specific ultraviolet absorption (SUVA)) and colored DOM (specific visual absorption (SViA)) next to the index of anaerobic production of tannin-like compounds were also not significantly different between the experimental variants over time. In contrast, the levels of indole, a microbial derivative of essential aromatic amino acid tryptophan ((2S)-2-amino-3-(1H-indol-3-yl)propanoic acid) and a precursor for simple indole alkaloids such as melatonin and serotonin, were found to increase significantly over time in HAmb in comparison to NBR and HBR (Fig 6D).

**Fig 7:**
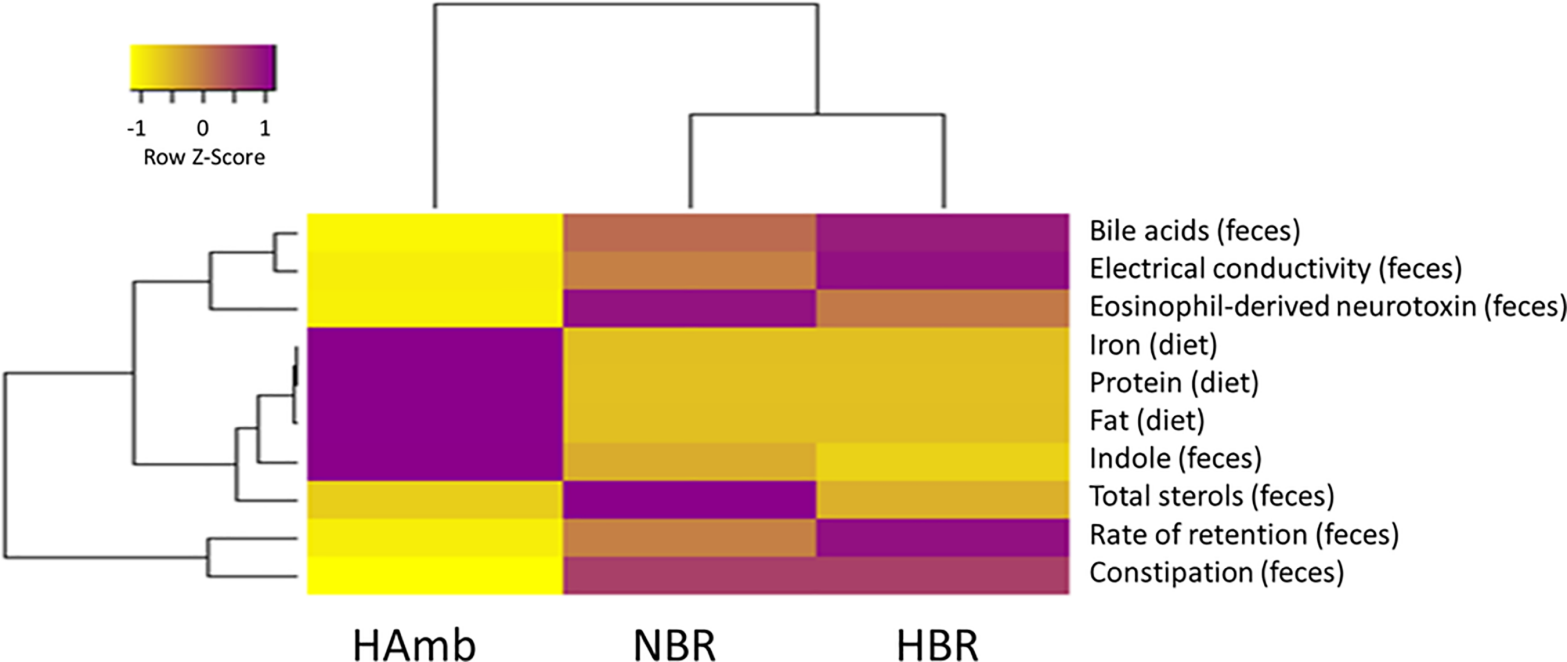
Heatmap plot showing the relationship between parameters describing intestinal environment that differed significantly by week four at the end of PlanHab experiments (n = 10; p<0.05, FDR corrected). See S2 Table for details on all measured variables describing diet, experimental conditions, intestinal environment, metabolites and immune parameters (n = 231) that are part of the new in-house PlanHab database [13–19].

**Fig 8:**
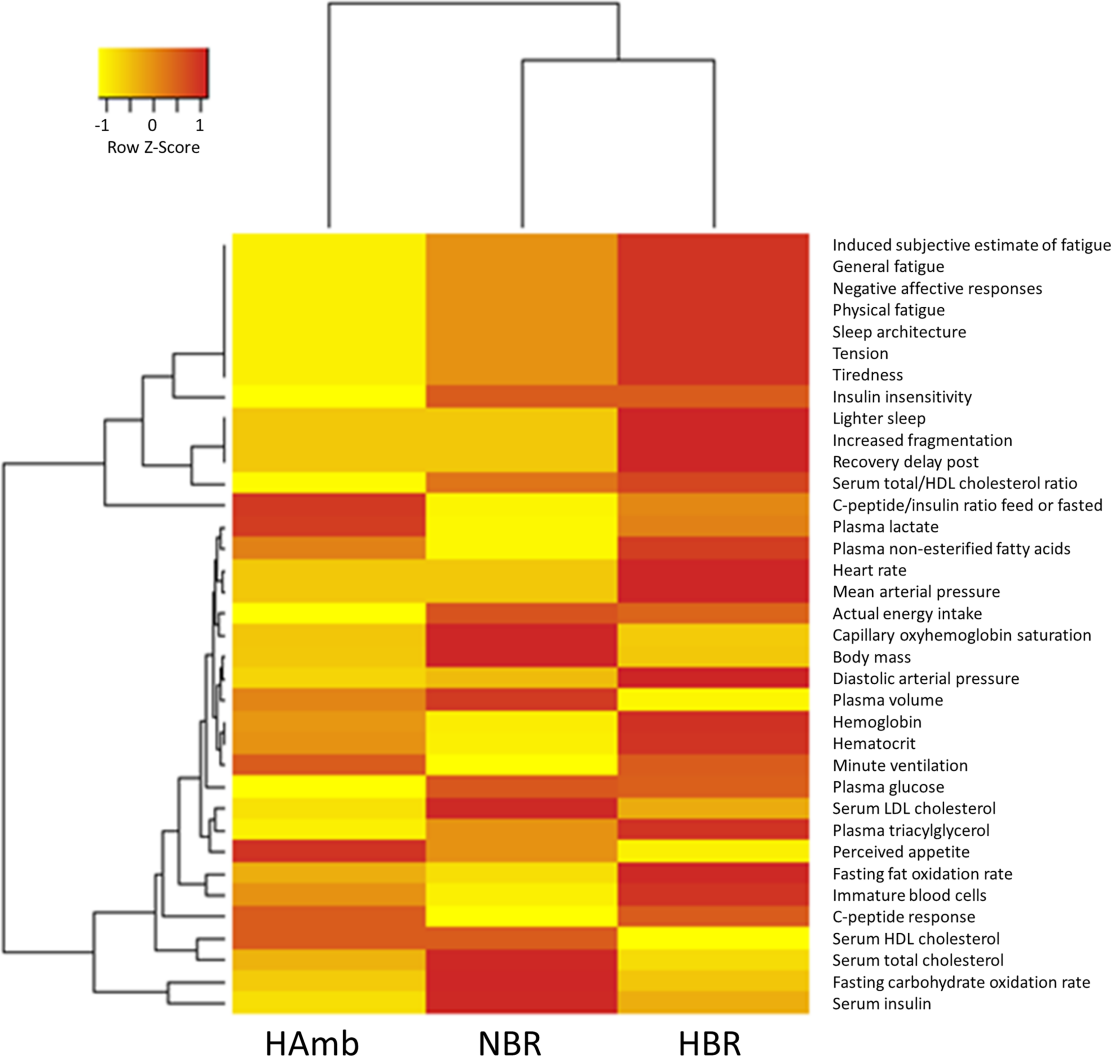
Heatmap plot showing the relationship between parameters describing human physiology that differed significantly by week four at the end of PlanHab experiments (n = 36; p<0.05; FDR corrected). The parameters recorded in this and past PlanHab substudies are now part of the new in-house PlanHab database [13–19].

In order to integrate variables recorded in this and past sub-studies of all participants throughout the run-in and experimental phase an in-house PlanHab database was established in this study (S2 Table). This enabled us to identify parameters that differed significantly between the experimental variants over the course of experiment and link them to bacterial community structure. Ten parameters out of 231 describing diet, intestinal metabolites, immune and chemical parameters (Fig 7) and 36 markers describing human physiology were significantly different between NBR, HBR and HAmb before the week four of PlanHab experiments (p<0.05; Fig 8). Covariation of the two datasets increased progressively with the time spent in the PlanHab experiment (linear regression R^2^ = 0.73) confirming that the two datasets were highly correlated (Mantel test, R^2 =^ 0.978; p<0.003).

### Identification of the key structuring parameters

To determine the association between microbiome data and the three data matrices describing (i) diet, (ii) experiment, (iii) intestinal metabolites, immune and chemical parameters assembled in the PlanHab database (S2 Table), two variation partitioning analyses were conducted at two different levels of resolution (97% OTU and Genus). Despite the introduction of numerous variables into the two analyses, many of the metadata variables were found to be correlated, and were hence removed during the step-down procedure, or were at the threshold of association of a completely random test variable, and were hence excluded from further analyses. A smaller subset of variables significantly associated with the dispersion of bacterial communities was identified as the key parameters explaining significantly the distribution of microbial taxa over time and experimental setup (S4 Table). The important overarching parameters were characteristics of individual participants and interactive effects of hypoxia and inactivity (experiment), short chain fatty acids and immune system variables (intestinal parameters) next to dietary water, fats and proteins. The three matrices ((i) intestinal metabolite and inflammation markers, (ii) PlanHab experimental setup, and (iii) experimentally structured metabolites) used in variation partitioning at the level of 97% OTU, explained 27.4%, 17% and 7.1% of variability, respectively. Hence, 48.5% of variation in microbial community data remained unexplained. In contrast, variation partitioning using the same three matrices at the genus level showed that the same categories explained slightly lower proportion of variability (21.4%, 12.8% and 1.7%, respectively), leaving 65.8% of variability unexplained. The extent of unexplained variation at 97% OTU and genus levels was consequently attributed to a combination of: (i) experimental sources of variation, (ii) unknown real sources of variation and (iii) random noise. The progressively increasing extent of phylogenetic resolution (phylum to species levels, or 80 to 99% OTU) also increased the extent of explained variation, providing a rationale for detailed functional metagenomic analyses targeting subspecies genomic diversity.

## Discussion

### The extent of experimental control within the PlanHab study reveals imminent shifts in food preference and human physiology

PlanHab experimental setup was tuned to control the effects of factors, that have been identified to contribute to changes in microbial community structure: host lifestyle and diet [49,50]; diurnal oscillations [51]; exercise [3,4] and development [52]. The diet supplied to participants in this study was balanced and synchronized to provide feeding habit continuation and resembled the habitual menu composition of participants [14]. Differences in cyclic circadian oscillations, known to induce transkingdom control of microbiota diurnal oscillations that promote metabolic homeostasis [51] were minimized through the adoption of 16/8 daylight regime in this study [14]. In addition, the timing of food delivery was kept constant in order to minimize variation in flow-rate of material through the gut that was previously shown to influence microbial and host metabolic activities [14,53]. The prescreening of participants according to NASA/ESA SOP yielded healthy microbiomes (Fig 4A) that corresponded to those described as healthy in HMP [48].

Consequently, the PlanHab study enabled us to monitor the effects of inactivity and hypoxia on many vital subsystems of human body over time. Exercise clearly led to modified food preference in HAmb. The amounts of ingested iron, proteins, fat and Ca^2+^ were 9%, 10%, 6% and 7.5% significantly higher in HAmb in comparison to HBR and NBR [14], an observation mirroring the diet composition of professional athletes reported recently in a study employing end-point groups without controlled diet [3]. Hypoxia alone did not induce significant differences in feeding behavior between NBR and HBR. In contrast, the acute cessation of exercise in NBR and HBR induced significant changes in human physiology that were further aggravated by hypoxia in HBR. HAmb members exhibited lower blood glucose, retained (high) insulin sensitivity, reduced body fat, lower postprandial glucose, fasting serum total cholesterol and LDL cholesterol [54], next to unchanged advanced oxidation protein products and nitrotyrosine, and 30–53% increased superoxide dismutase, catalase, plasma ferric reducing antioxidant power. HAmb exhibited normal BSS types, fecal retention times, defecation frequency (Fig 5), IEC (Fig 6) and inflammation markers (EDN, BA) in intestinal tract in comparison to HBR and NBR [13], indicating a healthy physiological state in HAmb despite the negative effects of hypoxia evident in inactive HBR participants.

Hypoxia was associated with 10% reduced capillary oxyhemoglobin saturation in HBR and HAmb [21]. However, only HBR exhibited 10% increased mean arterial pressure, 15% increased heart rate and three times larger decrease in plasma volume with concomitant three times larger increase in mature blood cells, hemoglobin, hematocrit than NBR or HAmb. General fatigue, tiredness, tension, recovery delay, negative affective responses, induced subjectiveness were also largest in HBR, followed by NBR, whereas almost undetectable at HAmb despite equal hypoxia levels as in HBR [18]. Increased postprandial glucose concentration and reduced insulin sensitivity were observed in NBR and HBR, suggesting that the negative effects observed in human physiology were dose dependent on the time subjected to inactivity and additional hypoxia [15].The significant changes in a number of intestinal parameters that were observed during the second week (defecation frequency (Fig 5), IEC (Fig 6), constipation and increased weekly retention time [13]) or third week (indole (Fig 6), EDN, BA [13]) corroborate dose dependent response of human physiology to cessation of exercise [13]. However, significant changes in bacterial community structure were not detected, or were delayed until week four in HBR only. Inactivity (bed rest) and hypoxia-responsive characteristics of the host (e.g. IEC and constipation) were apparently rather rapidly translated into modified microbial metabolism (e.g. indole), taking advantage of intestinal abrasions and anoxic ischemia [13], resulting in increased levels of *Bacteroides* in HBR, accompanied by responses of innate and adaptive immune systems (EDN, BA) [13,55,56].

The increasing levels of indole as a microbe-generated signal substance in HAmb variant coincided with healthy host physilogy in HAmb. This shows that the positive effects of rather limited bouts of exercise exerted on host as well as the microbiome were linked. Indole was shown to function as a quorum-sensing signal that regulates the virulence and biofilm formation of enterohemorrhagic *E*. *coli*, *Pseudomonas* and other commensal bacteria, but also strengthens the barrier function of the mucous membrane by repairing tight junctions (S3 Table). Hence, it is plausible that most relevant changes for the host physiological status took place at the level of molecular and chemical cross-talk between microbes and between microbes and the host.

### Resilience in microbial intestinal system towards short-term inactivity

The lack of directed changes in intestinal microbiome over the course of the PlanHab experiment points to a lag in response of microbial communities or possibly to an evolutionary resilience in microbial intestinal system towards short-term inactivity [13] despite accompanying significant changes in intestinal environmental parameters. A major factor shaping the balance between different human bacterial lineages is the ability of each group to compete efficiently for complex nutrients delivered to the intestinal system in spatial and temporal pattern [57,58]. Experimental physical inactivity effectively modified intestinal conditions through increased constipation (Fig 5), modified intestinal parameters (Fig 6) and autonomous shift in diet (Fig 7) over the course of PlanHab experiment [14]. The assembled data on increased levels of the genus *Bacteroides* in HBR in this study support recently described generalization [57,58], that the same microbial taxa responded to modified environmental conditions provided by the host by flexibly adjusting their metabolic activities through gene expression leading to significant increase in their relative abundance and internal diversity. In addition, this study for the first time showed that exercise per se effectively modified and maintained autonomous feeding habits towards significantly increased ingestion of proteins and fats in HAmb relative to HBR or NBR, in absence of systemic inflammation or significant changes in microbial community structure or diversity. This finding supports recent observation that professional athletes exhibited lower levels of *Bacteroides* and increased microbial diversity at higher dietary protein and fat intake than control groups [3]. HAmb exhibited a healthy phenotype [13–15], despite the fact that most of the *Bacteroides* species observed in HBR were also present in NBR and HAmb at lower levels (Fig 3). In addition, HAmb, NBR and HBR groups of PlanHab study contained at least 28.8%, 35.8% and 21.2% lower fraction of *Bacteroidetes*, respectively, than the athletes, low BMI and high BMI controls, reported before, respectively [3]. This clearly shows that related species and microbes can have multiple, very different or even opposing effects on host metabolism and health [58] based on microbial metabolic responses to the detected changes in the multivariate environment of intestinal tract. Consequently, for the development of the observed pathologies in host physiology other conditions than the sole presence or abundance of particular microbial type are necessary making the microbial metabolites the key effectors and mediators of signal for the real-time modification of host physiology.

### Changes in environmental parameters shape microbial activities

Numerous characteristics of intestinal environment (S2 Table) that were measured in this and our past study [13] revealed that many of these variables were either correlated and also covaried over the course of experiment, indicating a substantial and potentially hierarchical codependence between parameters within intestinal environment. In addition, the majority of variables did not change significantly, indicating that the extent of experimental disturbance (inactivity, time, hypoxia) and their interactive effects were not sufficient to induce significant changes, as observed for sterol and polyphenol content and diversity next to DOM spectral derivatives in this study. Dietary polyphenols are a major source of antioxidants consumed by humans. Polyphenols possess not only antioxidant properties but also antiviral, antibacterial, antiinflammatory and anticarcinogenic effects, as well as the ability to modulate certain signaling pathways such as nuclear factor-κB activation. Sterols as lipids have important biological functions in humans depending on the interaction between the diet of the host (that determines the relative quantities of sterol precursors) and the complex metabolic transformations of sterols by intestinal microbiota to their secondary derivatives with potent biological effects [4–8].

Constipation (Fig 5) in response to inactivity effectively changed IEC that is linked to the effectiveness of microbial nutrient transport (Fig 6), mucin characteristics (folding, thickness and porosity) and consequently surface availability of tripartite complex of glycans derived from diet (plant polymers), host (mucin-O-glycans) and microorganisms (extracellular polymeric substances) [58–60].

In line with these observations, the major factor shaping the metabolism of intestinal communities, including *Bacteroides*, is competition for nutrients. This competition is paralleled by the simultaneous need to shield themselves from harmful environmental factors, innate and adaptive parts of immune system, through coordinated modulation of capsular polysaccharide biosynthesis and surface antigenicity [61,62]. The capacity of *Bacteroides* to respond to environmental change within the timescale of minutes [58] is supported by the coordinated regulation of nutrient intake and surface antigenicity, the general prioritization of available plant polymers over host mucus and the dynamic responses in expression of genes sustaining metabolic hierarchy of available resources on three levels: enzymatic, transport and regulatory [61,62]. In addition, the ability to transfer rapidly evolving genomic elements in isogenic *Bacteroides* strains confirms the observed metabolic plasticity [63]. It follows that the observed negative physiological symptoms of the host in this controlled study were the result of modified microbial activities in response to environmental disturbance due to host inactivity rather than direct community disturbance by inflow of novel microbial cells with food and water as was suggested before [10,11,49,51,64].

### Reversibility of physiological symptoms as a part of inbuilt mammalian physiology

The regularly observed increased inflammatory responses in obesity related comorbidities were recently linked to central inflammatory mediators nuclear kappa B (NF-κB) and hypoxia inducible factor 1 (HIF-1) [65,66], that modulate cell transcription, shape nutritional-immunity status of the gut and induce the release of reactive oxygen and nitrogen species (RONS) [67]. However, increased levels of NF-κB and RONS are found in intestinal mucosa during intestinal colonization after birth [52], in inactivity-obesity related syndromes [1,2] and in various hibernating mammals during their torpor cycle [65,66,68], indicating a significant interplay of microbiota and intestinal mucosa driving immune homeostasis of the hosts. Phylogenetically distant mammalian species were shown to share similar mechanisms of network control of the same orthologous genes during physical inactivity [69], including humans as controls. The characteristics of inactive phenotype observed in 21-day PlanHab experiment in NBR and HBR variants corresponded surprisingly well to the hibernation characteristics of mammals during their general 28-day torpor cycle. The list of adaptations included elevated levels of genus *Bacteroides*, increased constipation and insulin resistance, lipid metabolism, triglyceride and glucose concentration, total cholesterol, even-acyl carnitines next to increased red blood cells, hemoglobin and hematocrit and increased neutrophils, lymphocytes and monocytes, C-reactive protein and other [70,71]. In contrast, other negative physiological characteristics of NBR and HBR matched those observed during seasonal overeating (hyperphagia) in the same mammals (e.g. increased bile acid content, adiposity). This shows that physical inactivity and food availability acted as two separate overarching signals governing gene regulation and expression of the mammalian host [69].

Adiposity in humans, however, was associated with reduced insulin sensitivity and accompanied by constant daily overeating and inactive sedentary lifestyle with limited or no exercise as observed in captive primates [1,2]. The fact that mammalian hibernators returned to normal physiological status including insulin sensitivity during the physically active part of seasonal cycle despite overeating [68,70,71] is complementary to the alleviation of inactivity-generated negative physiological and psychological symptoms observed in NBR and HBR over 5 and 14 days, respectively, during the PlanHab wash-out period [13–15]. This period was characterized by increased (voluntary) exercise, establishment of hydrostatic gradients within body [14,16] leading to reintroduction of abdominal splanchnic circulation [72], significantly increased physiologic demands of the host, alleviated constipation [14], indicating that signals from exercise and muscle turnover reached intestinal tract as suggested before for athletes [4], animal models [73–76] and potentially primed functional capacity of healthy microbiome (this study (IEC, indole)).

Short-term inactivity thus appears to be completely reversible as part of inbuilt mammalian physiology [14,16]. However, interactive and dose dependent responses to extensive physical inactivity and overeating, previously not encountered in the human evolution, lead to progressively significant system deconditioning, physiologic, immune and psychologic comorbidities observed in primates in captivity and humans indulging in Western lifestyle over prolonged periods (S4 Fig) [1,2].

### Limitations

A few limitations and concepts of this study need to be considered as well [13]. Firstly, the sample size in this study was relatively small, but well within the limits of recent detailed studies [49,51,64]. Second, the limited statistical power and accompanying potential for type-II error was at least partly alleviated by the fact that the test participant population was prescreened for healthy young males according to SOP used by ESA/NASA and the study executed according to SOP for NASA bed rest studies. Nevertheless, a larger study sampling random population longitudinally as participants experience variations in intestinal transit times might be able to find additional and more significant effects. Third, the inclusion of female participants would definitely add a much needed layer of complexity. Four, many of the initially measured variables were found to be correlated and hence did not contribute to the partitioning of variability in microbial communities. Next, the use of 16S rRNA genes has limited capacity for linking the phylogenetic information with microbial physiology. For the scope of detecting more subtle changes in microbial community structure at the level of bacteria, archaea, protozoa, fungi and also functional genes, the analyses adopting shot-gun metagenomics approaches would be even better suited. Moreover, novel draft genome sequences of *Bacteroides* species reconstructed from sequenced metagenomes would help explore the genetic plasticity and pathogenicity potential of this genus. Last, measurements of additional variables and possibly at different scales, collected continuously and noninvasively could decrease the extent of unexplained variation. In this respect the developed approaches of full-scale metabolomic analyses of fecal content have the potential to increase the number of metabolites and provide means for chemical identification of novel compounds present in significantly different concentrations in intestinal tract.

## Conclusisons

The results of our study show that cessation of exercise resulted in progressive decrease in defecation frequency and concomitant increase in IEC. The transition from healthy physiological state towards the developed symptoms of low magnitude obesity-related syndromes was dose dependent on the extent of time spent in inactivity and preceded or took place in absence of significant rearrangements in bacterial microbial community. The first significantly enriched taxa were probably inflammagenic and mucin degrading species *B*. *thetaiotamicron*, *B*. *acidifaciens*, *B*. *fragilis*, *B*.*dorei* and other members of the genus *Bacteroides* in HBR variant characterized with the most severe inflammation symptoms, indicating a shift towards host mucin degradation and harmful immune crosstalk. The inactivity-generated negative physiological and psychological symptoms diminished after reintroduction of exercise in NBR and HBR over 5 and 14 days, respectively, and may be related to the cyclic energy-conservation mechanisms evolutionary shared among mammals.

## Supporting information

S1 Fig Schematic representation of experimental outline used in PlanHab study (http://cordis.europa.eu/project/rcn/104127_en.html). **(A)** The parameters (hypoxia and exercise) and the resulting status of host physiology after 21 days in controlled experiment. **(B)** Human systems biology exploration space mapping exercise with oxygen saturation levels and human population diversity.^[^^1^^–^^4^^]^ **(C)** The difference between accumulation and short-term real-time experiments. The inset shows a tentative scheme of dose dependent benefits derived from various levels of exercise.^[^^4^^–^^8^^]^ **(D)** Methodological overview.
(PDF)

S2 Fig Ecological indices used to assess α-diversity of bacterial microbiomes at the start-up and endpoints of the PlanHab experiment. Taxa_S, Individuals, Dominance_D, Simpson_1-D, Shannon_H, Evenness_e^H/S, Brillouin, Menhinick, Margalef, Equitability_J, Fisher_α, Berger-Parker, Chao-1.
(PDF)

S3 Fig Strain level deconvolution of the genus *Bacteroides* sequences found in NBR, HBR and HAmb variants at the end of the PlanHab experiments. The overall significant increase in various strains of *Bacteroides* at the end of PlanHab experiment in HBR is shown (p < 0.05).
(PDF)

S4 Fig A schematic presentation of interactive and dose dependent responses to physical inactivity and overeating over the course of human evolution. (PDF)

S1 Table Baseline demographic and clinical characteristics for each experiment group (mean ± SD) in PlanHab experiment. (PDF)

S2 Table Overview of new in-house PlanHab database. The three data matrices containing (i) experimental, (ii) diet, and (iii) metabolite and immune status related parameters (n = 231) were used in variation partitioning of bacterial microbial community datasets in this study.
(PDF)

S3 Table Characteristics of identified Bacteroides species. Identification of *Bacteroides* species at the end of PlanHab experiment with their mucin degrading and inflammogenic characteristics of medical relevance reconstructed from published literature. The k-mer search strategy (RDPTools; n = 7) with Sab score>0.87 to type and cultivated strains with reported relevance for dysbiotic medical conditions and described metabolic characteristics was used in this analysis.[9–17]
(PDF)

S4 Table Variables significantly associated with the distribution of bacterial community structure in PlanHab experiment at the level of 97% OTUs and genus. The data were retrieved from the newly established in-house PlanHab database and used in variation partitioning. Please see Materials and methods next to S2 Table for additional information. A1AT–Alpha1 antitrypsin; TSOC–total soluble organic carbon, EDN–eosinophil derived neurotoxin; BSS–Bristol stool scale; polyphenol a1, b1, a3 –polyphenol peaks with currently unknown chemical structure.
(PDF)

S1 Text PlanHab metodology. (PDF)

S2 Text CONSORT checklist. (PDF)
