# Supplementary material for: Hypoxia and inactivity related physiological changes precede or take place in absence of significant rearrangements in bacterial community structure: The PlanHab randomized trial pilot study
Source: PLoS One. 2017 Dec 6;12(12):e0188556. doi: 10.1371/journal.pone.0188556 (PMC5718606; doi:10.1371/journal.pone.0188556)
Supplement: S1 Fig — (A) The parameters (hypoxia and exercise) and the resulting status of host physiology after 21 days in controlled experiment. (B) Human systems biology exploration space mapping exercise with oxygen saturation levels and human population diversity.[1–4] (C) The difference between accumulation and short-term real-time experiments. The inset shows a tentative scheme of dose dependent benefits derived from various levels of exercise.[4–8] (D) Methodological overview. (PDF) [file pone.0188556.s001.pdf]

S1 Figs. Schematic representation of experimental outline used in PlanHab study (http://cordis.europa.eu/project/rcn/104127_en.html). (A) The parameters (hypoxia and exercise) and the resulting status of host physiology after 21 days in controlled experiment. **(B)** Human systems biology exploration space mapping exercise with oxygen saturation levels and human population diversity.[1–4] **(C)** The difference between accumulation and short-term real-time experiments. The inset shows a tentative scheme of dose dependent benefits derived from various levels of exercise.[4–8] **(D)** Methodological overview.

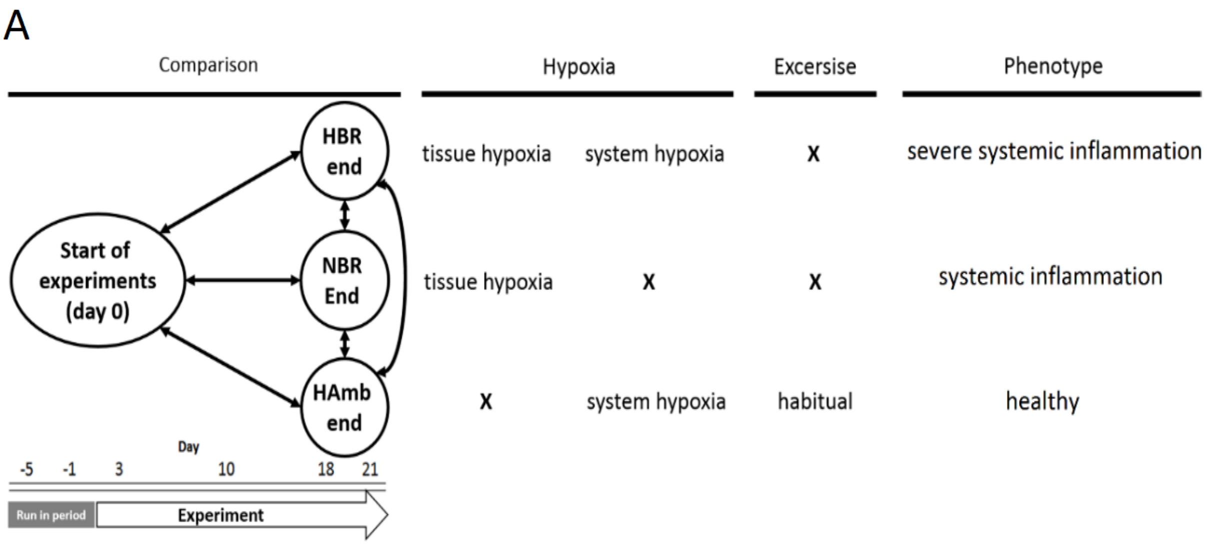

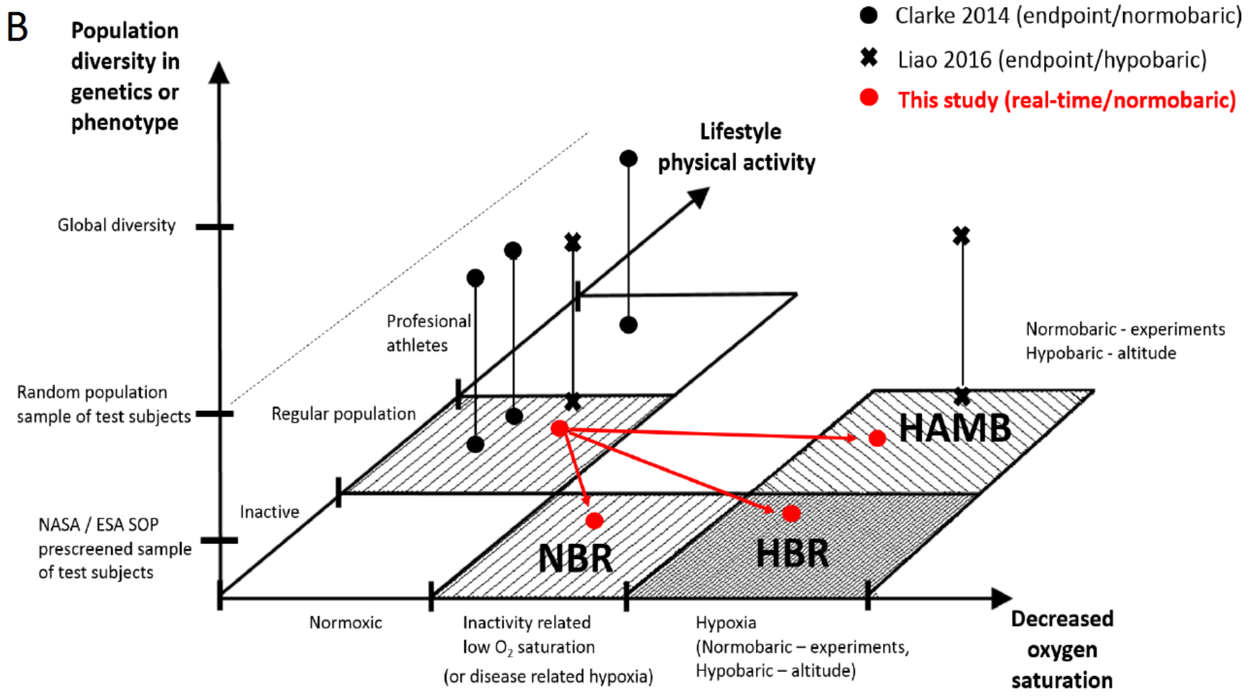

C

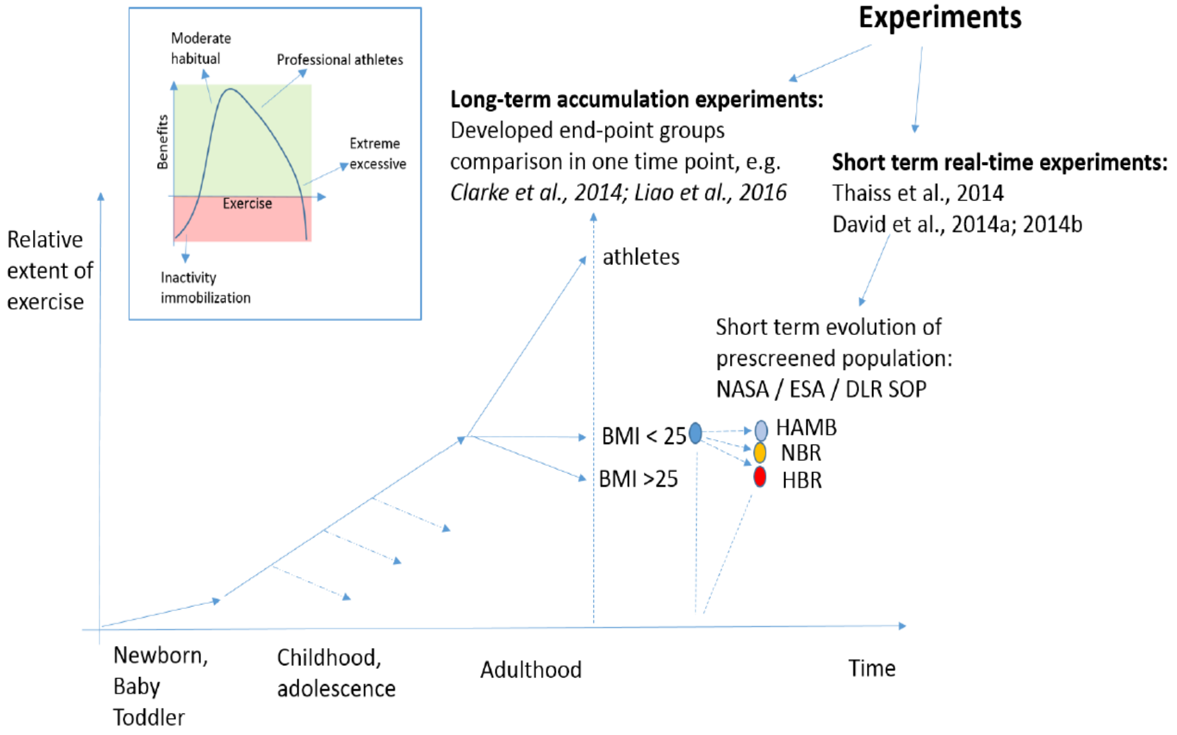
Experiments
Long-term accumulation experiments:
Developed end-point groups comparison in one time point, e.g. Clarke et al., 2014; Liao et al., 2016
Short term real-time experiments:
Thaiss et al., 2014
David et al., 2014a; 2014b
Moderate habitual
Professional athletes
Benefits
Extreme excessive
Exercise
Inactivity immobilization
Relative extent of exercise
athletes
Short term evolution of prescreened population:
NASA / ESA / DLR SOP
BMI < 25
BMI >25
HAMB
NBR
HBR
Newborn, Baby Toddler
Childhood, adolescence
Adulthood
Time

D

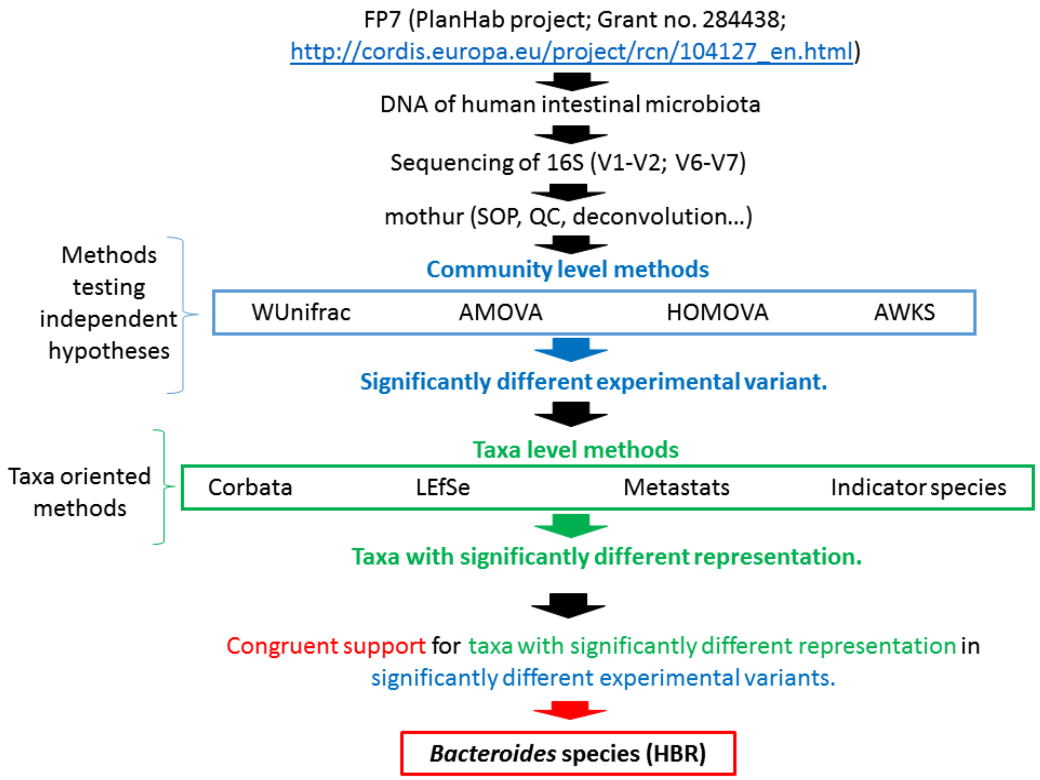
FP7 (PlanHab project; Grant no. 284438; http://cordis.europa.eu/project/rcn/104127_en.html)
DNA of human intestinal microbiota
Sequencing of 16S (V1-V2; V6-V7)
mothur (SOP, QC, deconvolution…)
Community level methods
Methods testing independent hypotheses
WUnifrac
AMOVA
HOMOVA
AWKS
Significantly different experimental variant.
Taxa level methods
Taxa oriented methods
Corbata
LEfSe
Metastats
Indicator species
Taxa with significantly different representation.
Congruent support for taxa with significantly different representation in significantly different experimental variants.
Bacteroides species (HBR)

REFERENCES

1 Clarke SF, Murphy EF, O'Sullivan O, *et al.* Exercise and associated dietary extremes impact on gut microbial diversity. *Gut* 2014;**63**:1913–20. doi:10.1136/gutjnl-2013-306541

2 Debevec T, Bali TC, Simpson EJ, *et al.* Separate and combined effects of 21-day bed rest and hypoxic confinement on body composition. *Eur J Appl Physiol* 2014;**114**:2411–25. doi:10.1007/s00421-014-2963-1

3 Liao WT, Liu B, Chen J, *et al.* Metabolite Modulation in Human Plasma in the Early Phase of Acclimatization to Hypobaric Hypoxia. *Sci Rep* 2016;**6**:22589. doi:10.1038/srep22589

4 Sket R, Treichel N, Debevec T, *et al.* Hypoxia and inactivity related physiological changes (constipation, inflammation) are not reflected at the level of gut metabolites and butyrate producing microbial community: The PlanHab study. *Front Physiol* 2017;**8**:250. doi:10.3389/fphys.2017.00250

5 Bermon S, Petriz B, Kajeniene A, *et al.* The microbiota: An exercise immunology perspective. *Exerc Immunol Rev* 2015;**21**:70–9.

6 Cronin O, Molloy MG, Shanahan F. Exercise, fitness, and the gut. *Curr Opin Gastroenterol* 2016;**32**:67–73. doi:10.1097/MOG.0000000000000240

7 Egan B, Zierath JR. Exercise metabolism and the molecular regulation of skeletal muscle adaptation. *Cell Metab* 2013;**17**:162–84. doi:10.1016/j.cmet.2012.12.012

8 Ringseis R, Eder K, Mooren FC, *et al.* Metabolic signals and innate immune activation in obesity and exercise. *Exerc Immunol Rev* 2015;**21**:58–68.
