# Supplementary material for: Hypoxia and inactivity related physiological changes precede or take place in absence of significant rearrangements in bacterial community structure: The PlanHab randomized trial pilot study
Source: PLoS One. 2017 Dec 6;12(12):e0188556. doi: 10.1371/journal.pone.0188556 (PMC5718606; doi:10.1371/journal.pone.0188556)
Supplement: S2 Fig — Taxa_S, Individuals, Dominance_D, Simpson_1-D, Shannon_H, Evenness_e^H/S, Brillouin, Menhinick, Margalef, Equitability_J, Fisher_α, Berger-Parker, Chao-1. (PDF) [file pone.0188556.s002.pdf]

S2 Figs. Ecological indices used to assess α-diversity microbiomes at the start-up and endpoints of the PlanHab experiment: Taxa_S, Individuals, Dominance_D, Simpson_1-D, Shannon_H, Evenness_e^H/S, Brillouin, Menhinick, Margalef, Equitability_J, Fisher_α, Berger-Parker, Chao-1.

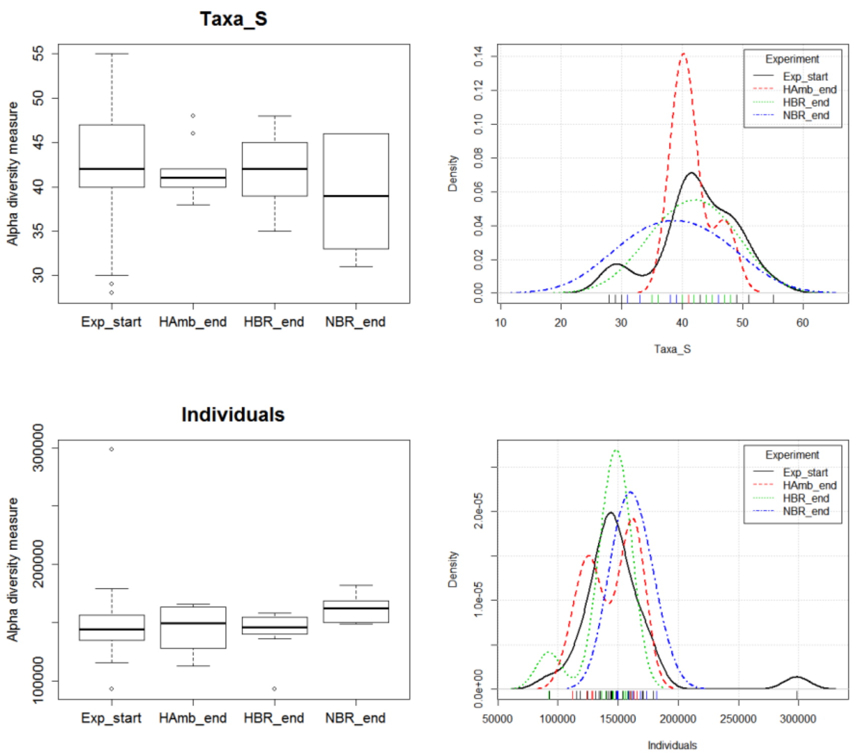

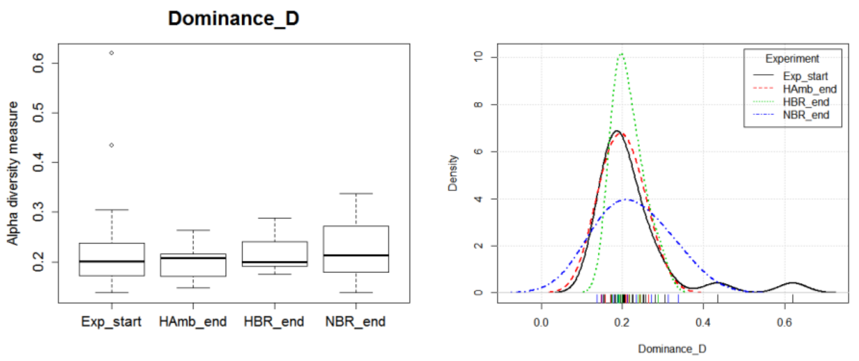
Dominance_D
Alpha diversity measure
Exp_start
HAmb_end
HBR_end
NBR_end
Density
Experiment
Dominance_D

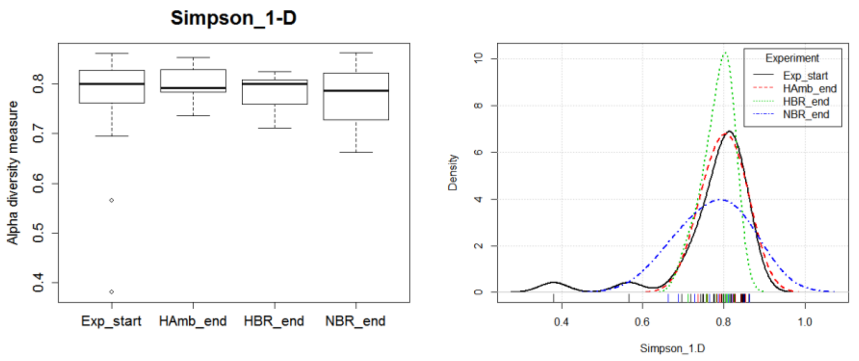
Simpson_1-D
Alpha diversity measure
Exp_start
HAmb_end
HBR_end
NBR_end
Density
Experiment
Simpson_1.D

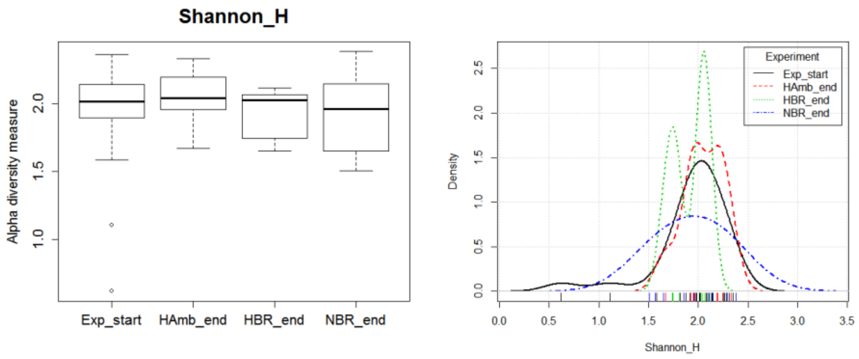
Shannon_H
Alpha diversity measure
Exp_start
HAmb_end
HBR_end
NBR_end
Density
Experiment
Shannon_H

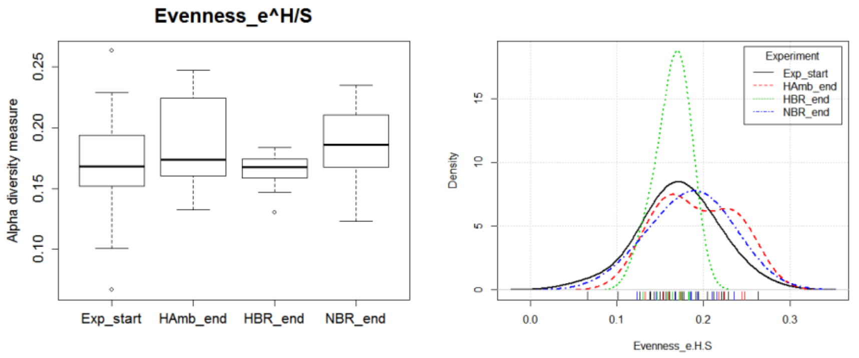
Evenness_e^H/S
Alpha diversity measure
Exp_start
HAmb_end
HBR_end
NBR_end
Density
Experiment
Evenness_e.H.S

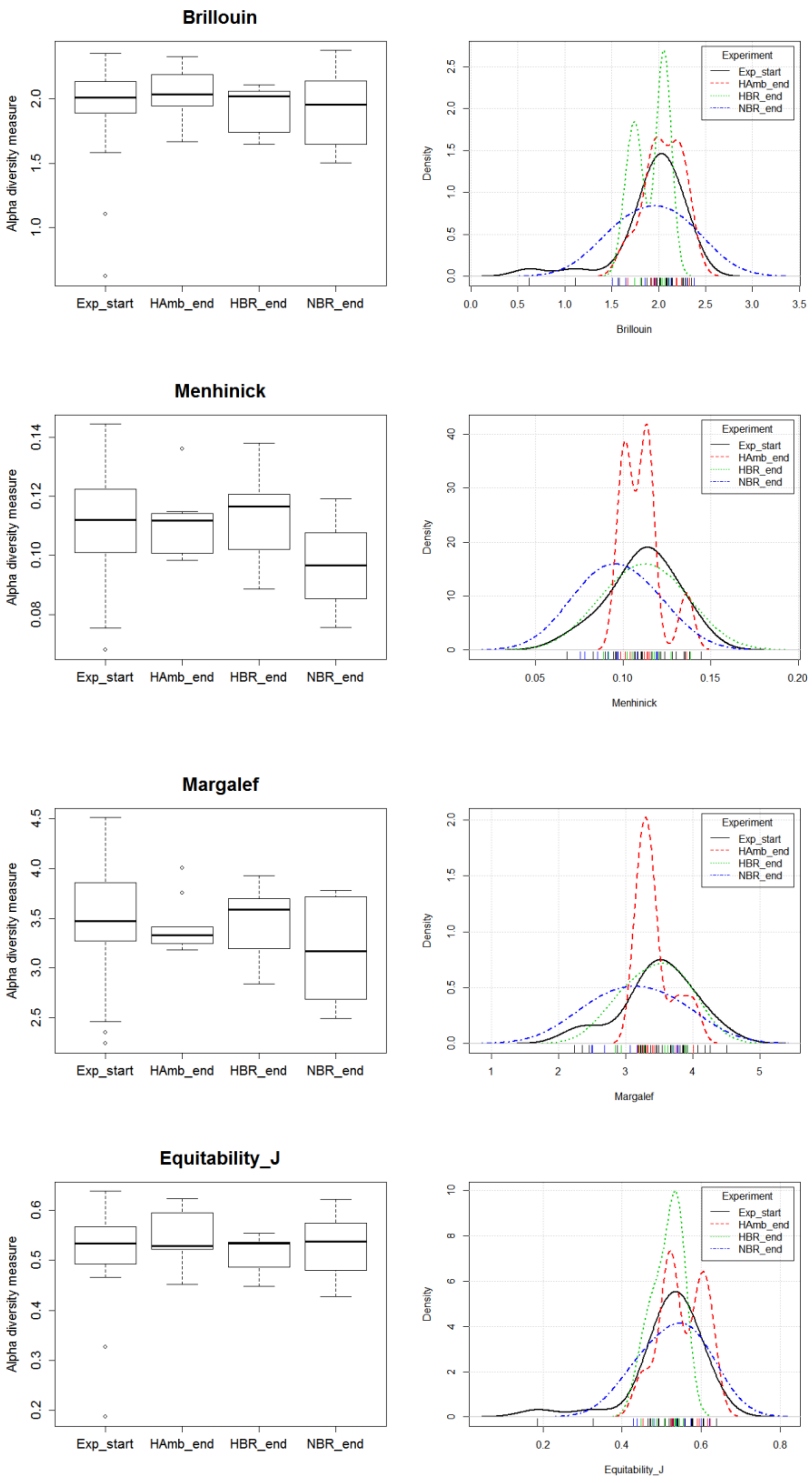
Brillouin
Alpha diversity measure
Exp_start
HAmb_end
HBR_end
NBR_end
Density
Experiment
Exp_start
HAmb_end
HBR_end
NBR_end
Brillouin
Menhinick
Alpha diversity measure
Exp_start
HAmb_end
HBR_end
NBR_end
Density
Experiment
Exp_start
HAmb_end
HBR_end
NBR_end
Menhinick
Margalef
Alpha diversity measure
Exp_start
HAmb_end
HBR_end
NBR_end
Density
Experiment
Exp_start
HAmb_end
HBR_end
NBR_end
Margalef
Equitability_J
Alpha diversity measure
Exp_start
HAmb_end
HBR_end
NBR_end
Density
Experiment
Exp_start
HAmb_end
HBR_end
NBR_end
Equitability_J

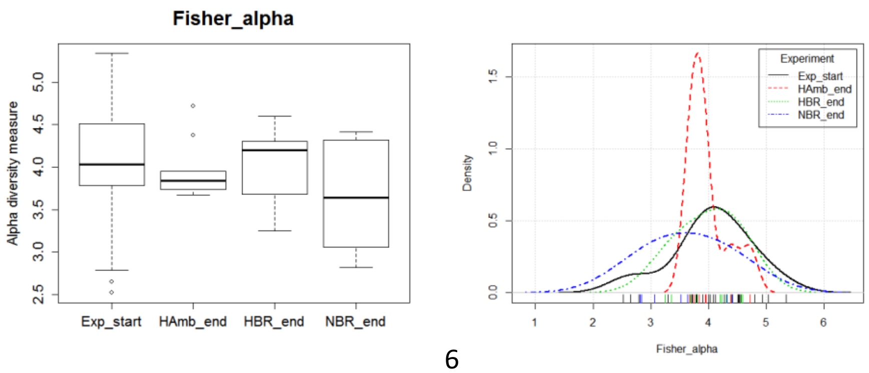
Fisher_alpha
Alpha diversity measure
Exp_start
HAmb_end
HBR_end
NBR_end
Density
Experiment
Fisher_alpha

6

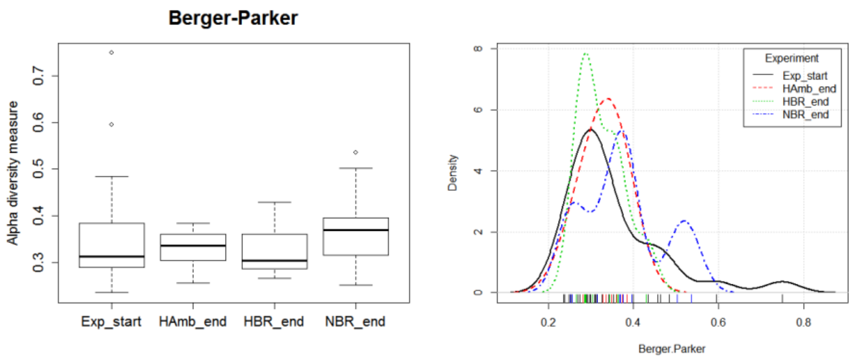
Berger-Parker
Alpha diversity measure
Exp_start
HAmb_end
HBR_end
NBR_end
Density
Experiment
Berger.Parker

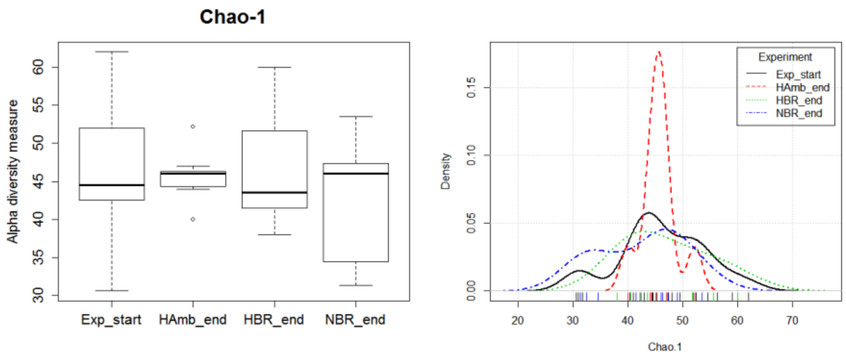
Chao-1
Alpha diversity measure
Exp_start
HAmb_end
HBR_end
NBR_end
Density
Experiment
Chao.1
