# Supplementary material for: Hypoxia and inactivity related physiological changes precede or take place in absence of significant rearrangements in bacterial community structure: The PlanHab randomized trial pilot study
Source: PLoS One. 2017 Dec 6;12(12):e0188556. doi: 10.1371/journal.pone.0188556 (PMC5718606; doi:10.1371/journal.pone.0188556)
Supplement: S3 Fig — The overall significant increase in various strains of Bacteroides at the end of PlanHab experiment in HBR is shown (p < 0.05). (PDF) [file pone.0188556.s003.pdf]

S3 Fig. Strain level deconvolution of the genus *Bacteroides* sequences found in NBR, HBR and HAmb variants at the end of the PlanHab experiments. The overall significant increase in various strains of *Bacteroides* at the end of PlanHab experiment in HBR is shown ($p < 0.05$) (Continues below).

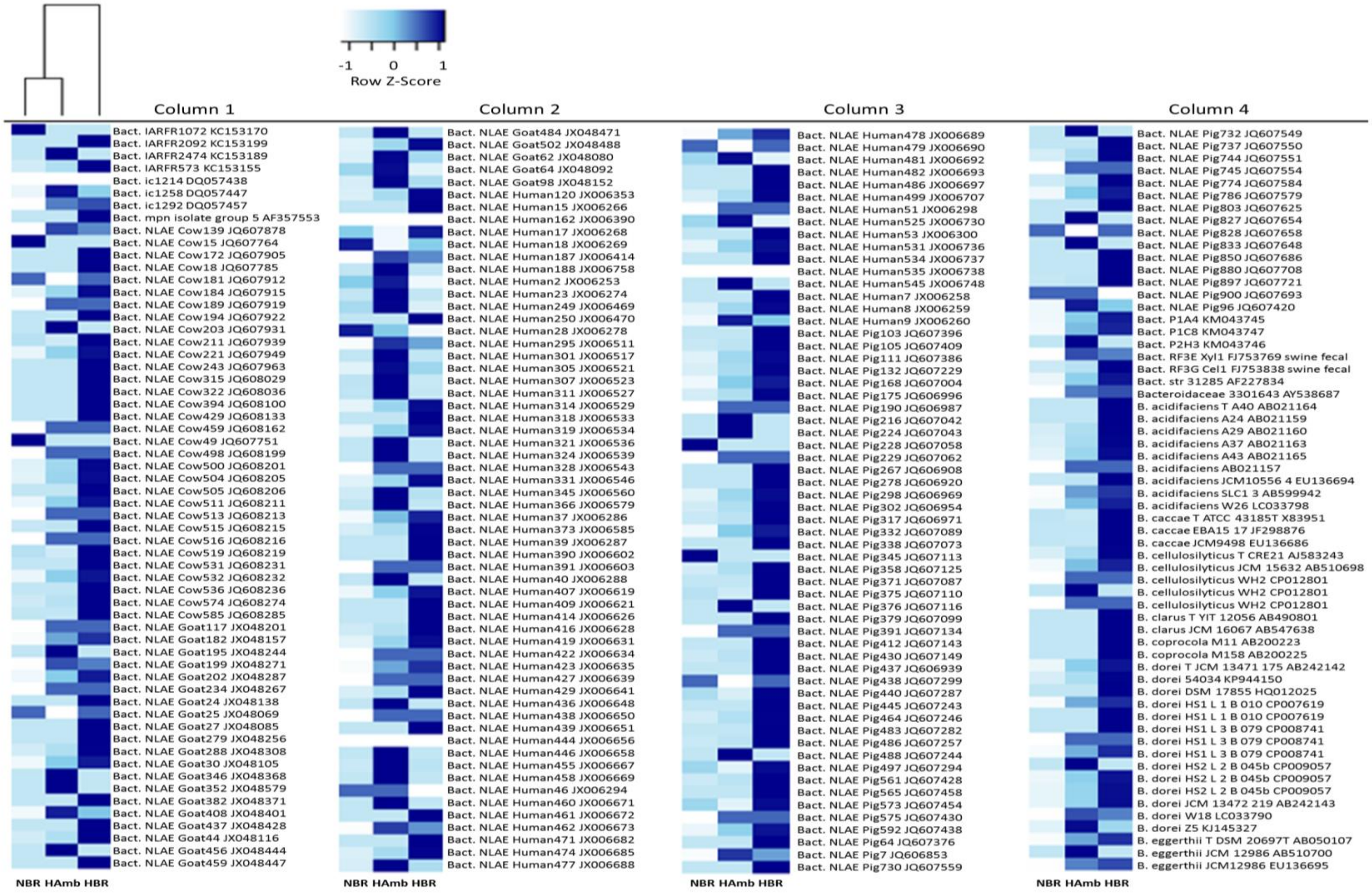

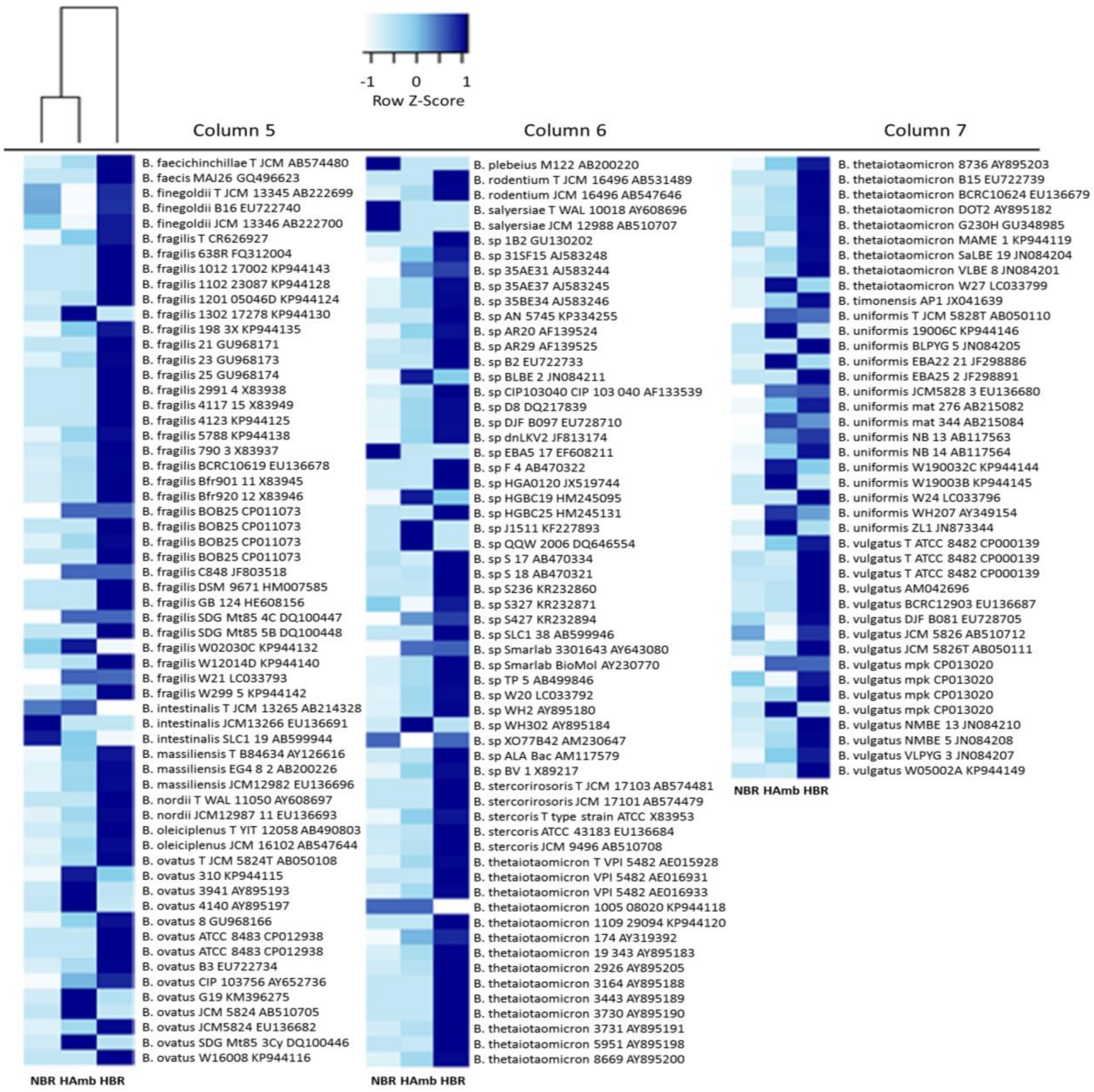
-1 0 1
Row Z-Score
Column 5
Column 6
Column 7
B. faecichinchillae T JCM AB574480
B. faecis MAJ26 GQ496623
B. finegoldii T JCM 13345 AB222699
B. finegoldii B16 EU722740
B. finegoldii JCM 13346 AB222700
B. fragilis T CR626927
B. fragilis 638R FQ312004
B. fragilis 1012 17002 KP944143
B. fragilis 1102 23087 KP944128
B. fragilis 1201 05046D KP944124
B. fragilis 1302 17278 KP944130
B. fragilis 198 3X KP944135
B. fragilis 21 GU968171
B. fragilis 23 GU968173
B. fragilis 25 GU968174
B. fragilis 2991 4 X83938
B. fragilis 4117 15 X83949
B. fragilis 4123 KP944125
B. fragilis 5788 KP944138
B. fragilis 790 3 X83937
B. fragilis BCRC10619 EU136678
B. fragilis Bfr901 11 X83945
B. fragilis Bfr920 12 X83946
B. fragilis BOB25 CP011073
B. fragilis BOB25 CP011073
B. fragilis BOB25 CP011073
B. fragilis BOB25 CP011073
B. fragilis C848 JF803518
B. fragilis DSM 9671 HM007585
B. fragilis GB 124 HE608156
B. fragilis SDG Mt85 4C DQ100447
B. fragilis SDG Mt85 5B DQ100448
B. fragilis W02030C KP944132
B. fragilis W12014D KP944140
B. fragilis W21 LC033793
B. fragilis W299 5 KP944142
B. intestinalis T JCM 13265 AB214328
B. intestinalis JCM13266 EU136691
B. intestinalis SLC1 19 AB599944
B. massiliensis T B84634 AY126616
B. massiliensis EG4 8 2 AB200226
B. massiliensis JCM12982 EU136696
B. nordii T WAL 11050 AY608697
B. nordii JCM12987 11 EU136693
B. oleiciplenus T YIT 12058 AB490803
B. oleiciplenus JCM 16102 AB547644
B. ovatus T JCM 5824T AB050108
B. ovatus 310 KP944115
B. ovatus 3941 AY895193
B. ovatus 4140 AY895197
B. ovatus 8 GU968166
B. ovatus ATCC 8483 CP012938
B. ovatus ATCC 8483 CP012938
B. ovatus B3 EU722734
B. ovatus CIP 103756 AY652736
B. ovatus G19 KM396275
B. ovatus JCM 5824 AB510705
B. ovatus JCM5824 EU136682
B. ovatus SDG Mt85 3Cy DQ100446
B. ovatus W16008 KP944116
NBR HAmb HBR
B. plebeius M122 AB200220
B. rodentium T JCM 16496 AB531489
B. rodentium JCM 16496 AB547646
B. salyersiae T WAL 10018 AY608696
B. salyersiae JCM 12988 AB510707
B. sp 1B2 GU130202
B. sp 31SF15 AJ583248
B. sp 35AE31 AJ583244
B. sp 35AE37 AJ583245
B. sp 35BE34 AJ583246
B. sp AN 5745 KP334255
B. sp AR20 AF139524
B. sp AR29 AF139525
B. sp B2 EU722733
B. sp BLBE 2 JN084211
B. sp CIP103040 CIP 103 040 AF133539
B. sp D8 DQ217839
B. sp DJF B097 EU728710
B. sp dnLKV2 JF813174
B. sp EBA5 17 EF608211
B. sp F 4 AB470322
B. sp HGA0120 JX519744
B. sp HGBC19 HM245095
B. sp HGBC25 HM245131
B. sp J1511 KF227893
B. sp QQW 2006 DQ646554
B. sp S 17 AB470334
B. sp S 18 AB470321
B. sp S236 KR232860
B. sp S327 KR232871
B. sp S427 KR232894
B. sp SLC1 38 AB599946
B. sp Smarlab 3301643 AY643080
B. sp Smarlab BioMol AY230770
B. sp TP 5 AB499846
B. sp W20 LC033792
B. sp WH2 AY895180
B. sp WH302 AY895184
B. sp XO77B42 AM230647
B. sp ALA Bac AM117579
B. sp BV 1 X89217
B. stercorirosoris T JCM 17103 AB574481
B. stercorirosoris JCM 17101 AB574479
B. stercoris T type strain ATCC X83953
B. stercoris ATCC 43183 EU136684
B. stercoris JCM 9496 AB510708
B. thetaiotaomicron T VPI 5482 AE015928
B. thetaiotaomicron VPI 5482 AE016931
B. thetaiotaomicron VPI 5482 AE016933
B. thetaiotaomicron 1005 08020 KP944118
B. thetaiotaomicron 1109 29094 KP944120
B. thetaiotaomicron 174 AY319392
B. thetaiotaomicron 19 343 AY895183
B. thetaiotaomicron 2926 AY895205
B. thetaiotaomicron 3164 AY895188
B. thetaiotaomicron 3443 AY895189
B. thetaiotaomicron 3730 AY895190
B. thetaiotaomicron 3731 AY895191
B. thetaiotaomicron 5951 AY895198
B. thetaiotaomicron 8669 AY895200
NBR HAmb HBR
B. thetaiotaomicron 8736 AY895203
B. thetaiotaomicron B15 EU722739
B. thetaiotaomicron BCRC10624 EU136679
B. thetaiotaomicron DOT2 AY895182
B. thetaiotaomicron G230H GU348985
B. thetaiotaomicron MAME 1 KP944119
B. thetaiotaomicron SaLBE 19 JN084204
B. thetaiotaomicron VLBE 8 JN084201
B. thetaiotaomicron W27 LC033799
B. timonensis AP1 JX041639
B. uniformis T JCM 5828T AB050110
B. uniformis 19006C KP944146
B. uniformis BLPYG 5 JN084205
B. uniformis EBA22 21 JF298886
B. uniformis EBA25 2 JF298891
B. uniformis JCM5828 3 EU136680
B. uniformis mat 276 AB215082
B. uniformis mat 344 AB215084
B. uniformis NB 13 AB117563
B. uniformis NB 14 AB117564
B. uniformis W190032C KP944144
B. uniformis W19003B KP944145
B. uniformis W24 LC033796
B. uniformis WH207 AY349154
B. uniformis ZL1 JN873344
B. vulgatus T ATCC 8482 CP000139
B. vulgatus T ATCC 8482 CP000139
B. vulgatus T ATCC 8482 CP000139
B. vulgatus AM042696
B. vulgatus BCRC12903 EU136687
B. vulgatus DJF B081 EU728705
B. vulgatus JCM 5826 AB510712
B. vulgatus JCM 5826T AB050111
B. vulgatus mpk CP013020
B. vulgatus mpk CP013020
B. vulgatus mpk CP013020
B. vulgatus mpk CP013020
B. vulgatus NMBE 13 JN084210
B. vulgatus NMBE 5 JN084208
B. vulgatus VLPYG 3 JN084207
B. vulgatus W05002A KP944149
NBR HAmb HBR
