# Supplementary material for: Hypoxia and inactivity related physiological changes precede or take place in absence of significant rearrangements in bacterial community structure: The PlanHab randomized trial pilot study
Source: PLoS One. 2017 Dec 6;12(12):e0188556. doi: 10.1371/journal.pone.0188556 (PMC5718606; doi:10.1371/journal.pone.0188556)

S4 Fig. A schematic presentation of interactive and dose dependent responses to physical inactivity and overeating over the course of human evolution.

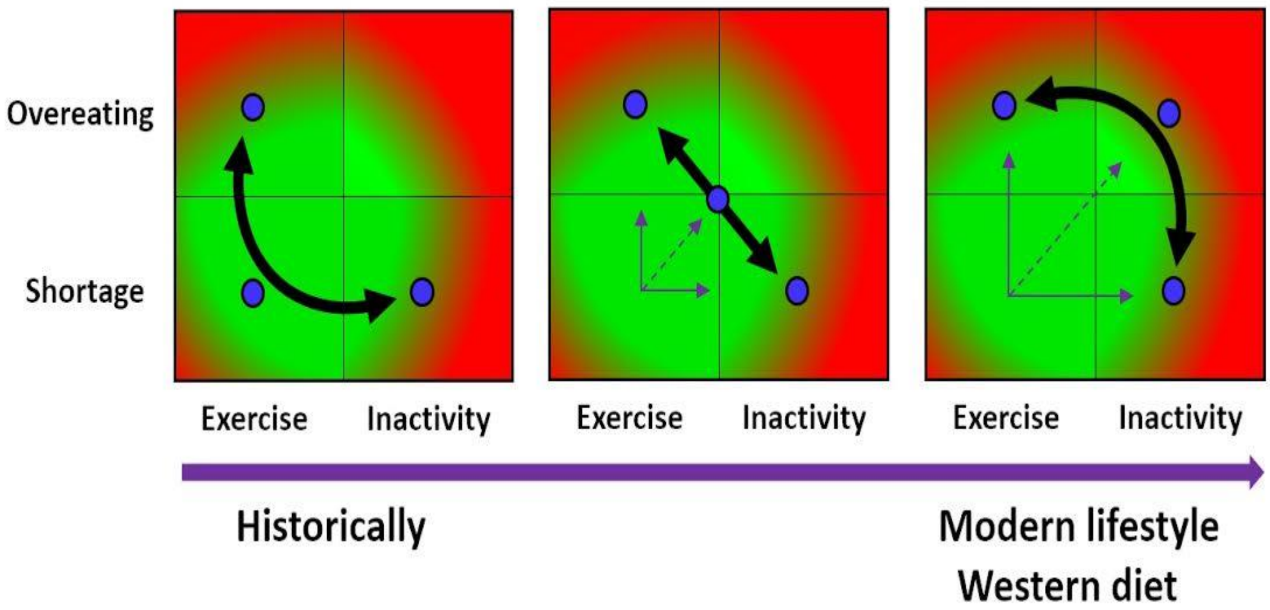

Supplement: S4 Fig — (PDF) [file pone.0188556.s004.pdf]
