# Supplementary material for: Hypoxia and inactivity related physiological changes precede or take place in absence of significant rearrangements in bacterial community structure: The PlanHab randomized trial pilot study
Source: PLoS One. 2017 Dec 6;12(12):e0188556. doi: 10.1371/journal.pone.0188556 (PMC5718606; doi:10.1371/journal.pone.0188556)
Supplement: S1 Table — (PDF) [file pone.0188556.s005.pdf]

S1 Table. Baseline demographic and clinical characteristics for each experiment group (mean ± SD) in PlanHab experiment.

Experiment	Sex	Nationality	Age (years)		Height (m)		Weight (Kg)		BMI (kg/m2)	
			Mean	SD	Mean	SD	Mean	SD	Mean	SD
NBR	Male	Caucasian	32.7	6.2	1.79	0.01	69.9	3.7	21.8	1.2
HAmb	Male	Caucasian	26.7	0.5	1.85	0.06	82.8	8.7	24.3	2.4
HBR	Male	Caucasian	23.0	2.9	1.77	0.01	72.7	11.8	23.2	3.5
