# Supplementary material for: Hypoxia and inactivity related physiological changes precede or take place in absence of significant rearrangements in bacterial community structure: The PlanHab randomized trial pilot study
Source: PLoS One. 2017 Dec 6;12(12):e0188556. doi: 10.1371/journal.pone.0188556 (PMC5718606; doi:10.1371/journal.pone.0188556)
Supplement: S2 Table — The three data matrices containing (i) experimental, (ii) diet, and (iii) metabolite and immune status related parameters (n = 231) were used in variation partitioning of bacterial microbial community datasets in this study. (PDF) [file pone.0188556.s006.pdf]

S2 Table. Overview of new in-house PlanHab database. The three data matrices containing (i) experimental, (ii) diet, and (iii) metabolite and immune status related parameters (n=231) were used in variation partitioning of bacterial microbial community datasets in this study.

**Experiment**	**Diet**				**Environment: metabolite and immunological parameters**
Participant (n=9)	aaeb - Branched-chain amino acids (g)	f18_3n3 - "no name" (g)	his - Histidine (g)	trp - Tryptophan (g)	pH
Hypoxia (n=2)	aat - Total amino acids (g)	f18_3n6 - "no name" (g)	id - Identifier	tyr - Tyrosine (g)	reducing sugars - Colorimetric detection of reducing sugars content
Inactivity (n=2)	ala - Alanine (g)	f18_4n3 - "no name" (g)	ile - Isoleucine (g)	val - Valine (g)	%water.feces - Water content in feces (%)
Sample (n=54)	alc - Alchocol (g)	f20_2n6 - "no name" (g)	k - Potassium (mg)	vita - Vitamin A (µg)	Acetic - Acetic acid (g/L)
Time in experiment(n=6)	arg - Arginine (g)	f20_3n6 - "no name" (g)	kcal - Kilocalories	vitb12 - Vitamin B12 (µg)	Propionic - Propionic acid (g/L)
Experimental variant (n=3)	asp - Aspartate (g)	f20_4n6 - "no name" (g)	lacs - Lactose (g)	vitb6 - Vitamin B6 (mg)	isoBut - iso-Butiric acid (g/L)
Age (year)	biot - Vitamin B7 (µg)	f20_5n3 - "no name" (g)	leu - Leucine (g)	vitc - Vitamin C (mg)	nBut - n-Butiric acid (g/L)
Height (cm)	ca - Calcium (mg)	f22_4n6 - "no name" (g)	lys - Lysine (g)	vitd - Vitamin D (µg)	isoVal - iso-Valeric acid (g/L)
Body mass (kg)	carta - alpha-Carotene (µg)	f22_5n3 - "no name" (g)	mals - Maltose (g)	vite - Vitamin E (mg)	nVal - n-Valeric acid (g/L)
BMI - Body mass index	cartb - beta-Carotene(µg)	f22_6n3 - Docosahexaenoic fatty acid (g)	met - Methionine	vitk - Vitamin K(µg)	nCapric - n-Capric acid (g/L)
	cho - Total carbohydrates (g)	fams - Monounsaturated fatty acids (g)	mg - Magnesium (mg)	water - Water (g)	totSCFA - Total short chain fatty acids (g/L)
	chorl - Cholesterol (mg)	fapu - Total polyunsaturated fatty acids(g)	mn - Manganese (µg)	zn - Zinc (mg)	TSOC - Total soluble organic carbon per gram of dry matter in feces
	chot - Total carbohydrates (g)	fapun3 - Total omega-3 fatty acids (g)	mo - Molybdenum (µg)		BA - Bile acids (fold)
	chot_p - Total carbohydrates (%)	fapun6 - Total omega-6 fatty acids (g)	na - Sodium (mg)		zonulin - Zonulin (fold)
	cld - Chlorine (mg)	fasat - Saturated fatty acids (g)	nacl - Sodium chloride (g)		a1aT - Alpha 1 anti-trypsin (fold)
	color - Color	fat - Total fat (g)	nia - Vitamin B3 (µg)		EDN - Eosinophile derived neurotooxin (fold)
	cr - Chromium (µg)	fat_p - Total fat (%)	p - Phosphorus (mg)		BSS - Bristol stool scale
	cu - Copper (µg)	fd - Fluoride (µg)	pantac - Vitamin B5 (mg)		retention time - Time between particular defecations (days)
	cyste - Cysteine (g)	fe - Iron (mg)	phe - Phenylalanine (mg)		defecation frequency - Number of defecations during experiments
	edible - "no name"	fibc - Crude fiber (g)	pro - Proline (g)		Sr - Ratio of absorbation slopes between 275-295 nm slope and 350-400 nm slope
	enera - Energy (kcal)	fiber.energy.kcal - Total fiber energy yield (kcal)	prot - Total protein (g)		E2.E3 - Ratio between absorption coeficitents at 250 nm and at 365 nm
	f14_0 - Myristic fatty acid (g)	fibins - Insoluble fiber (g)	prot_p - Total protein (%)		a255 - Absorption coeficient at 255 nm
	f16_0 - Palmitic fatty acid (g)	fibsol - Soluble fiber (g)	protan - Protein of animal origin (g)		a.300 - Absorption coeficient at 300 nm
	f18_0 - Stearic fatty acid (g)	fibt - Total fiber (g)	protpl - Protein of plant origin (g)		Total.a.250.450 - Integrated absorbance between 250 nm and 450 nm
	f18_1cn9 - Oleic fatty acid (g)	fol - Folic acid (µg)	ribf - Vitamin B2 (mg)		S.300.700 - Spectral slope of absorbance from 300 nm to 700 nm
	f18_2 - Linoleic fatty acid(g)	frus - Fructose (g)	se - Selenium (µg)		**totalSterols (area under curve)**
	f18_2cn6 - "no name" (g)	fsugar - Available sugars (g)	ser - Serine (g)		**totalPolyphenols (area under curve)**
	f18_2con - "no name" (g)	gals - Galactose (g)	sq - "no name"		**profileSterols (n=12) and Shannon diversity index**
	f18_2ct - "no name" (g)	gi - GI - glycemic index	ssugar - Total simple sugars (g)		**profilePolyphenols (n=41) and Shannon diversity index**
	f18_2iso - "no name" (g)	gl - GL - glycemic load	starch - Starch (g)		**Conductivity (mS/cm)**
	f18_2tn - "no name" (g)	gln - Glutamine (g)	sucs - Sucrose (g)		**SUVA245 - specific ultraviolet absorption (fold)**
	f18_3 - Linolenic fatty acid (g)	glu - Glutamate (g)	sugar - Total sugars (g)		**SViA420 - specific visual absorption (fold)**
	f18_3cn3 - "no name" (g)	glus - Glucose (g)	thia - Vitamin B1 (mg)		**TDLP9 - sum of nine p-hydroxy, vanillyl, and syringyl phenols (fold)**
	f18_3cn6 - "no name" (g)	gly - Glycine (g)	thr - Threonine (g)		**Indole level index (fold)**
