# Supplementary material for: Hypoxia and inactivity related physiological changes precede or take place in absence of significant rearrangements in bacterial community structure: The PlanHab randomized trial pilot study
Source: PLoS One. 2017 Dec 6;12(12):e0188556. doi: 10.1371/journal.pone.0188556 (PMC5718606; doi:10.1371/journal.pone.0188556)
Supplement: S3 Table — Identification of Bacteroides species at the end of PlanHab experiment with their mucin degrading and inflammogenic characteristics of medical relevance reconstructed from published literature. The k-mer search strategy (RDPTools; n = 7) with Sab score>0.87 to type and cultivated strains with reported relevance for dysbiotic medical conditions and described metabolic characteristics was used in this analysis.[9–17] (PDF) [file pone.0188556.s007.pdf]

S3 Table. Characteristics of identified *Bacteroides* species. Identification of *Bacteroides* species at the end of PlanHab experiment with their mucin degrading and inflammogenic characteristics of medical relevance reconstructed from published literature. The k-mer search strategy (RDPTools; n=7) with Sab score>0.87 to type and cultivated strains with reported relevance for dysbiotic medical conditions and described metabolic characteristics was used in this analysis.[1–9]

***Bacteroides* species**	**[1]Degradation of mucin**	**[2]α-Fucosidase activity**	**[3]Indole production**	**[4]Aesculin hydrolysis**	**[5]Associated with the following clinical manifestations**
B. acidifaciens	+	+	-	+	inflammatory bowel disease
B. caccae	+	+	-	+	inflammatory bowel disease
B. dorei	+	+	-	-	type 1 diabetes
<u>*B. fragilis*</u>	+	+	-	+	inflammatory bowel disease
<u>*B. ovatus*</u>	+	+	+	+	type 1 diabetes, inflammation
<u>*B. thetaiotamicron*</u>	+	+	+	+	enteric infections, inflammation
<u>*B. vulgatus*</u>	-	+	-	+	ulcerative colitis

1 Bacteroides derive nutrients from either diet or host derived or commensal derived polymers. Overutilization of host derived polymers (e.g. during constipation) results in thinning of mucus layer and modified barrier functioning.[8, 10]

2 De-glycosylation of mucin components leads to degradation mucus properties and degradation of collocated immune compounds (IgA). Free fucose is a danger signal to human intestinal epithelial cells and is directly utilized in fermentations by facultatively anaerobic enteropathogenic *E.coli* and other bacteria, affecting the mucosal physicochemical characteristics.[11]

3 Indole is a microbe-generated signal substance that has positive effects on its host as well as the microbiome. Indole functions as a ‘quorum-sensing’ signal that regulates the virulence and biofilm formation of EHEC, *Pseudomonas* and other commensal bacteria. Indole strengthens the barrier function of the mucous membrane by repairing ‘tight junctions’.[12, 13]

4 Aesculin hydrolysis *in-vivo* generates secondary derivatives from the family of coumarins that exert potent positive pharmacological effects on the host.[14]

5 The same commensal *Bacteroides* species observed in numerous healthy fecal samples undergo significant metabolic changes associated with the clinical manifestations under specific environmental conditions.

The <u>**underlined species**</u> were recently identified as those exhibiting extensive genomic variants with markedly different metabolic capacities in patients.[15]

REFERENCES

1 Bakir MA, Sakamoto M, Kitahara M, *et al.* Bacteroides dorei sp. nov., isolated from human faeces. *Int J Syst Evol Microbiol* 2006;**56**:1639–1643. doi:10.1099/ijs.0.64257-0

2 Bloom SM, Bijanki VN, Nava GM, *et al.* Commensal Bacteroides species induce colitis in host-genotype-specific fashion in a mouse model of inflammatory bowel disease. *Int Rev Immunol* 2012;**31**:410–27. doi:10.1016/j.chom.2011.04.009.Commensal

3 Brook I. Bacteroides infections in children. *J Med Microbiol* 1995;**43**:92–8.

4 Davis-Richardson AG, Ardissone AN, Dias R, *et al.* Bacteroides dorei dominates gut microbiome prior to autoimmunity in Finnish children at high risk for type 1 diabetes. *Front Microbiol* 2014;**5**:1–11. doi:10.3389/fmicb.2014.00678

5 Jousimies-Somer HR, Summanen PH, Wexler H, *et al.* Bacteroides, Porphyromonas, Prevotella, Fusobacterium, and other anaerobic Gram-negative bacteria. In: Murray PR, Baron EJ, Jorgensen JH, *et al.*, eds. *Manual of Clinical Microbiology, 8th edn*. Washington, DC: : American Society for Microbiology. 2003. 880–901.

6 Miyamoto Y, Itoh K. Bacteroides acidifaciens sp. nov., isolated from the caecum of mice. *Int J Syst Evol Microbiol* 2000;**50**:145–8. doi:10.1099/00207713-50-1-145

7 Salyers AA, Vercellotti JR, West SEH, *et al.* Fermentation of mucin and plant polysaccharides by strains of Bacteroides from the human colon. *Appl Environ Microbiol* 1977;**33**:319–22.

8 Tailford LE, Crost EH, Kavanaugh D, *et al.* Mucin glycan foraging in the human gut microbiome. *Front Genet* 2015;**5**. doi:10.3389/fgene.2015.00081

9 Wexler HM. Bacteroides: The good, the bad, and the nitty-gritty. *Clin Microbiol Rev* 2007;**20**:593–621. doi:10.1128/CMR.00008-07

10 Jakobsson HE, Rodríguez-Piñeiro AM, Schütte A, *et al.* The composition of the gut microbiota shapes the colon mucus barrier. *EMBO Rep* 2015;**16**:164–77.

11 Chow WL, Lee YK. Free fucose is a danger signal to human intestinal epithelial cells. *Br J Nutr* 2008;**99**:449–54. doi:10.1017/S0007114507812062

12 Berstad A, Raa J, Valeur J. Indole - the scent of a healthy ‘inner soil’. *Microb Ecol Health Dis* 2015;**26**:1–5. doi:10.7554/eLife.06758

13 Shimada Y, Kinoshita M, Harada K, *et al.* Commensal bacteria-dependent indole production enhances epithelial barrier function in the colon. *PLoS One* 2013;**8**:1–10. doi:10.1371/journal.pone.0080604

14 Ding W-J, Deng Y, Feng H, *et al.* Biotransformation of aesculin by human gut bacteria and identification of its metabolites in rat urine. *World J Gastroenterol* 2009;**15**:1518–23. doi:10.3748/wjg.15.1518

15 Vineis JH, Ringus DL, Morrison HG, *et al.* Patient-specific Bacteroides genome variants in pouchitis. *MBio* 2016;**7**:1–11. doi:10.1128/mBio.01713-16.
