# Supplementary material for: Hypoxia and inactivity related physiological changes precede or take place in absence of significant rearrangements in bacterial community structure: The PlanHab randomized trial pilot study
Source: PLoS One. 2017 Dec 6;12(12):e0188556. doi: 10.1371/journal.pone.0188556 (PMC5718606; doi:10.1371/journal.pone.0188556)
Supplement: S4 Table — The data were retrieved from the newly established in-house PlanHab database and used in variation partitioning. Please see Materials and methods next to S2 Table for additional information. A1AT–Alpha1 antitrypsin; TSOC–total soluble organic carbon, EDN–eosinophil derived neurotoxin; BSS–Bristol stool scale; polyphenol a1, b1, a3 –polyphenol peaks with currently unknown chemical structure. (PDF) [file pone.0188556.s008.pdf]

S4 Table. Variables significantly associated with the distribution of bacterial community structure in PlanHab experiment at the level of 97 % OTUs and genus. The data were retrieved from the newly established in-house PlanHab database and used in variation partitioning. Please see Materials and methods next to Table S2 for additional information. A1AT – Alpha1 antitrypsin; TSOC – total soluble organic carbon, EDN – eosinophil derived neurotoxin; BSS – Bristol stool scale; polyphenol a1, b1, a3 – polyphenol peaks with currently unknown chemical structure.

97 % OTU

Environment	Experiment	Diet
**Variable**	**Variable**	**Variable**
iso-valeric acid	hypoxia	cholesterol
n-butyric acid	individual	histidine
n-capric acid	experimental variant	f18_3cn3 (alpha-linolenic acid)
A1AT	body traits (mass, height)	water
n-valeric acid		
TSOC		
EDN		
water content		
acetic acid		
bile acids		
reducing sugars		

Genus

Environment	Experiment	Diet
**Variable**	**Variable**	**Variable**
acetic acid	experimental variant	protein
n-butyric acid	hypoxia	fat
iso-valeric acid	time (day in experiment)	f18_2 (linoleic acid)
n-capric	individual	f18_3cn3 (alpha-linolenic acid)
A1AT	body traits (height, BMI)	serine
bile acids		sucrose
BSS		water
polyphenol a1		
polyphenol b1		
polyphenol a3		
