# Supplementary material for: Hypoxia and inactivity related physiological changes precede or take place in absence of significant rearrangements in bacterial community structure: The PlanHab randomized trial pilot study
Source: PLoS One. 2017 Dec 6;12(12):e0188556. doi: 10.1371/journal.pone.0188556 (PMC5718606; doi:10.1371/journal.pone.0188556)
Supplement: S1 Text — (PDF) [file pone.0188556.s009.pdf]

Planetary Habitat Simulation project Methodology

(PlanHab)

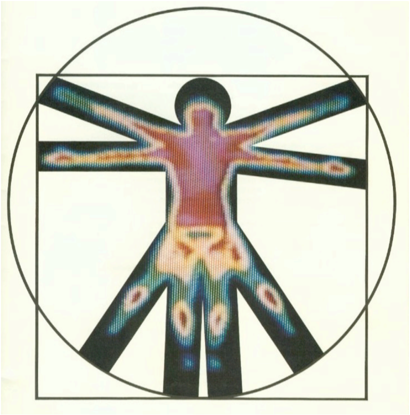

Coordinator and contact person:
Institut Jožef Stefan
Prof. dr. Igor B. Mekjavic (igor.mekjavic@ijs.si)

Abstract

The aim is to investigate the combined effects of hypoxia and sustained recumbency (bedrest), on human physiological systems. The partial pressure of oxygen in the environmental gas inside future planetary habitats will be lower than in atmospheric air. Prolonged exposure to low gravity will result in deconditioning of vital physiological systems, and may consequently constitute a threat to the health of the astronauts. However, it is unknown how prolonged exposure to both reduced gravity and hypoxia will affect health. The new knowledge has also implications for society in general, since chronic hypoxia and bedrest constitutes a model of the basic conditions experienced by patients suffering from respiratory insufficiency restricting them to a physically inactive life style. The challenge of the project lies in the complexity of the experimental interventions where healthy humans are confined to a hypoxic environment during prolonged bedrest. A series of studies will be conducted at the Planica hypoxia facility capable of housing 20 subjects at any simulated altitude. Subjects will remain in horizontal position (bedrest) or be ambulatory, but confined to the facility (ambulation) for 21 days/trial. Each subject will participate in three trials: hypoxic bedrest (simulated altitude 4000m), normoxic bedrest, and hypoxic ambulation. The effects will be investigated in experiments concerning metabolic, cardiorespiratory, musculoskeletal, haematological, immunological and thermoregulatory functions. In addition to the specific objectives, the study will be explorative in the sense that it will collect a broad spectrum of basic data corresponding to that obtained when 21-day bedrest experiments are conducted by ESA/NASA (bedrest core data). Thus, data from the experiments can readily be compared with core data from previous bedrest studies, and hence the added effects of hypoxia should be evident.

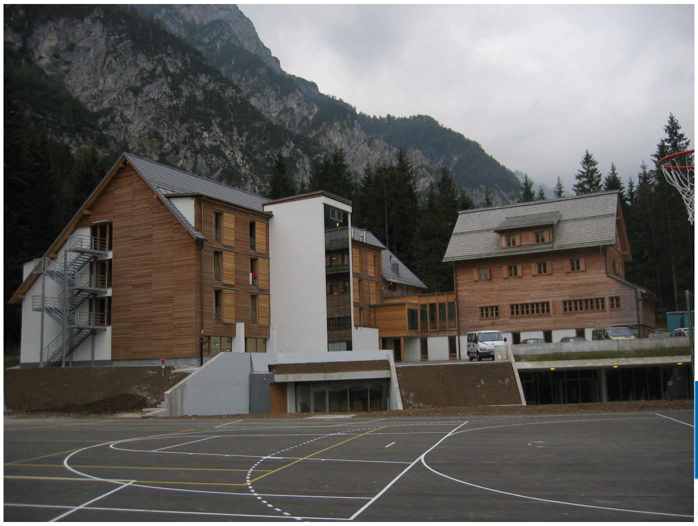

Evropski Sklad za Regionalni Razvoj

Projekt je delno financirala Evropska unija.

Figure 1: *The Planica Facility (Olympic Sport Centre Planica), built with EU Regional Development Funds, is the site of hypoxic bedrest and confinement studies. The hypoxic facility (building at left) is capable of simulating altitudes up to 5400 m.*

1.1 Concept and objectives

The aim of the proposed study is to investigate the combined effects of hypoxia and sustained recumbency (bedrest), on human physiological systems. Sustained bedrest is commonly used to simulate certain biological effects of reduced gravity. It is anticipated that the partial pressure of oxygen in the environmental gas inside future planetary habitats will be lower than atmospheric air (i.e. the astronauts will breathe a hypoxic gas mixture). Prolonged exposure to low gravity will result in deconditioning of several vital physiological systems, and may consequently constitute a threat to the health of the astronaut. However, it is presently unknown how prolonged exposure to both reduced gravity and hypoxia will affect the health of the astronauts.

Exploring effects of prolonged hypoxia and sustained bedrest is not only relevant from a space science perspective, but also has implications for society in general, since chronic hypoxia and bedrest constitutes a model of the basic conditions experienced by a common category of patients, namely those suffering from respiratory insufficiency restricting them to a physically inactive life style.

Study design

A series of bedrest and hypoxia studies involving human subjects will be conducted at a hypoxia facility (Olympic Sport Centre Planica, Slovenia) capable of housing up to 20 subjects simultaneously at any simulated altitude. During the course of the studies, subjects will remain in a horizontal position (bedrest) or be ambulatory, but confined to the facility (ambulation) for durations of 21 days. The studies will be conducted using a repeated-measures cross-over design (Fig. 2); each subject will participate in three trials: hypoxic bedrest, normoxic bedrest, and hypoxic ambulation. In between trials, the subjects will be allowed a wash-out period, which will be approx. 3 times the length of the bedrest or ambulation period. In the normoxia trial, subjects will be exposed to a normoxic environment (21 kPa oxygen) during the bedrest, whereas in the hypoxia trials, subjects will be exposed to a hypoxic environment for the duration of the bedrest or confinement. The level of hypoxia will be maintained at 12.5 kPa (corresponding to 4000 m above sea level), which is somewhat lower than the anticipated oxygen partial pressure in future planetary habitats.

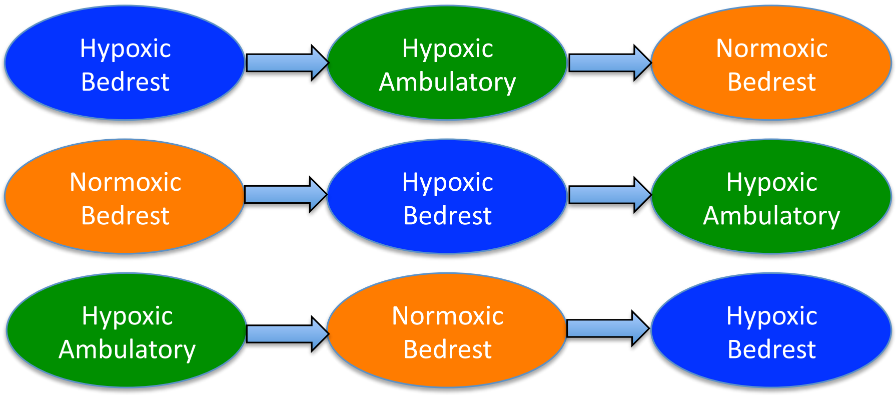

Figure 2: *Proposed study design: repeated-measures cross-over design (see text).*

The justification for lowering the oxygen fraction in the proposed experiments to the extent that the PO_2 will be considerably lower than the stipulated minimum value is scientific rather

than operational. Thus, it can be assumed that any interactions between reduced gravity and low inspired PO_2 as regards physiological responses, will be easier to reveal, if the two stimuli are somewhat exaggerated (i.e. more pronounced unloading and lower oxygen tension) than would be expected in an operational setting. The other rationale for the exaggerated stimuli is that it is premature to try to create a high-fidelity simulation since too many factors regarding the nature and duration of EVAs are as yet unknown. The project will investigate the interactions between hypoxia and unloading as regards several physiological responses. Since the proposed levels of unloading and hypoxia will be exaggerated compared to those encountered during real-life missions, such interactions may be qualitatively rather than quantitatively relevant from an operational perspective.

General background

Until recently, the focus of the preparations for future space missions has been the interplanetary mission to Mars. As a consequence, there has been a heightened interest in the hazardous effects of prolonged exposure to weightlessness, as would be experienced by astronauts and/or cosmonauts during their mission to Mars, as well as an interest in the development of countermeasures to minimise or eliminate such negative effects of weightlessness. The effects of weightlessness have been successfully studied with an experimental model, in which subjects are requested to maintain a horizontal, or slightly head-down position. The changes in the physiological systems during such studies mimic the changes experienced by astronauts and cosmonauts during a sojourn in space of the same duration (Fortney et al. 1996, Convertino 1996).

In 2004, the President of the USA announced a new vision for space explorations, after which the US National Aeronautical Space Agency (NASA) refocused its emphasis towards human missions to the Moon and Mars (NASA 2004). With the Aurora program, the European Space Agency (ESA) formulated similar objectives (ESA 2003, Hufenbach and Seibert 2005, Crawford 2004). As a consequence, some nations with active space programmes have now focussed their attention on establishing human colonies on the Moon. These colonies would exploit the reduced gravity environment, and the mineral resources of the Moon to become financially self-sufficient, and thus lead the progress towards future interplanetary explorations; the emphasis of the NASA program is currently under scrutiny.

The ultimate goal is, of course, to conduct human expeditions to Mars, but not until having acquired adequate knowledge about the planet and not until having successfully demonstrated sustained human exploration missions to the Moon (Mendell 2005). There are many reasons to promote a step-by-step approach as regards extraterrestrial human missions, with short-term Lunar missions preceding long-term Lunar missions and eventually Mars missions. Thus, currently experience relevant to a mission operation of the scale and scope of a human expedition to the surface of Mars is lacking. Moreover, reliability and maintainability of hardware and software systems necessary to undertake a 3-year mission to Mars need to be tested under extraterrestrial conditions, and last but not least, any risks concerning health, safety and performance of the crew need to be investigated and adequate countermeasures against unwarranted effects need to be developed before commencing such missions (cf. Crawford 2004). It should also be noted that sustained human expeditions to the Moon are envisioned to, not only provide knowledge needed for future missions to Mars, but also to increase our understanding of several separate science areas (Mendell 2005, Bodkin et al. 2006).

Planetary habitats

One problem associated with the design of human habitats for use at the surface of the Moon or Mars concerns the environmental control and life support system, which, for both technical and medical safety reasons, is desired to have a low operating pressure (Bodkin et al. 2006). A low operating pressure in the planetary habitat will reduce the potential risk of decompression sickness during extravehicular activities (Scheuring et al. 2008). To avoid any detrimental effects of the resultant hypoxia amongst the crewmembers, the fraction of oxygen must be increased in the habitat gas mixture. The trade-off for restoring the oxygen partial pressure to normoxic levels by increasing the oxygen fraction of the habitat gas mixture is a markedly increased flammability of the gas. A compromise between the elevated oxygen fraction and the reduced environmental gas pressure has been proposed; the environmental gas pressures and oxygen fractions that have been discussed range from 55 to 57 kPa, and from 30 to 40%, respectively (Bodkin et al. 2006). It appears that these values have since been slightly revised; currently, it is envisaged that the gas mixture in a Lunar habitat may have a partial pressure of oxygen (PO_2) of 15.8 kPa (Meck and Conkin, NASA, personal communication). The aim of the present research programme is not to create high-fidelity simulations. This is also not possible since all the parameters for future space flight and sojourns on Mars and the Moon have not yet been precisely defined. The justification for lowering the oxygen fraction in the proposed experiments to the extent that the PO2 will be considerably lower than the stipulated minimum value is scientific rather than operational. Thus, it can be assumed that any interactions between reduced gravity and low inspired PO2 as regards physiological responses, will be easier to reveal, if the two stimuli are somewhat exaggerated (i.e. more pronounced unloading and lower oxygen tension) than would be expected in an operational setting. As mentioned earlier, the other rationale for the exaggerated stimuli is that it is premature to try and create a high-fidelity simulation, since too many factors regarding the nature and duration of lunar EVAs are as yet unknown.

Physiological effects of long-term exposure to reduced oxygen and low gravity

Even though it is well known that humans can acclimatize to such levels of hypoxia (Ward et al. 2000), information is scarce as to how different physiological systems may respond to combined chronic exposure to hypoxia and low gravity force field. As elaborated upon under the section ”Beyond the state of the art (1.2) ”, several significant interactions between prolonged hypoxia and low gravity are envisaged.

Long-term exposure to microgravity brings about mechanical unloading of weight-bearing bones and postural muscles, as well as physical inactivity, resulting in bone demineralisation, muscle atrophy and reduced muscle strength, collectively referred to as musculoskeletal deconditioning (Fortney et al. 1996). In particular, the weight-bearing bones (Rittweger et al. 2009) and the muscles involved in postural control (Berg et al. 2007) are affected. Microgravity also abolishes hydrostatic pressure gradients acting along blood vessels, resulting in redistribution, and subsequently reduction, of the circulating blood volume (Fortney et al. 1996), as well as reduced cardiac function (Convertino et al. 1990, Levine et al. 1997) and increased distensibility in dependant blood vessels (Eiken et al. 2008, Kölegård et al. 2009). These adaptations, commonly referred to as cardiovascular deconditioning, manifest themselves upon return to Earth’s gravity force field as reduced aerobic exercise capacity, reduced tolerance to upright posture and to increased gravitoinertial load in the head-to-foot direction (Fortney et al. 1996), the latter constituting a problem during the space shuttle landing phase (Bassett Frey et al 1990). It can be assumed that chronic exposure to 0.16 G (Lunar gravity) will induce cardiovascular and musculoskeletal deconditioning, which are qualitatively similar to those brought about by

microgravity. From the perspective of physiological adaptation, it is desired that the Lunar/planetary missions be preceded by relevant ground-based simulations.

Possible ground-based simulation of a Planetary habitat

It is well established that prolonged bedrest in the horizontal or slightly head-down position brings about similar cardiovascular and musculoskeletal adaptive responses as microgravity exposure (Fortney et al. 1996). At least as regards the cardiovascular system, it is reasonable to assume that prolonged bedrest in the slightly head-up position (about 9 degrees) may serve as a relevant simulation of a 0.16-G environment. However, many uncertainties remain regarding the gravity and hypoxia stimulus profile during future lunar sorties; these uncertainties include the durations of the microgravity phase as well as the 0.16 G phase, the extent of extravehicular activities, etc. Consequently, it may be premature to try to establish a ”high-fidelety experimental analogue” of Lunar/planetary missions. From a science perspective, it would be preferable to initially focus on basic research regarding the interaction of unloading/inactivity, induced by sustained horizontal bedrest, and hypoxia. Indeed, also life scientists at NASA (see attached letter from NASA) have expressed a wish to be able to undertake prolonged (several weeks) bedrest studies, with the subjects consistently in a hypoxic environment.

Implications for society in general

Knowledge concerning the combined effects of hypoxia and unloading/inactivity is envisaged to also have important implications for society in general. Thus, a growing number of individuals suffer from chronic hypoxia due to respiratory insufficiency, such as advanced chronic obstructive pulmonary disease. Due to their limited physical capacity, these individuals are commonly restricted to an inactive life style, and hence may also suffer from inactivity-related diseases/conditions such as skeletal muscle atrophy and bone demineralisation. To understand the pathophysiology of such conditions, it is warranted to, in a controlled manner, investigate the combined effects of hypoxia and inactivity/unloading.

Feasibility

In 2009 and 2010, a group of life scientists with expertise within the research fields of hypoxia/high altitude and microgravity/bedrest met with scientists from NASA and ESA to discuss: (i) the state of the art on hypoxia and bedrest, respectively, and (ii) whether research on hypoxia combined with bedrest is needed, and, if so, (iii) what experimental paradigm to implement (Mekjavic & Eiken 2010). It was concluded that:

- In view of anticipated long-term Lunar missions, research on hypoxia and unloading/inactivity is needed. Such research is expected to benefit society, in general.
- Due to as yet unidentified operational parameters, it is premature to consider a high-fidelity analogue experimental design; the scientific interest is mainly in the interactions of hypoxia and unloading.
- The experimental paradigm should be based on interventions with horizontal bedrest and normobaric hypoxia with an inspired oxygen partial pressure of about 12.5 kPa.

Having experience with the design and management of both bedrest and hypoxic studies, the partners of the present proposal are cognisant of the complex logistics that are involved in the preparation and management of such research programmes. Furthermore, a study combining bedrest and hypoxia adds a new dimension to the complexity. Accordingly, based on the above recommendations, a feasibility study was conducted, with the objectives to:

- Establish logistics for a hypoxic bedrest study in terms of personnel requirements, organisational structure and modifications required to facility infrastructure.

• Develop emergency-contingency plans in case of technical failure of individual system components.
• Establish the methodology to perform horizontal bedrest within a hypoxic facility, and conduct feasibility trials with human test subjects.

The feasibility study was performed in the hypoxia facility in Planica, Slovenia (see Fig. 2); the facility is capable of housing up to 20 subjects simultaneously at any simulated altitude. In addition to the 10 double rooms available in the hypoxia section, the facility has 25 double rooms available for control subjects, and suites for scientific personnel and staff. The facility also has a large open-plan laboratory in which a hypoxic environment can be established. In addition to investigations on hypoxic system failures and their respective contingency plans, an intervention study was performed, in which four subjects (2 males and 2 females) were exposed to simulated altitudes of up to 4000 m above sea level. Each subject spent 2 days in horizontal bedrest and 2 days in a combination of 9.5° head-up tilt (12 hrs) and horizontal position (during the nights). It was concluded that the facility offers an adequate platform to conduct short-, medium- and long-term hypoxic bedrest studies (Mekjavic & Eiken 2010).

Objectives

The objectives of the present proposal are to evaluate the effects of hypoxia on the deconditioning induced by bedrest. Studies will compare the processes of deconditioning associated with inactivity/unloading in normoxic and hypoxic environments. Since information is scarce concerning interactive effects of such interventions, the study will be explorative in the sense that a broad spectrum of physiological functions will be investigated. To a large extent basic data will be collected, corresponding to that obtained when bedrest experiments are conducted by ESA/NASA (bedrest core data). Since the duration (21 days) of each intervention is identical to that of a medium-term bedrest study according to ESA standards, data from the proposed experiments can readily be compared with core data from previous bedrest studies, and hence the added effects of hypoxia should be apparent. In addition, specific experiments will be conducted to investigate the combined effects of hypoxia and bedrest on:

• cardiovascular and respiratory functions
• musculoskeletal functions
• haematological and immunological functions
• oxidative stress and thermogenesis
• thermoregulatory functions as well as sleep disorders and altitude sickness

Summary. The aim is to investigate the combined effects hypoxia and sustained bedrest on physiological systems.

Scientific novelty and relevance. Humans will be exposed to hypoxia and reduced gravity in future planetary habitats. Information regarding the combined effects of hypoxia and inactivity/ unloading is scarce. To gather such knowledge is not only warranted from a space science perspective, but is also envisaged to increase our knowledge regarding patients suffering from respiratory insufficiency restricting them to a physically inactive life style.

Challenge and feasibility. The challenge of the proposed project lies in the complexity of the experimental interventions, i.e. to, in a controlled manner, confine healthy humans to a hypoxic environment during prolonged, sustained bedrest. The feasibility of the project is ensured by the authors' experience in performing experimental campaigns on prolonged bedrest as well as on hypoxia confinement, and by the successful conductance of a feasibility study on hypoxia and bedrest.

1.2 Progress beyond the state-of-the-art

Cardiovascular and respiratory physiology

It is, as mentioned above, well established that both prolonged bedrest and hypoxia will induce substantial cardiovascular and respiratory adaptations. Information is, however, scarce as regards the interaction of hypoxia and bedrest on cardiovascular and respiratory functions, and consequently, the proposed study will to a large extent be explorative. Several physiological functions, including cardiovascular and respiratory, will be explored by collecting "bedrest core data" (BCD), in accordance with the standards set by ESA for 21-day bedrest campaigns. In addition, certain hypothesis driven projects are proposed. The background and objectives of these projects are outlined below under the headings cardiac functions at rest, pulmonary functions at rest and cardiovascular and respiratory functions during exercise.

Cardiac functions at rest

It is well accepted that both prolonged bedrest and prolonged space flights induce cardiac atrophy and dysfunction. It appears that diastolic as well as systolic functions are affected (Levine et al., 1997, Summers et al., 2005, Platts et al. 2009). The mechanisms underlying bedrest-induced cardiac dysfunctions are not fully understood, but are commonly attributed to adaptive processes resulting from altered cardiac pressure-loading conditions. During exposure to hypoxia, left ventricular systolic function is usually preserved (Boussuges et al. 2000) or even improved (Hirata et al. 1991, Huez et al 2005), whereas, diastolic ventricular functions may be compromised (Huez et al. 2005). Predominantly the right ventricular function appears to be affected, presumably due increased loading, resulting from hypoxia-induced pulmonary vasoconstriction (Boussuges et al. 2000, Naeije 2004, Huez et al. 2005); an elevated pressure gradient between the right ventricle and right atrium may remain even after recompression from altitude to sea level (Boussuges et al. 2000).

To date, cardiac function and dimensions during or following bedrest or hypoxia exposures have mainly been evaluated by use of 2-dimensional echocardiography (2-DE) /standard Doppler techniques, which do not provide reliable evaluations of right ventricular function, due to the complex geometry and nonconcentrical contraction pattern of the right ventricle. By contrast, 3-dimensional echocardiography (3-DE) provides more accurate measurements of volumes and ejection fractions (Gopal et al. 1997, Khaw et al. 2009,), and in combination with tissue Doppler (TDI) imaging of myocardial movement it is currently possible to obtain shape-independent measurement of cardiac ventricular functions (Vogel 2002, Manouras et al. 2009).

Pulmonary functions at rest

Prolonged bedrest, a condition also frequently experienced by critically ill patients, including those with acute respiratory failure requiring mechanical ventilation, can cause several complications that may delay or prevent recovery, including joint contractures and disuse muscle atrophy not only of limb skeletal muscles, but also of respiratory muscles (diaphragm, rib cage muscles, abdominal muscles) that allow the vital function of the ventilatory pump (Truong et al. 2009). Even though these muscles are the only skeletal muscles that provide a vital function (i.e., lung ventilation), only very scanty data are available about the effects of bedrest on them.

The objective of this set of additional measurements to the BCD is to study the effects of normoxic horizontal bedrest, hypoxic horizontal bedrest, and hypoxic ambulatory

confinement on the respiratory system, namely on respiratory muscle and chest wall function at rest and during exercise. Standard spirometry will provide data on overall lung and chest wall function, volitional and nonvolitional tests will provide a quantitative assessment of respiratory muscle force (ATS/ERS, 2002), ultrasonography of parasternals, diaphragm and abdominal muscles (Cala et al. 1998, Ueki et al. 1998, Aliverti et al. 2003) will provide a quantitiative assessment of respiratory muscle thickness at different lung volumes, optoelectronic plethysmography (Cala et al. 1996, Aliverti et al. 2001) will provide data regarding the actions of the respiratory muscles on the different chest wall compartments at rest and during exercise.

Specific objectives and hypotheses: To evaluate the effect of hypoxia and prolonged bedrest, *per se* and in combination, on cardiac systolic and diastolic and on respiratory functions. We hypothesise that exposure to hypoxia will counteract bedrest-induced systolic dysfunctions but aggravate bedrest-induced diastolic dysfunctions We further hypothesise that a) bedrest will alter the breathing pattern at rest and during exercise (decreased action of the rib cage muscles relative to the diaphragm and abdominal muscles); b) hypoxia, causing increased ventilation and work of breathing, will counteract bedrest-induced dysfunction of the respiratory muscles.

Cardiovascular and respiratory functions during exercise

The impairment of skeletal muscle oxidative metabolism in chronic hypoxia is well established (see e.g. Hoppeler & Vogt 2001, Flueck 2009, Calbet & Lundby 2009). In recent years our group, among others, has performed studies aimed to a functional and quantitative evaluation of the impairment of skeletal muscle oxidative metabolism in microgravity, simulated by bedrest (Porcelli et al. 2010, Salvadego et al. 2010c). The effects of the association of hypoxia and microgravity are unknown. These effects, however, are likely to be significant, and could be greater than the sum of those associated with each of the two conditions. This could significantly impair the capacity by humans to carry out work in planetary habitats. A better knowledge of these impairments, moreover, is a pre-requisite for the definition of any countermeasures.

The association of microgravity and hypoxia appears of interest also from at least two other points of view. Whereas in normoxia the respiratory system does not usually represent a significant limiting factor for maximal aerobic function during exercise (as evaluated by VO_2max) (di Prampero 2003), the situation could be quite different in hypoxia (di Prampero 2003). The effects of combined hypoxia and microgravity, also in this respect, are not known, and would be worth investigating, both from a basic science point of view and for the possible practical implications (exercise tolerance, countermeasures, etc.). In hypoxia, moreover, the combined effects of the reduced capacity of convective and diffusive O_2 delivery by the cardiovascular system, and of cerebral vasoconstriction (hypoxia → hyperventilation → hypocapnia → cerebral vasoconstriction) could cause a condition of cerebral hypoxia, which could limit exercise tolerance and contribute to the impairment of aerobic performance (Subudhi et al. 2009). On the other hand, following bedrest the reduced capacity of maximal convective cardiovascular O_2 delivery, which is by definition not associated with hypoxemia, did not cause a “competition” for the available O_2 between skeletal muscle and brain, and did not cause cerebral hypoxia (Porcelli et al. 2010; Salvadego et al. 2010c). The combined effects of hypoxia and microgravity on this aspect are not known, and could obviously be functionally relevant.

Specific objectives: To evaluate the effects of prolonged bedrest and hypoxia, *per se* and in combination, on the O_2 cascade from ambient air to the mitochondria of skeletal muscles.

This will be done by measuring cardiovascular and respiratory variables, fractional oxygen extraction in the in working leg muscles and respiratory muscles and in the brain, as well as muscle respiratory function during different exercise tasks designed so as to limit oxygen availability at the pulmonary, cardiovascular, and muscle oxidative levels, respectively. The consequences of any impairment of the investigated variables/functions, in the various experimental conditions, will be considered and evaluated with respect to exercise tolerance.

Immobilization and Hypoxia; musculoskeletal system

Muscle

Microgravity-induced muscular atrophy in the legs of astronauts (LeBlanc *et al* 2000), and ensuing loss in muscle function (Fitts et al 2010; Narici *et al* 2003) are well documented. Similar alterations result from bedrest (LeBlanc *et al* 1992; Rittweger *et al* 2005), a ground-based model to simulate space flight (Kakurin *et al* 1976) with obvious clinical relevance. Human muscles contain fibres that can be distinguished on the basis of the myosin heavy chain isoform as either slow (type I) or fast (type II). The slow fibres specialize in oxidative metabolism, whilst the fast fibres have a high capacity for glycolytic energy generation (Jones *et al* 2004). Both in response to bedrest, and microgravity, the loss in muscle mass is associated with a shift in fibre types from the slow to the fast isoform (Blottner *et al* 2006; Fitts et al 2010), leading to reduced oxidative capacity. Thus, a recent proteomic approach has demonstrated that the most prominent changes in bedrest are in proteins involved in mechanical stress and in oxidative stress and metabolism (Moriggi *et al* 2010). Responses of skeletal muscle to chronic hypoxia are well documented in terms of metabolic function and bioenergetics (Murray, 2009; Cerretelli, 2009), but less is known about changes in muscle mass, architecture and ultra-structure. There is little evidence to suggest changes in muscle strength in acute or chronic hypoxia (Perrey, 2009), whereas iterative exposures to ischaemic hypoxia in the working muscles may decrease the maximum voluntary dynamic force and increase the share of the muscle cross-sectional area consisting of slow-twitch fibres (Eiken et al., 1991). Moreover, a large body of literature exists to suggest that muscle mass is reduced in mountaineers, because of fibre atrophy (Hoppeler & Vogt 2001), with no change in muscle fibre type (Green et al. 1989), an increase in capillary density (MacDougall et al. 1991), but a decrease in oxidative capacity (Hoppeler & Desplanches 1992). Of note, the decrease in muscle mass is associated with reduction in protein synthesis (Cerretelli & Hoppeler 1996), probably associated with, or caused by, reduced levels of mammalian target of rapamycin (mTOR) (Vigano et al. 2008). Interestingly, high altitude-related atrophy can be circumvented with appropriate nutrition at high altitude (Kayser *et al* 1991). In animals, however, the picture is not clear, as the response to hypoxia may vary between muscles, as well as between regions within a given muscle (Deveci et al. 2001; Faucher et al. 2005; Wust 2008). This could reflect genetic or functional differences between humans and rodents, and certainly also the combined effects of exercise and high altitude acclimation in most human studies. With regard to the latter, relatively little is known about effects of hypoxia in non-exercising people (Hoppeler & Vogt 2001). Furthermore, most of the studies on chronic hypoxia exposure involve some kind of exercise, and most of them lack dietary control. Most importantly, no study has yet investigated the combined effects of hypoxia and disuse.

The latter combination, however, is not only interesting in light of future spacecraft design, but the problem also touches upon a fundamental biological problem. We have found in a previous study that intra-muscular glutathione synthesis is enhanced by bedrest, suggesting that immobilization increases oxidative stress (Agostini et al. 2010). The reason for this

immobilization-related oxidative stress is, as yet, unclear. Firstly, although not likely, it is theoretically possible that immobilization leads to enhanced tissue oxygenation. Tissue oxygenation has been assessed during exercise before and after bedrest (Porcelli et al. 2010), but not under resting conditions during bedrest, There is a possibility that elevated levels of oxidative stress could arise from the degradation of mitochondria, as it is well established that oxidative capacity deteriorates during immobilization. Alternatively, immobilization could lead to mitochondrial leakage, potentially via uncoupling protein-3 (UCP3) (Desaphy et al. 2010).

Thus, it is clear from the above that hypoxia and immobilization have interfering effects upon skeletal muscle. However, the manner in which both stimuli interact remains unresolved (see Figure 3).

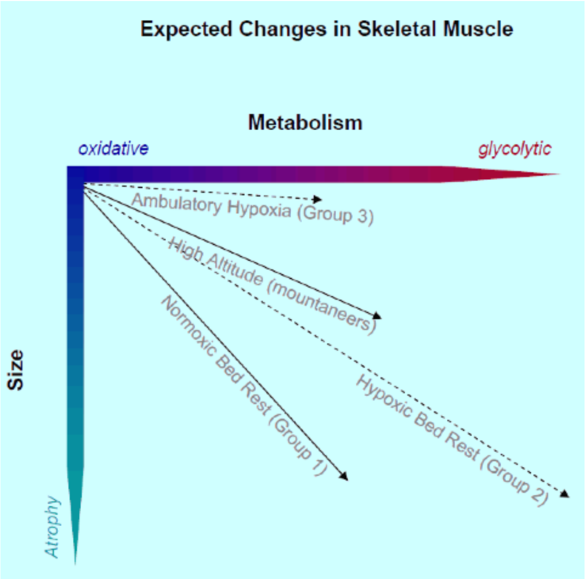

***Figure** 3: Schematic of the changes (muscle size and metabolic specialization) expected in the vastus lateralis muscle of the 3 groups to be studied (Groups 1 to 3), and a comparison with the changes in mountaineers after high altitude sojourns. The dashed lines indicate the hypothesis which will be tested in this project.*

Specific objectives: Investigate the combined effect of hypoxia and inactivity (bedrest) on skeletal muscle structure and function.

Hypothesis: Given that proper alimentation partly offsets the deleterious effects of high altitude exposure, we hypothesize that hypoxia *per se* (a) does not cause any muscular atrophy, but an increased oxidative capacity on superficial layers of the *vastus lateralis* muscle, and (b) the level of bed rest-induced muscle atrophy will be unaffected by hypoxia, but the reduction in oxidative capacity normally observed after bedrest will be exaggerated by hypoxia (see also Figure 3).

Bone

The danger of bone loss in response to immobilization (LeBlanc et al. 1990; Rittweger et al. 2005) and space flight is now widely recognized. Muscles are the generators of the largest forces that our bones experience (Rittweger 2007). Accordingly, resistive exercise is able to prevent bedrest-induced bone losses (Rittweger et al. 2010; Shackelford et al. 2004). Therefore, any alteration in muscle function has the potential to also affect bone metabolism. Very little is known about the effects of hypoxia on bone *in vivo*. On the one hand one could speculate that the respiratory alkalosis, normally associated with exposure to hypoxia, may mitigate bedrest-induced bone losses, either via a renal effect (Frings-Meuthen et al. 2008), or directly via an effect upon the osteocytes. On the other hand *in vitro* experiments suggest that hypoxia *per se* can lead to bone losses by lowering PO_2 and pH and thus foster bone resorption and hamper formation (Arnett 2010). Altogether, one might therefore expect either enhancement or mitigation of bedrest-induced bone losses by hypoxia. Work package 3 will therefore also explore changes in bone metabolism in response to hypoxia and bedrest.

The present proposal will, for the first time, provide an insight into the differential effects of disuse and hypoxia, with nutrition controlled, upon the musculoskeletal system. It is therefore bound to further our understanding of a variety of diseases and disorders. Chronic obstructive pulmonary disease, for example, is characterized by a respiratory disorder that both directly as well as via reduced physical activity and inflammation can have deleterious effects on skeletal muscle (Wust & Degens 2007), and thus will impact on the quality of life as well as upon life expectancy. Another field of direct application and dissemination will be in critical care medicine, where immobilization and various degrees of hypoxia are paramount to therapy success.

Furthermore, the present proposal will fill important gaps in the scientific literature. As mentioned above, it will elucidate the role of hypoxia for muscle and bone metabolism under controlled dietary conditions. Moreover, it will provide further insight into the possible mechanistic role that oxidative stress will play. In order to determine muscle tissue oxygenation, *quantitative near infra-red spectroscopy* (qNIRS) will be used (Tachtsidis et al. 2010) that has been further developed from the now-established NIRS technique. Importantly, qNIRS is able to measure absolute values, rather than relative changes as in the past. A dedicated qNIRS system will be applied in order to establish the extent to which tissue hypoxia occurs under conditions of environmental hypoxia and bedrest (*nota bene:* in the presence of reduced fibre size and unchanged capillary length as well as reduced mitochondrial density, all of which have been reported in response to bedrest, one could even expect increased tissue oxygenation levels).

Finally, the most important progress to be expected is in its applicability to future spacecrafts and human space exploration. Definition of technically feasible levels of oxygen concentration and pressures, together with suitable countermeasures against musculoskeletal de-conditioning constitute the basis for future long-term missions. Candidate countermeasures, such as the advanced resistive exercise device or resistive vibration exercise seem to now come into existence. It can therefore be expected that, at least under normoxic conditions, musculoskeletal integrity can be maintained under microgravity conditions. Therefore, the present research is a necessary step to assess any additional risk arising from hypoxic spacecraft environment. In the affirmative case, scrutiny of any proposed countermeasure has to be done also under hypoxic conditions.

Immunology and Haematology

Space-flight, even when short in duration, can affect the immune system by several adverse effects (“stressors”) like emotional stress, radiation, malnutrition and microgravity which can be aggravated by conditions of hypoxia in future exploration missions. Compromising the immune system may have a significant impact, especially during long-term manned space flights. During two long-term simulation studies (confinement and bedrest) and in cosmonauts studied on the International Space Station (ISS) we have observed considerable immunological changes which were paralleled by increased levels of emotional stress and stress hormone secretion (Choukèr et al 2002, 2004, Kaufmann 2008, Crucian et al 2008). The changes of the non-specific and specific immune system showed altered reactivity of granulocytes towards cell-receptor-induced oxidative burst stimulation, changes of T-cell functions and overall impaired reactivity to microbial antigens detected by delayed type hypersensitivity *in vitro* tests (Choukèr 2010, submitted). These observed immune responses are thought to be become aggravated under hypoxia, with the latter becoming an important stressor relevant for future manned exploration.

Although much work has been reported regarding the effect of high altitude on immune system function, there is still a lack of understanding regarding the manner in which immunity is affected by endogenous stress response systems under conditions of controlled hypoxia, especially when the body is subjected additionally to immobilization, shown to affect innate immune functions (Chouker et al. 2002).

Hypoxic stress affects immunity

Stress and stress-response systems contribute to immune control. The complexity of control of the immune system is susceptible to a disequilibration of stress-response systems which may result in deleterious side-effects for risk of infection and of disease. The control of these critical immune functions when challenged by microbes and viruses are redundant and include auto-, para- and endocrine signalling [Tracey 2002]. Among those stress response systems, (hypoxia sensitive) hormone- or hormone-like acting mediators (e.g. corticoids, catecholamines, endocannabinoids and purines) modulate immune responses. From the literature and from own recent investigations we and other could confirm the role of the **glucocorticoid-** (Glaser et al.), the **catecholaminergic-** and the **endocannabinoid-** system (Choukèr et al. 2010), as important stress response system to affect innate and adaptive immune responses (Kaufmann et al 2008), including neural pathways to control the host´s immune response. Altogether neural, hormonal and inflammatory signals regulate the brain-immune axis (Klein et al. 2003), which exerts an important pathophysiological control of inflammation (D'Argentino et al. 2006). In addition, because lowered oxygen tension is a major, evolutionary preserved variable to control immune function through very hypoxia sensitive stress response systems affecting the purinergic system, this crossover study protocol in healthy subjects is of unique nature to understand the role of the stress response systems under conditions of controlled hypoxia, especially when the human organism is subjected to immobilization which may further affect the immune imbalance.

Breathing hypoxic air and delivering reduced amount of oxygen to the tissues can result in hypoxia-induced modulation of the above mentioned stress response systems and of the immune system.

One especially hypoxia sensitive stress-permissive response system is the purinergic system with its main mediator adenosine. The purinergic signalling system is one of the most ancient and arguably the most widespread intercellular signalling system in living tissues. Adenosine acts on four different and widely distributed cell surface adenosine receptors: A1, A2A, A2B, and A3 (Linden 2001). It is known that hypoxia (10%) promotes

the accumulation of extra-cellular adenosine as a result of enhanced purine nucleotide degradation from adenosine tri- and diphosphate (ATP, ADP) to adenosine-monophosphate (ADP) and finally to adenosine (ADO) (Decking et al. 1997; Chouker et al. 2005, 2008). Binding of adenosine to the cAMP-elevating G_s protein-coupled A2 receptors results in an inhibition of effector functions of T cells and myeloid cells and includes the inhibition of expansion and secretion of cytotoxic molecules and cytokines (Choukèr et al. 2008). Moreover, by using e.g. adenosine A2A receptor gene deficient mice (Ohta et al. 2001) it was show that adenosine can modulate the functions of a large spectrum of cells of the immune system, to decrease cell adhesion, the synthesis of cytokines (e.g. tumor necrosis factor-α) (Thiel et al. 2005; Chouker et al. 2008) and T-cell receptor dependent signaling with a shift of T-helper cells to suppressor/cytotoxic T-cells. Vice versa, like hypoxia or the release of cytokines (Kreth et al. 2009) influence expression of purinergic receptors and its sensitivity.

Accumulation of immunosuppressive intracellular cAMP in immune cell - a prime pathway as for the action G-protein coupled action of adenosines, catecholamines and endocannabinoids as well - inhibits intracellular distal signalling pathways by down-regulating the synthesis and secretion of pro-inflammatory and cytotoxic mediators by immune cells (Condie et al. 1996; Sitkovsky 2003). This terminates immune cells' effector functions and thereby leads to the down-regulation of the immune response (Thiel & Choukèr 2005, Sitkovsky et al 2005).

Interestingly, there are important mutual interactions between other hematologic 'non-immune factors' like erythropoetin (EPO) and adenosine (ADO) during hypoxia:

• Hypoxia induces EPO dependent erythropoesis to compensate for hypoxia impaired oxygen supply.

• Hypoxia stabilizes the hypoxia inducible factor (HIF) in immune cells.

• Production of EPO is amplified through adenosine A2 receptor activation (Ohigashi et al. 1995). Therefore EPO can reduce the Hypoxia-adenosine and the hypoxia-HIF mediated immune suppression hereby possibly counterbalancing also the effects hypoxia-HIF induced immune suppression (Katz et al. 2007).

Thus, it needs to be investigated if the relation between EPO and ADO can be considered as a critical factor linked to degree of immune suppression, as a low ratio of EPO/ADO might be- under conditions of hypoxia- be positively correlated to immune suppression.

Beside an integrated set-up for the assessment of degree of stress and stress responses on the level of perceived stress and cognitive dysfunction (questionnaires) and biological markers and assays for immune function, we the proposer plan, in addition to beside blood collections, the application of a non-invasive technique of breath analyses by Ion Molecule Reaction (IMR)-Mass-Spectrometry (MS). This IMR-MS represents a scientifically attractive and complementary breakthrough for longitudinal monitoring. In the recent decade it has emerged that many different volatile organic compounds (VOC) as well as volatile inorganic compounds (VICs) are present in the exhaled breath and that some of these compounds can serve as indicators for multiple physiologic (Millonig et al. 2010: Hornuss et al. 2007]. This method will be important for the quantification of immune re-activity and oxidative stress as reflected by changes in diens, aldehydes, NO, propionaldehyde, isoprene, pentane and VOCs and VICs (Dolch et al. submitted 2010]. These markers will be correlated to the blood borne parameters taken at the same time.

Specific objectives: Stress and hypoxia sensitive response systems will be analysed using a battery of standard and innovative tools to describe additive or counteracting stress responses and their consequences on the immune systems´ function.
This integrative approach under standardized conditions will provide: i) new insight and also a mechanistic understanding of the complex interaction of psycho–neuro-endocrine, metabolic and physical stress factors (hypoxia) and impact on immunity in the active and inactive (bedrest) healthy volunteers, and will ii) provide the necessary understanding for tentative measures to preserve or even restore adequate immunity under such conditions.
Hypothesis: Hypoxia stress will induce an activation of the stress-response systems and hereby induce altered immune responses. This condition is further be aggravated by hypoxic bed-rest as compared to normoxic bedrest.

Oxygen availability, oxidative stress, glutathione system and muscle atrophy

Oxidative stress originates when free radical production exceeds the antioxidant scavaging defenses within an organism. Several classes of macromolecules can be modified by the action of free radicals; beside lipid membranes (Ghosh et al. 1993) and nucleic acids (Beckman & Ames 1998), proteins are one of the most important target molecules of free radical action.

Protein carbonylation as marker of oxidative stress

Protein carbonylation has been demonstrated to be a reliable marker of oxidative stress (Greilberger et al. 2008). Free radical actions on proteins can modify selected amino acids via the stable addition of carbonyl groups (Roth et al. 2004). Protein carbonylation, obtained by complex and irreversible mechanisms (Dalle-Donne et al. 2003), can alter both enzyme structure and activity (Stadtman 2001). Indeed, increased levels of protein carbonylation were shown in patients affected by neurological diseases such as Alzheimer and Parkinson’s, and in myopathies such as Duchenne muscular dystrophy or amyotrophic lateral sclerosis (Stadtman 2001). Interestingly, high protein carbonylation levels have also been demonstrated in the respiratory muscles of chronic obstructive pulmonary disease (COPD) patients (Barreiro et al. 2005) and are significantly associated to the disease progression in muscle wasting patients with leukemia (Ahmad et al. 2008)

Oxidative stress, proteolysis and muscle atrophy

Oxidation of sulphydril groups can fragment protein structure (Stadtman 1990; Starke-Reed & Oliver 1989) and oxidative stress is known to be involved in the regulation of complex pathways leading to protein and muscle wasting. It can, in fact, induce perturbations of intracellular ionic homeostasis (Kondo et al. 1994) while reactive aldehydes (i.e. 4-hydroxy-2,3-trans-nonenal) can decrease $Ca2^{+}$ removal from the cell (Siems et al. 2003). Thus, increased intrecellular calcium concentrations can trigger calpains (Primeau et al. 2002) and other calcium-activated proteases augmenting cytoskeletal proteolysis and degradation of myofilaments by proteasome (Tidball & Spencer 2002; Goll et al., 2003). Oxidative stress can induce skeletal muscle atrophy, controlling co-factors of caspase-3 activity (Primeau et al. 2002) and can directly affect muscle protein degradation at the proteasome level (Betters et al. 2004). Specifically, oxidative stress has been shown to upregulate the expression of muscle atrophy F-box/atrogin1 and MuRF-1 in myotubes (Li et al. 2003), and with the increased expression of such E3 ubiquitin ligases in skeletal muscle, can enhance proteolysis and muscle atrophy (Bodine *et al.* 2001). Furthermore, carbonylated proteins can be selectively degraded by the 20S core proteasome without ubiquitination (Grune *et al.* 2003; Grune & Davies, 2003). To avoid accumulation of damaged peptides, carbonylated

proteins can be very rapidly scavenged by proteolytic degradation compared to their nonoxidized counterparts (Dukan et al. 2000; Bota & Davies 2002; Grune et al. 2003).

Inactivity and oxidative stress: impact on muscle atrophy

Low levels of physical activity can promote oxidative stress. Animal studies have shown that inactive confinement compared to constant training increases lipid peroxidation and reactive oxygen species (ROS) release (Laufs et al. 2005). Available evidence suggests that inactivity-mediated oxidative stress can promote muscle atrophy as a typical consequence of immobility. In particular, experimental unloading of the soleus muscle increases lipid hydroperoxide levels, oxidation of selected target substrates (Lawler et al. 2003), while decreasing catalase and glutathione peroxidase, and non-enzymatic antioxidant capacity (Kondo et al. 1994; Lawler et al. 2003; Kondo et al. 1992). Muscle unloading is shown to induce a decrease in antioxidant heat shock proteins and glutathione peroxydase activity (Lawler et al. 2006). Other published data showed that muscle unloading can up-regulate a heme-oxygenase response in virtue of previously occurring oxidative damage (Hunter et al. 2001). Investigations into the alterations of gene expression in unloaded muscle have also demonstrated that factors promoting oxidative stress, ubiquitination and protein degradation are significantly up-regulated by immobility (Stevenson et al. 2003). Interestingly, lowered oxidative stress induction in unloaded muscles, achieved by supplementation of antioxidant vitamin E, can reduce protein wasting and muscle atrophy (Appell *et al.*, 1997). Even though the general association between inactivity, oxidative stress and muscle atrophy is well published, specific knowledge of the exact mechanisms between inactivity and oxidative stress is incomplete. It is plausible that inactivity can trigger muscle oxidative stress (e.g. protein carbonylation) by the interaction of different sources of oxidative species (Kondo et al., 1993).

Antioxidant activity of glutathione

The ability of an organism to counteract free radical action is exerted by both enzymic and non-enzymic factors. The most important non-enzymatic antioxidant in the organism is glutathione: it is a thiolic tripeptide formed by glutammic acid, cysteine and glycine (Pastore et al. 2003). The antioxidant activity of glutathione is primarily based on the action of glutathione peroxidase which catalyzes the conversion of hydrogen peroxide to water. Essentially, glutathione peroxidase involves the oxidation of 2 molecules (reduced glutathione to form the glutathione dimer) (Pastore et al. 2003) which allows for the preservation of a protein’s structure and function. Glutathione depletion is frequently encountered in pathologies associated with oxidative damage, including: liver cyrrhosis (Altomare et al. 1988), COPD or acute respiratory distress syndrome (Anderson, 1997) and cardiovascular pathologies (Morrison et al. 1999). Thus, increased availability of glutathione can be considered, at least in healthy subjects, as an active response to a previous release of free radicals.

Glutathione and physical activity level

Regular exercise can enhance total and reduced glutathione availability (Sen & Packer 2000). The ratio between reduced and oxidised glutathione, a reliable marker of glutathione system activation, is increased by exercise (Ji 1995). Physical training has been shown to induce glutathione incorporation in muscle from blood, enhance *de novo* synthesis, and the release of glutathione from the liver was also observed as a consequence of regular physical activity (Leeuwenburgh & Ji, 1995). Activity of enzymes related to glutathione metabolism (glutathione peroxidase and glutathione reductase) were also up-regulated by moderate exercise (Ortenblad et al. 1997): demonstrating that the glutathione system may be activated by muscle contractions. In long-term training studies glutathione oxidation rate

was decreased in the trained compared to the untrained subjects when performing a single bout of exercise (Michelet et al. 1995). Several bouts of exercise can upregulate oxidized glutathione levels, especially in trained subjects (Sastre et al. 1992). These results suggest an upregulation of glutathione availability and enzymatic efficiency in trained subjects (Kretzschmar et al., 1991). Interestingly we previously showed in human volunteers that bed rest can effectively enhance whole body glutathione availability as a response to previous production of oxidative species (Biolo et al. 2008).

Muscle Glutathione synthesis rate assessment
In this project a new validated approach will be used to assess muscle glutathione synthesis rate in healthy humans (Agostini et al. 2010) involving two parallel infusions of different isotopes of the same amino acid precursor of glutathione. Infusions are started with a calculated time shift and terminated concomitantly with muscle biopsy sampling. The measurement of the two different isotopic products, as reflecting different times of precursor infusion and incorporation, allows for the determination of glutathione synthesis rate in muscle.

Oxygen availability and oxidative stress
Oxygen is crucial for the survival of eukaryotic cells through its critical role in energy metabolism as terminal energy acceptor. Nevertheless, oxygen free radical byproducts of this process can directly induce damage on several substrates, including proteins. Molecular oxygen availability can significantly modulate the production of reactive free radicals. In vitro studies have shown that molecular oxygen availability can affect oxidative injury; reactive oxygen production (Cadenas *et al.*, 1983) and cytochrome oxidation (Jones & Mason, 1978) have been linearly correlated to oxygen availability, and there is an indirect correlation between reductive processes and oxygen availability (Jones & Kennedy, 1982). Hyperoxia, ie. exposure to increased oxygen partial pressure, has been shown to induce oxidative stress. H_2O_2 production by heart and liver mitochondria is upregulated by increased oxygen availability (Boveris & Chance, 1973) while under hyperoxic conditions, mitochondrial reactive oxygen species generation increase linearly with oxygen concentration (Turrens et al. 1982a). Still, an acute increase of oxygen concentration can induce significant synthesis of total glutathione (Turrens et al. 1982b) suggesting that hyperoxia can stimulate a cytotoxic effect, mediated by the increased production of reactive oxygen species. Severe hypoxia is also associated with oxidative stress and demonstrated to be associated with significant oxidative stress-induced endothelial damage (Karimova & Pinsky, 2001,Motterlini et al. 2000). In contrast to hyperoxia and severe hypoxia, recent studies indicate that moderate hypoxia can paradoxically improve oxidative stress (Rascón & Harrison 2010). The mechanisms for these inconsistencies are presently unknown, however evidence indicates that moderate hypoxia may enhance glutathione levels (Lin & Miller 1992) potentially leading to an improved re-dox balance.

Specific objectives: 1) To describe, in healthy volunteers, and under controlled nutritional conditions, changes mediated by combination of physical inactivity and reduced environmental oxygen availability on: a) glutathione synthesis and oxidative stress at whole body (blood) level and in muscle biopsies and b) muscle atrophy.
2) To provide new insights for innovative therapy of COPD patients, which are characterized by low physical activity levels, hypoxia, increased whole body oxidative stress and muscle atrophy.
Hypothesis: Inactivity combined with moderate reduction of oxygen availability can positively interact to increase muscle glutathione synthesis leading to reduced oxidative stress development and lowered muscle atrophy.

Thermogenesis, adipocyte growth and insulin resistance under hypoxic conditions

Adipocytes play a key role in energy balance by serving as a major site of storage, and as an endocrine organ, secreting molecules that regulate energy storage and metabolism in other tissues as well (Spiegelman and Flier 2001). Along with skeletal muscle cells, adipocytes are the main target for insulin action to lower the blood glucose level by stimulating the transport of glucose from plasma into the cell. Insulin also acts to increase the energy storage capacity by increasing the cell size of adipocytes (Jeanrenaud 1978). It was proposed that an insulin-induced increase in membrane area may contribute to the growth of single adipocytes during lipogenesis (Chowdhury et al. 2005). Recently, it was shown that small and large adipocytes differ in their protein expression at key steps in lipid and energy metabolism related to insulin action and cellular lipid accumulation (Blüher et al. 2004), which may play an important role in the development of type 2 diabetes. Interestingly, enlarged adipocytes predict type 2 diabetes (Weyer et al., 2000) and treatment with agonists for peroxisome proliferator–activated receptor gamma (PPARγ) improves insulin resistance and increases the number of small adipocytes in fat tissue (Rangwala and Lazar, 2004)). These studies indicate that the growth of single adipocytes is an important process in the development of metabolic abnormalities, but the mechanism of cell enlargement is still poorly understood.

Hypoxic stress plays a pivotal role in normal human development and physiology and also in the pathogenesis of several human diseases, including heart disease, stroke, diabetes and cancer (Semenza 2000). Recent studies provided consistent evidence that adipose tissue hypoxia (ATH) exists in obese mice and that it contributes to the initiation of chronic inflammation and inhibition of adiponectin expression in the white adipose tissue (Hosogai at al, 2007; Rausch et al, 2008; Ye et al, 2007). Hypoxia was shown to enhance insulin-independent glucose uptake in human adipocytes through induction of GLUT1 expression in mRNA and protein (Wood et al, 2007). On the other hand, it was shown that hypoxia decreases insulin signaling pathways in adipocytes leading to a decrease in glucose transport by inhibiting the insulin receptor tyrosine phosphorylation and reducing insulin-signalling molecules IRβ and IRS-1 (Regazzetti et al. 2009; Yin et al. 2009). The second study also proposed evidence that hypoxia is able to induce strong lipolysis in adipose tissue in vivo and decreases the expression of transcription factors, such as PPARγ and C/EBPα and fatty acid transporters FATP1 and CD36 (Yin et al, 2009), suggested that the reduction may be a result of chronic hypoxia response. However, the effect of hypoxia on the growth of adipocytes has not been yet reported. Additionally, the relationship of ATH and changes in membrane surface area of single adipocytes was not characterized.

Specific objectives: To describe, in healthy volunteers and under controlled nutritional conditions, changes mediated by combination of physical inactivity and reduced environmental oxygen availability on: (a) adipose tissue expression of inflammatory genes and alterations in metabolic genes associated with insulin resistance, (b) dipocyte membrane potential and adipocyte proliferation
Hypothesis: Acute exposure to hypoxia and inactivity will have specific effects on adipose tissue, by increasing the expression of inflammatory cytokines, that will contribute to the development of whole body insulin resistance.

Temperature regulation, sleep disorders and altitude sickness

Exercise temperature regulation

Based on the physiological evidence provided by decades of manned spaceflights, prolonged exposure to microgravity does not appear to jeopardize the maintenance of normothermia in astronauts. Results of limited thermal studies on flight missions suggest a decrease in the sensitivity of the heat loss response in humans (Fortney et al. 1998) and primates (Sulzman et al. 1992). This has been confirmed by bedrest and post-flight experiments, noting that inactivity or prolonged exposure to microgravity causes impairment in exercise thermoregulation (Fortney 1987; Lee et al. 2002; Crandall et al. 1994). Specifically, there is a greater exercise-induced increase in core temperature (Greenleaf and Reese, 1980; Greenleaf, 1980), attributed to impaired sweating (Greenleaf and Reese, 1980; Fortney, 1987; Fortney et al. 1998; Lee et al. 2002) and vasodilatory responses (Williams and Reese, 1976; Crandall et al., 1994; Fortney et al. 1998; Lee et al. 2002). These alterations in exercise thermoregulatory responses have been attributed to non-thermal factors associated with prolonged bedrest.

The reported changes in the sweating and vasodilatory responses following bedrest are most likely attributable to the changes in body fluid compartments. Namely, it is well documented that both hypovolemia and dehydration, conditions present in subjects during various phases of bedrest, attenuate the magnitudes of the vasodilatory and sweating responses. Hypovolemia has been demonstrated by Fortney et al. (1981) to significantly attenuate the sweating response. Specifically, an 8% decrease in blood volume significantly reduced the magnitude of the exercise sweating response and attenuated the gain of the sweating response (Esw) relative to increases in core temperature (Tc). This reduction in the Esw/Tc response was observed only on the skin of non-exercising regions. The sweating response did not appear to be affected in the exercising regions. Similar to the effect of hypovolemia, dehydration also attenuates the sweating response (Ekblom et al. 1970; Candas et al. 1986; Grucza et al. 1987). It is the increased plasma osmolality, associated with dehydration that is sensed by the hypothalamic osmosensitive neurons (Silva and Boulant 1984), that in turn modulate the thermoregulatory responses (Baker and Doris 1982 a,b). In the event that the conditions of dehydration and hypovolemia occur concomitantly, then the effect on thermoregulatory responses has been reported to be additive (Fortney et al. 1981, Baker 1984).

Sleep temperature regulation

Both bedrest and hypoxia induce peripheral vasoconstriction (Golja et al. 2002, 2003). Whereas the reduction of peripheral perfusion, as monitored with infra-red spectrometry, increases during the period of bedrest, the hypoxia-induced vasoconstriction presumably diminishes during prolonged exposure to hypoxia, most likely due to the adaptation to the

hypoxic environment. Sleeplessness, one of the symptoms of altitude sickness, may be associated with the hypoxia-induced vasoconstriction. According to Krauchi et al. (1999) thermal afferent information from the feet, particularly from the warm sensors, promotes sleep onset and enhances sleep quality. The enhanced heat loss from the peripheral regions observed during sleep, is suggested to provide the thermal afferent stimulus for sleep onset (Krauchi & Wirz-Justice, 1994; Krauchi et al. 2000). This would therefore suggest that the progressive reduction in peripheral perfusion during bedrest progressively also affect sleep quality. In contrast, the reduction in sleep quality associated with the hypoxia-induced reduction in peripheral perfusion would be less with progressive adaptation to the hypoxic environment. The combined effect of hypoxia and bedrest would presumably induce a much greater and sustained reduction in sleep quality.

Equivalent air altitude

As a result of Paul Bert's investigations during the third quarter of the 19 century it has become axiomatic that combinations of ambient pressures and oxygen fractions that produce the same partial pressure of oxygen in the inspired gas will cause the same physiological response (Bert 1878). However, the equivalent air altitude model, as it has become known as, has been questioned both in regards to extreme hypoxic exposures (Rahn & Fenn, 1956), and also in regards to less severe hypoxic exposures (eg. Roach et al, 1996). In a recent review (Conkin & Wessel III, 2008) it was noted that in a number of studies on humans the acute ventilatory effect of hypoxia differ between normobaric and hypobaric (altitude) hypoxia (eg. Loeppky et al 1996, Savourey et al 2003).

These latter studies both showed subjects having a higher respiratory rate during altitude exposure compared to normobaric hypoxia, and a tendency to lower arterial oxygen saturation at altitude compared to normobaria. The mechanisms causing the difference between normobaric hypoxia and hypobaric hypoxia have not been elucidated, but most authors suggest that the different gas densities could be the cause for a change in respiratory pattern (Loeppky et al. 1996, 1997, Savourey et al. 2003). This hypothesis has never been tested, though.

Using light (He) or heavy (SF_6) inert gases to balance the density of the breathing gas, it would be possible to expose subjects for hypoxic gas at normal pressure with the same low gas density as at altitude, and conversely expose subjects for altitude hypoxia but retaining a normal density of inspired gas. A test of the equivalent air altitude model, and its possible dependency on gas density, would be of importance for future high fidelity tests of the Lunar and Mars expeditions, since it would reduce the necessity to carry out such tests in altitude chambers.

Specific objectives: Assess the combined effect of hypoxia and inactivity on autonomic and behavioural thermoregulatory function during rest, exercise and sleep, and document the risk of altitude sickness during the hypoxic bedrest intervention.
Hypothesis: It is hypothesised that: (1) hypoxia enhances bedrest-induced alterations in thermoregulatory function; (2) hypoxia-induced peripheral vasoconstriction contributes to the sleep disturbance often associated with altitude sickness, and (3) the progressive vasoconstriction observed in the extremities during bedrest gives progressive rise to sleep disturbances.

1.3 S/T methodology and associated work plan

i) Overall strategy of the work plan.

To address the problems and specific objectives outlined above, the following Workpackages (WPs) are defined:

WP1: Hypoxic bedrest facility and protocol
WP2: Effects of hypoxia and bedrest on cardiovascular and musculoskeletal function
WP3: Effects of hypoxic inactivity on the musculoskeletal system
WP4: Monitoring of stress and hypoxia sensitive immune and haematological changes
WP5: Hypoxia and physical inactivity regulation of oxidative stress and thermogenesis
WP6: Hypoxia-induced thermoregulatory dysfunction, sleep disorders and ventilator equivalent altitude

Further WPs are
WP7: Dissemination
WP8: Management

All physiological data and tissue samples required by WPs 1 to 6 will be obtained in the course of three bed rest/confinement campaigns, each one lasting 4 months (including pre-trial conditioning, 21 days of bed rest, and a recovery period), which is the objective of WP1: i) hypoxic bedrest; ii) normoxic bedrest; iii) hypoxic confinement with ambulation. In addition to the basic physiological core data which will be obtained during each study, individual principal investigators (PIs) and their respective staff will be accommodated in the Planica facility, and a workstation/laboratory established, so that they may collect physiological data specific to their studies. Most of the data collection will occur prior to, and immediately after the completion of each bedrest. Nevertheless, some measurements will also be made during the course of the bedrest/confinement and recovery periods. The experimental schedule is presented in detail in WP1. Additional analysis of the data, biological tissues and samples will be conducted within the framework of WPs 2 to 6. Training of personnel and students, and dissemination of the results of the project to the scientific and lay communities is the objective of WP7. Finally, WP8 will deal with the administrative, and financial management of the project, as well as management of ethical issues. It also outlines the manner in which the bedrest studies will be managed at the Planica facility.

Year 1: It is envisaged that within the proposed 3-year project, one year will be devoted to: preparation of documentation for submission to the Ethics Committee, training medical and technical staff for allocated tasks in the bedrest campaigns, establishing normoxic and hypoxic laboratories, establishing the Management structure (WP8), and subject selection, and conducting familiarisation trials. During the familiarization trials subjects interested in participating in PlanHab will be invited to Planica to be briefed by the scientific personnel regarding the instrumentation, methodology and aims of the experimental studies. On the basis of these familiarization trials, the final subject selection will be conducted.

Year 2: Once the final subject selection is made, preliminary trials and the experimental study comprising three bedrest campaigns will be conducted in years 1 and 2 of the study. Although the duration of each bedrest/confinement campaign is only 21 days, subjects will be monitored for 14 days prior to the bed rest and during the 4-month recovery period. The recovery phase is an important, but often neglected, phase of the campaign, as it provides information regarding the rate of recovery of individual physiological systems to pre-bedrest baseline values.

Year 3: The final year of the study will be devoted to the analysis of the data and preparation of deliverables. The focus will be on dissemination of the results of the study as outlined in WP7.

Graphical presentation of the components showing their interdependencies (Pert diagram)

Workpackage 1 forms the backbone of the project, as shown in Fig. 4. It is the essential component of **all** workpackages, and is also the foundation for the entire project. Although indpendent entitites, the WPs are also interconnected. Thus, all WPs will derive data from the interventions studies, which is the focus of WP1. In addition, each WP will also derive data from additional experiments, which will be conducted during the main interventions studies in WP1, and this data will also be shared among the other WPs.

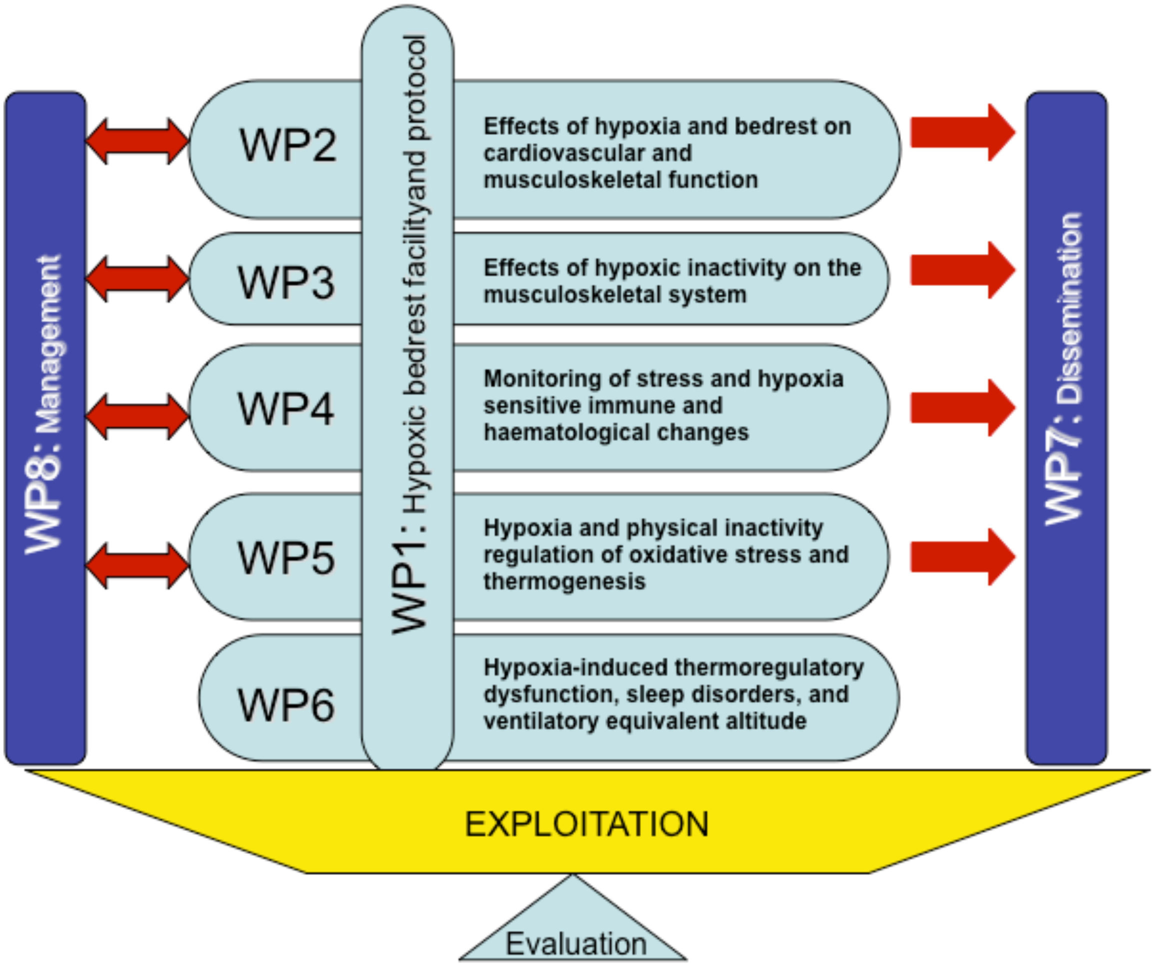

Figure 4: *Pert diagram of the workpackages and their interdependencies.*

As indicated in Fig. 4, Dissemination of the knowledge from PlanHab will also be of prime concern, and innovative initiatives will be developed for this purpose, as presented in WP7. Exploitation of the results will also be an important aspect of the project. As presented in Section 2, a consortium of European (EU) small-and-medium enterpriseses (SMEs) are already interested in exploiting the potential results of PlanHab.

Description of significant risks and contingency plans

Standard policies and procedures for Risk Management will be used. Risk is understood to be the product of damage and probability of damage occurring. The risk assessment procedure is outlined below in Fig. 5.

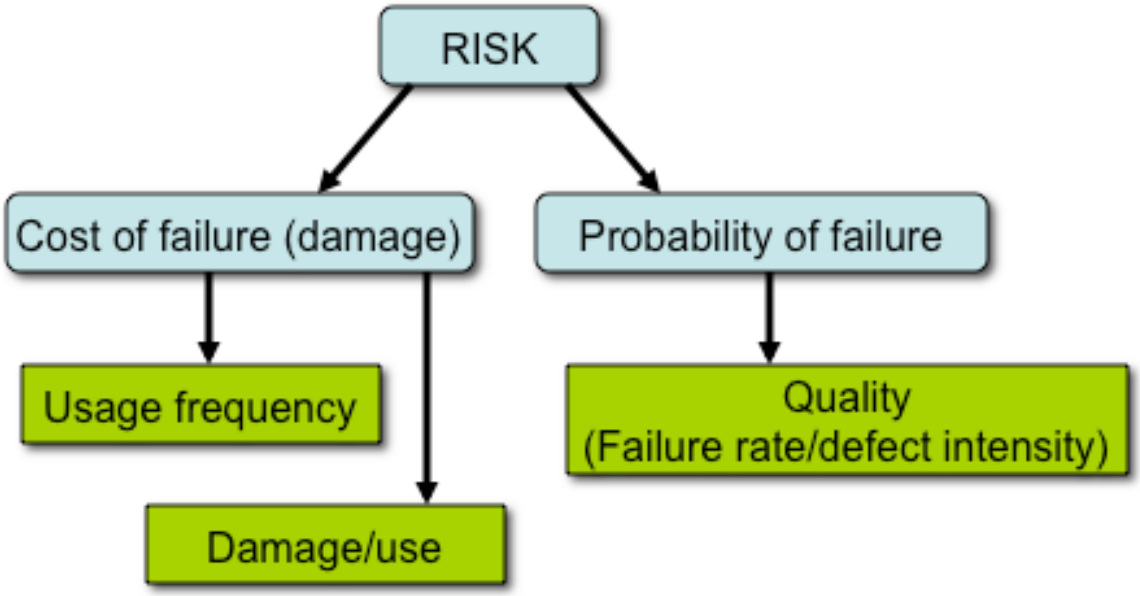

Figure 5: *Risk definition and structure*

The Risk Impact will be quantified according to the criteria in Fig. 6.

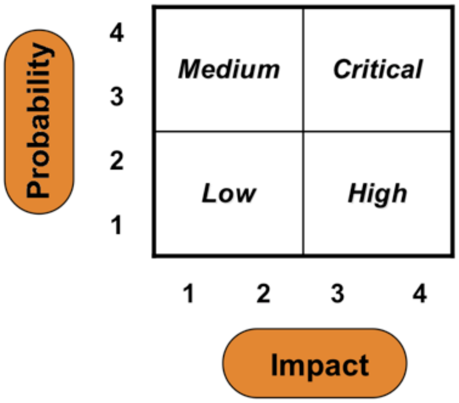

Figure 6: *Risk quantification*

Based on above risk analysis, the risks detailed in Table 2 were identified, rated, and contingency plans established.

Table 2: Potential risks and contingency plans.

Potential risks (unexpected outcomes)	Rating	Contingency plan
**WP1:** Hypoxic Bedrest Facility and Protocol		
**Risk 1.1:** Failure to reach consensus regarding the simulation paradigm and/or the nature of core data.	**Low**	During the pilot study workshops, all PIs agreed to the adoption of the guidelines outlined in ESA documents: ***"Standardization of bed rest study conditions".***
**Risk 1.2:** Failure to establish guidelines for core data measurement, analysis, storage and management.	**Low**	During the pilot study workshops, all partners agreed to the adoption of the guidelines outlined in ESA documents: ***"Standard Operating Procedures for Bed Rest Core Data Using Bed Rest Studies".*** Also, the coordinator has experience coordinating large international bedrest and hypoxia studies, which have required appropriate data management. All PIs have participated in international bedrest studies.
**Risk 1.3:** Failure of equipment in the hypoxic facility	**Low**	In case of failure of the permanent swing absorption system, portable back-up systems will be available in each room. A failure analyses study has been performed in which capacity of the back-up system was tested and found to perform sufficiently. Judging from the flawless function of the hypoxia systems to date, the risk for failure seems remote. An equipment failure analysis has been conducted within the framework of a pilot study. Where indicated, appropriate back-ups will be implemented.
**Risk 1.4:** Failure to establish the infrastructure needed to perform successful hypoxic and normoxic bedrest and confinement campaigns.	**Low**	JSI and KTH have already organised 5-week horizontal, and head-down bedrest studies, as well as 4-week hypoxic training studies. In

		June 2010, a pilot study was conducted with 4 subjects (2 males and 2 females) exposed to combinations of head-up and horizontal bedrests at simulated altitudes to 4000m above sea level, in the Plancia facility. A workshop on the topic of the Feasibility study identified infrastructrue and logistic bottle-necks, which have been addressed, such that their potential impact on the success of the study is low.
**Risk 1.5:**<br>Unforeseen adverse medical events	**Low**	During the interventions, test subjects will be observed 24 hrs/day by nurses, and checked on a daily basis by medical doctors. On a few previous occasions hypoxic bedrest studies have been performed without any adverse events (Stevens et al 1966).<br>Moreover, in preparation of the present proposal we have performed a feasibility study in which subjects (n=4) were exposed to horizontal bedrest and hypoxia (corresponding to an altitude of 4000 m above sea level) at the Olympic Centre in Planica; no adverse advents were noted.
**Risk 1.6:**<br>Subject “drop-out”	**Medium**	The commitment for each subject will be substantial, both with regard to time and effort, and hence the risk that subjects will discontinue their participation prematurely must be considered. The research group performing WP 1 has long experience of performing sustained bedrest studies of 5-wk durations, as well as of performing hypoxic confinement studies and hypoxic physical training studies. In these studies, the number of subjects terminating prematurely have been

		marginal. Also, the number of subjects (20) is gauged so as to permit a "drop out" of 8 subjects, while maintaining sufficient statistical power. Should the "drop-out" number exceed 8, additional subjects will be recruited and the study design will convert from intra-individual to group comparisons.
**WP2:** Effects of hypoxia and bedrest on cardiovascular and respiratory functions at rest and during exercise		
**Risk 2.1:**<br>Failure to receive ethics approval for obtaining muscle biopsies**.**	**Low**	Ethics approval for withdrawing muscle biopsies has been obtained previously for two bedrest studies.
**Risk 2.2:**<br>Failure to obtain sufficient muscle tissue during the biopsy procedure.	**Low**	The standard operating procedure utilised to date by individual partners (DLR, JSI, KTH, UoT, UoU) has provided sufficient tissue sample size for the required analyses. In case that the first approach with the biopsy rongeur fails, we will use a smaller automatic needle system, which in our hands has 100% success. The only real risk therefore would be that subjects do not tolerate the biopsy for emotional reasons. This risk will be met by defining specific inclusion criteria.
**WP3:** Musculoskeletal system		
**Risk 3.1:**<br>Failure to receive ethics approval for obtaining muscle biopsies**.**		See Risk 2.1 above
**Risk 3.2:**<br>Failure to obtain sufficient muscle tissue during the biopsy procedure.		See Risk 2.2 above
**Risk 3.3:**<br>Inaccessibility of CT machine		There are several CT machines available within a 1 hr driving range of the Planica facility. In the past, we have transferred subjects in bedrest studies from a bedrest facility to the site of a measurement device. We have

		had the support of the Ministry of Defence Medical Services, which provided the medical staff, drivers and vehicles for the transfer of subjects. Post-bedrest CTs will be conducted in normoxia, thus there will be no need for hypoxic transfer of subjects. Moreover, a peripheral CT scanner that is portable and owned by one of the PIs (Rittweger) will be available to the study and would be able to cover data collection on the calf muscles.
**WP4:** Monitoring of stress and hypoxia sensitive immune and haematological changes		
**Risk 4.1:**<br>Failure to receive ethics approval for obtaining blood samples, and risks associated with withdrawal of blood samples.	**Low**	The collection of tissue specimens is compliant with the current applicable European laws and guidelines for human biomedical research (GCP: Guidelines of Good Clinical Practice). The investigators will have applied for approval of the study protocol by the Slovene National Ethics Committee, which has already granted many approvals for blood collection from healthy humans and patients in many trials including bed rest and hypoxia. The risk associated with the specific tasks (general risk assessment in WP1) solely results from the venous puncture to draw blood. However, when blood collection is performed according to the standard operating procedures, which include skin disinfection by alcohol pads prior to venous puncture and puncture by experienced personnel, no risk higher than with any other routine blood draw for health control purposes can be anticipated. The total blood volume taken per subject per time point is not more than 13 ml. The collections will be spread over almost one year.

**Risk 4.2:**<br>Failure to obtain urine and salive samples	**Low**	Urine, saliva and air sampling are non-invasive and do not require needles or any other potentially hazardous materials.
**Risk 4.3:**<br>Transportation of blood, urine and salive samples to respective laboratories.	**Low**	Much of the analysis will be conducted either at the Planica facility, or in clinical laboratories at the General Hospital in Jesenice (20 min. drive from Planica facility), or in University Clinical Centre Ljubljana (1 hr drive) or in the laboratories at the LMU or CU. The transfer of biological samples has been required in almost all previous studies at the Planica facility, thus this is now a standard routine procedure carried out by the research personnel or designated couriers.<br>In the past, sample have also been successfully transported to collaborating laboratories (ie. KTH, UniTs, UoN) for further analysis. The transportation has been conducted by either JSI drivers, international courier services, or by the research personnel. The procedures for transportation of biological samples within Europe are now standard, and minimal risks are involved.
**WP5:** Hypoxia and physical inactivity regulation of oxidative stress and thermogenesis		
**Risk 5.1:** Failure to receive ethics approval for stable isotopic precursors infusions	**Low**	Ethics approval for stable isotopic precursors infusions has been obtained previously for two bed rest studies.
**Risk 5.2:**<br>Failure to receive ethics approval for obtaining fat biopsies.	**Low**	Ethics approval for withdrawing fat biopsies has been obtained previously for a hypoxia confinement study.
**Risk 5.3:**<br>Failure to obtain sufficient muscle and adipose tissue during the biopsy procedures.	**Low**	The standard operating procedures utilised to date by individual partners (JSI, KTH, UoN) has provided sufficient tissue sample size for the

		required analyses.
**Risk 5.4:** Failure of transportation of blood, urine and saliva samples to respective laboratories.	**Low**	See Risk 4.3 above
**WP6:** Hypoxia-induced thermoregulatory dysfunction, sleep disorders, and ventilatory equivalent altitude		
**Risk 6.1:** Failure to obtain quality EEG recordings during sleep studies.	**Low**	We have secured the assistance of the Head of the Sleep Laboratory at the University Clinical Centre Ljubljana (Dr. Leja Dolenc), who has extensive research in EEG recordings.
**Risk 6.2:** Obtaining fundus photographs during the interventions.	**Low**	PlanHab coordinator has secured a grant from the Programme for European Cooperating States fund. The funding will be used to purchase equipment, such as a fundus camera. We have also secured the assistance of Dr. Jaki Mekjavic (Eye Clinic, University Clinical Centre Ljubljana), who has monitored subjects participating in previous hypoxic studies for signs of altitude retinopathy.
**Risk 6.3:** Functioning of water perfused suit to assess behavioural temperature regulation.	**Low**	Although a prototype device, it has been used successfully in many studies. One such study was awarded the best student presentation at the 2007 International Conference on Environmental Ergoomics.
**WP7:** Dissemination, IPR and Training		
**Risk 7.1:** Failure to implement measures for the dissemination of project results.	Low	PlanHab Coordinator has secured a Slovene Research Agency grant to conduct a short-term bedrest study. Thus, almost ALL the initiatives listed in section 3.2 Dissemination and/or exploitation of project results, and management of intellectual property, are already in progress to satisfy the Dissemination requirements of the short-term bedrest study..

**Risk 7.2:**<br>Failure to implement measures for the exploitation of project results.	**Low**	See comments for Risk 7.1 above.
**Risk 7.3:**<br>Failure to reach IPR agreement among partners.	**Low**	PlanHab partners have already indicated that they are in favour of an IPR agreement.
**WP8:** Management		
**Risk 8.1:**<br>Failure to provide deliverables.	**Low**	ALL partners have experience in conducting bedrest studies, and many have also collaborated on such studies, as well. All proposed methods have been used before. The possibility of not being able to complete a study and thus not being able to provide the stated deliverables is low.<br>Furthermore, the Project Management Committee will maintain a risk diary to alert partners in advance of any problems that might jeopardise the success of the project.
**Risk 8.2:**<br>Failure to meet budgetary guidelines.	**Low**	The PlanHan budget has been created based on previous bedrest and confinement studies. Financial status of the project will be monitored by a designated financial advisor.
**Risk 8.3:**<br>Failure to obtain ethics approval for the study.	**Low**	Ethics approval has previously been obtained for 5-week horizontal and 6° head-down tilt bedrest studies. Following ethics approval, a total of four (4) such studies have been performed by the coordinator and KTH in the past,

4. ETHICAL ISSUES

All the experiments will be performed at the Olympic Sport Centre Planica in Slovenia. Therefore, for the experiments performed in Slovenia, only the approval of a Slovene ethics committee is applicable. All Information for Subjects sheets must also be written in Slovene. The request for Ethics approval of the study will be submitted by the Coordinator (Mekjavic) who will also receive the formal approval from the National Ethics Committee, and will be responsible for all Ethics issues of the studies performed at the Olympic Sport Centre Planica. Although all investigators might have ethics approval for performing the studies in their respective institutes and countries, these approvals are not valid in Slovenia. All procedures and protocols must be approved by the Slovene National Medical Ethics Committee (NMEC). The protocol of the study will be evaluated by this committee (Chairman: Prof. dr. Jože Trontelj; Secretary: Mr. Tone Zakelj) at the Ministry of Health.

The National Medical Ethics Committee of the Ministry of Health of the Republic of Slovenia maintains a bilingual (Slovene/English) website (www.kme-nmec.si), where instructions for submitting applications to the Committee are itemised. The Instructions for the APPLICATION FOR ETHICS REVIEW are reproduced in Appendix 5. All the points listed in the Application form must be addressed in detail, and to the satisfaction of the National Medical Ethics Committee.

The NMEC meets on a monthly basis, and provides a response within 1 month. The dates of the scheduled meetings are posted on their website (www.kme-nmec.si). PlanHab coordinator will submit an application to the NMEC. The Chairman of the NMEC will be requested to provide a written response in English. The ruling of the NMEC will then be forwarded to the Project Officer.

The Coordinator (Mekjavic) has received approval from the Ministry of Health (Republic of Slovenia) National Medical Ethics Committee for a 10 day feasibility study, which contains all the procedures that will be performed in the PlanHab project. In addition, the Coordinator, together with Prof. Ola Eiken (KTH) have received approval from the Ministry of Health National Medical Ethics Committee for three 35-day bedrest studies, conducted previously. It should also be noted that many of the FP7 partners have acted as Chairmen, Secretaries, or Members of their institutional Ethics Review Committee, and are cognizant of the Declaration of Helsinki guidelines, as well as the document issued by the European Commission “Ethics for Researchers. Facilitating Research Excellence in FP7” by Eleonore Pauwels (2007).

Where applicable, copies of ethical/legal approvals by the competent local/regional/national Ethics Committees will be submitted to the REA prior to the commencement of the relevant part of the research . The progress of compliance with the above commitment will be described in the Periodic Reports/Final Report (under section 3.2.2. – Work progress and achievements during the period.

The issues raised by the European Commission DG RTD Ethics Committee reviewing the PlanHab proposal have been addressed in our submission to the Ministry of Health National Medical Ethics Committee. The manner in which these have been addressed is provided below.

Recruitment procedures

Subjects will be recruited through advertisements. Advertisements will be placed with all Students Services in Slovenia. Studies within the PlanHab project involve the investigation of the hormonal and thermoregulatory responses during the three interventions. Due to the menstrual cycle, there is a great degree of variability in these responses in adult female subjects. For this reason, studies should investigate these responses separately in these two populations (females and males). Each study should incorporate a sufficient number of subjects to ensure compliance with statistical requirements. The present research programme will investigate the effect of the three interventions on both genders. The study involving male subjects will be conducted within the framework of the PlanHab project. In parallel to this project, a submission has been made to the European Space Agency to conduct a similar investigation on female subjects, within the framework of the Plan for European Cooperating States (PECS). Thus, for the puproses of the current PlanHab project, only healthy adult male subjects will be recruited.

Selection of test subjects

Subjects responding to the advertisements will be initially screened during a telephone interview. They will be informed of the general nature of the study, and be explained the nature of the experiments that will be performed. Those that will continue to show interest in participating in the study will be invited for an interview. The interview will be conducted by a panel of participating scientists, who will explain the risks and hazards of the experimental procedures, and also emphasise the manner in which these risks will be minimised. The panel will comprise personnel that have experience in conducting such studies. They will also focus on the day-to-day logistics of the study, and answer any questions the subjects may have. Following these interviews a shortlist of subjects will be drawn. Following these selection criteria, subjects will be invited to spend 4 days at the Olympic Sport Centre, where the study will be carried out. During this week, subjects will participate in the baseline experiments. Performing baseline experiments approximately 1 month prior to the start of the study will ensure that the subjects understand the nature of all the experiments, and that they are able to complete the required tasks. This week will also allow the subjects to get to know one another, and to suggest room assignments to the staff. Namely, during the study subjects will be housed in the facility two per room. During this week, subjects will need to adhere to the basic guidelines of the study: no alcohol, no coffee, no smoking, regulated sleeping hours. Those subjects that will not be able to adhere to these simple rules will not be invited to continue their participation in the study.

Informed consent

Subjects will be requested to give their written consent to participating in the study. It will be emphasised that they can withdraw their participation in the study at any time without any prejudice.

Insurance

The bedrest studies will be performed in Slovenia, where health insurance is mandatory. All subjects will therefore have valid medical insurance. In addition, in the event that students will be participating in the studies, their participation will be mediated by the Students Services, in which case they will be provided with additional work-related insurance.

Compensations in the case of injury
As indicated above, all subjects will be insured against injury during the course of the study.

Subject compensation for participation in the study
The guidelines of the Ministry of Health (Republic of Slovenia) Ethics Review Committee state that payment received by subjects for participating in experiments should compensate them for their time only. The subjects will be paid a minimal hourly wage of €3 per hour. Since they will be requested to participate in three experimental campaigns, each lasting 28 days (including the pre- and post-intervention test days), we will therefore offer them a total of (28 days x 24 hours per day x €3 per hour x 3 experimental campaigns=) € 6048 for participating in all three campaigns. The subjects will receive this amount in three instalments over the course of the year.
In the event that the subjects discontinue their participation in the study, for whatever reason, they will receive monetary compensation for their participation up to, and including, the day of the termination of their involvement in the study.

Handling of incidental findings
Incidental findings will be grouped in two categories:
1) Log of incidents: a daily log of any unplanned incidents will be kept. These will be incidents that may compromise an experiment, unnecessarily increase the complexity of a procedure, or may be a hazard for the subjects or staff members. Any such incidents will be analysed immediately, and procedures implemented to ensure that they do not re-occur.
2) Incidental findings: all measurements obtained on a subject are confidential. In the event that during the course of an experiment observations are made relating to the health of the subject, the subject will be immediately informed. His continued participation will be subject to approval of the attending physician who will be aware of the incidental finding. An example of such a finding would be the identification of a disorder during the routine analysis of the blood samples. If the attending physician deems the disorder such that it does not place the subject in any danger by participating in the study, the subject will be allowed to continue. However, if the medical team are of the opinion that the subject's physician should be notified of this disorder, they will communicate this to the subject.

Psychological support for the research patients (if required)
Psychological support will be provided by a consulting psychiatrist. S/he will be a member of the medical team responsible for the subjects' well-being. S/he will also be a member of the team, which will interview the shortlisted candidates.

Discussion of experimental procedures
Prior to start of the experimental campaigns, all PlanHab partners will meet to review the total study design, as well as individual experimental procedures. The discussion will ensure that the study design is appropriate, such that:
- there is no overlap of individual experiments
- any potential crossover effect between the different experiments is minimised
- subjects have sufficient time between tests to recover
- the experimental schedule is not too fatiguing for the subjects
- personnel are available for performing the tests.

Continued review process
In past studies, we have provided the National Medical Ethics Committee with feedback regarding the progress of the study. In instances where additional guidance is required from the Ethics Committee during the course of the study, we will schedule an immediate meeting with the Secretary of the Ethics Committee, who will then bring the matter to the attention of the Chairman of the National Medical Ethics Committee. In the past, we have been able to meet with the Secretary of the Ethics Committee on the same day an issue was raised.

Data management
All experimental data that will be collected during the study will only be available to the researchers directly involved in the study. Individual data will be coded so that the identity of any given subject cannot be linked to the data unless access to the code key is provided (which will be kept in a safe room at the Jozef Stefan Institute). On request, subjects will be provided with results of experiments in which they served as a test subject, but not results of experiments performed by other subjects.
The Jozef Stefan Institute has a safe room available for data storage and meetings. This room has been certified by the Ministry of Defence (using NATO guidelines) as being appropriate for storage of sensitive/secret information. Information stored in this room pertains only to the experiments conducted at the Jozef Stefan Institute. Access to this room, as well as to the information contained in the room, is regulated by the JSI Security Officer.
The coded results will be added to the Data Archive currently being developed by the European Space Agency. It will not be possible to connect the information contained in the archive with individual subjects.

Data protection
Subjects‘ pariticipation in the study will be confidential. All data will archived with reference only to the subjects‘ number, and will not be linked to a subject’s name. A code will be kept in safe-keeping at JSI. This code will not be disclosed to anyone, other than the partners in the study. All will sign a confidentiality agreement, ensuring subject’s privacy.

All principal investigators will be requested to have only coded information regarding the subjects on their computers. Guidelines governing the handling of clinical patient information will also govern the handling of the information obtained from the volunteers. This includes the screening questionnaires and collected data. All un-coded data will be stored in the secure safe room at the Jozef Stefan Institute, which has been designed according to NATO guidelines for storage of sensitive information. This room is used by JSI researchers to store classified research data. Its use is controlled by the JSI Security Officer, and only individuals with appropriate clearance have access to this room. The Coordinator (Mekjavic) has successfully completed a Ministry of Defence sponsored and organised course on handling sensitive/secret data. The completion of this course was necessary for participation in NATO Human Factors in Medicine panels. The Coordinator therefore has experience and training in handling sensitive information. A certificate of completion of the course, as well as the level of security clearance received by the Coordinator is available on request. Also, a certificate regarding the security level of the safe room at JSI, where the data will be stored, is also available on request.

Level of intrusiveness of the tests as well as eventual risks

To obtain tissue specimens from the vastus lateralis muscle by means of the needle biopsies is a well-established (standard) technique that has been used for more than 4 decades in thousands of healthy subjects with very few reports of adverse events. To reduce any foreseeable risk of accidental blood vessel rupture or nerve puncture, an examination of the biopsy site will be performed with ultrasound imaging, identifying any of these structures. From this examination the best approach to the vastus lateralis muscle will be decided and marked on the skin.

The whole biopsy procedure from the initial administration of the anaesthetic to closing of the biopsy site will take approximately 10 - 15 minutes.

When the anaesthetic wears off, the participant may feel the biopsy site to be rather tender (for 1–2 days) and there may be some bruising, but in general the muscle biopsy is very well tolerated. The muscle itself recovers very rapidly and the biopsy will have no effect on muscle strength or day-to-day living. There is no contraindication against participants performing maximal muscle contractions the day after the biopsy. This procedure is common practice in our laboratory and no severe pain or inability to perform contractions is observed.

Participants need to keep the site dry for 2 - 3 days to allow the cut to heal and should avoid bathing where possible. After the biopsy, it is important to monitor the incision and to check that it is healing properly. In the unlikely event that it becomes red and inflamed the attending medical doctor in charge will be alerted and will keep the principal investigators informed. There are no indications to suspect that repeated biopsies will introduce any additional risk; the practise of repeated (multiple) biopsies is indeed very common and has been so for many decades.

The procedure for obtaining fat biopsies is similar to that for muscle biopsies.

The prolonged and repeated medical investigations may induce fatigue in the subjects. This will be taken into account in scheduling the investigations. Subjects will also complete questionnaires on a daily basis, which will document their mood and psychological status. They will be under constant supervision by medical staff, and will have regular discussions with the resident psychologist/psychiatrist.

The nature of the experimental campaigns, involving 21-day bed rests in normoxia or hypoxia, and the confinement in a hypoxic floor of the Olympic Sport Centre is certainly extraordinary. During the interview process the personnel will continuously emphasise the nature of the stress subjects may experience, and will also emphasise that they may terminate any experimental procedure at any time, and that they may also withdraw their participation from the study at any time.

Rehabilitation

Upon completion of each bedrest/confinement trial, the subjects will be requested to participate in a supervised rehabilitation programme. This programme will comprise prescribed daily exercise.

ETHICAL ISSUES TABLE

	Research on Human Embryo/Foetus	Yes	Page
*	Does the proposed research involve human Embryos?		
*	Does the proposed research involve human Foetal Tissues/ Cells?		
*	Does the proposed research involve human Embryonic Stem Cells (hESCs)?		
*	Does the proposed research on human Embryonic Stem Cells involve cells in culture?		
*	Does the proposed research on human Embryonic Stem Cells involve the derivation of cells from Embryos?		
	I confirm that none of the above issues apply to my proposal.	X	

	Research on Humans	Yes	Page
*	Does the proposed research involve children?		
*	Does the proposed research involve patients?		
*	Does the proposed research involve persons not able to give consent?		
*	Does the proposed research involve adult healthy volunteers?		
	Does the proposed research involve Human genetic material?		
	Does the proposed research involve Human biological samples?	X	
	Does the proposed research involve Human data collection?	X	
	I confirm that none of the above issues apply to my proposal.		

	Privacy	Yes	Page
*	Does the proposed research involve processing of genetic information or personal data (e.g. health, sexual lifestyle, ethnicity, political opinion, religious or philosophical conviction)?		
*	Does the proposed research involve tracking the location or observation of people?		
	I confirm that none of the above issues apply to my proposal.	X	

	Research on Animals	Yes	Page
	Does the proposed research involve research on Animals?		
	Are those animals transgenic small laboratory animals?		
	Are those animals transgenic farm animals?		
*	Are those animals non-human primates?		
	Are those animals cloned farm animals?		
	I confirm that none of the above issues apply to my proposal.	X	

	Research Involving Developing Countries	Yes	Page
	Does the proposed research involve the use of local resources (genetic, animal, plant, etc.)?		
	Is the proposed research of benefit to local communities (e.g. capacity building, access to healthcare, education)?	X	
	I confirm that none of the above issues apply to my proposal.		

	Dual Use	Yes	Page
	Research having direct military use.		
	Research having the potential for terrorist abuse.		
	I confirm that none of the above issues apply to my proposal.	X	

5. CONSIDERATION OF GENDER ASPECTS

Gender perspective is an integral part of the project's overall approach. The gender issues related to the present project will be discussed with regards to the Partners participating in the project, and the research project itself. The project offers equal opportunities between women and men and promotes the equality of women. During the proposed activities all partners will at all times account for the following:

- Women's participation in research, must be encouraged both as scientists/technologists and within the evaluation, consultation and implementation processes,
- Research must address women's needs, as much as men's needs,
- Research must be carried out to contribute to an enhanced understanding of gender issues.
- Gender balance will be the aim of all PlanHab partners.
- Measures will be implemented to help reconcile work and private life.
- Improving gender equality awareness will be addressed by PlanHab partners. The intention being to enhance female participation in research.

The partners have also discussed using both male and female subjects in the studies.

7. References

Agostini F, dalla Libera L, Rittweger J, Mazzucco S, Jurdana M, Guarnieri G, Mekjavic I, Pisot I, Gorza L, Narici M and Biolo G. Effects of inactivity on human muscle glutathione synthesis by a double tracer and single biopsy approach. *Journal of Physiology (London)* (2010).

Ahmad, R., Tripathi, A. K., Tripathi, P., Singh, S., Singh, R., & Singh, R. K. (2008). Malondialdehyde and protein carbonyl as biomarkers for oxidative stress and disease progression in patients with chronic myeloid leukemia. In Vivo 22, 525-528.

Allen DL, Bandstra ER, Harrison BC, Thorng S, Stodieck LS, Kostenuik PJ, Morony S, Lacey DL, Hammond TG, Leinwand LL, Argraves WS, Bateman TA, Barth JL. Effects of spaceflight on murine skeletal muscle gene expression. J Appl Physiol. 2009 Feb;106(2):582-95.

Altomare, E., Vendemiale, G., & Albano, O. (1988). Hepatic glutathione content in patients with alcoholic and non alcoholic liver diseases. Life Sci. 43, 991-998.

Andersen P, B Saltin. Maximal perfusion of skeletal muscle in man. *J Physiol* 366: 233-248, 1985.

Anderson, M. E. (1997). Glutathione and glutathione delivery compounds. Adv.Pharmacol. 38, 65-78.

Appell, H. J., Duarte, J. A., & Soares, J. M. (1997). Supplementation of vitamin E may attenuate skeletal muscle immobilization atrophy. Int.J.Sports Med. 18, 157-160.

Arnett TR. Acidosis, hypoxia and bone. *Arch Biochem Biophys* (2010); **503**: 103-109.

Aschenbrenner, V., Zak, R., Cutilletta, A. F., & Rabinowitz, M. (1971). Effect of hypoxia on degradation of mitochondrial components in rat cardiac muscle. Am.J.Physiol 221, 1418-1425.

Askew, E. W. (2002). Work at high altitude and oxidative stress: antioxidant nutrients. Toxicology 180, 107-119.

Baker M.A. & P.A. Ddoris. 1982(a). Effect of dehydration on hypothalamic control of evaporation in the cat. *J Physiol* 322: 457 - 468.

Baker, M.A. & P.A. Doris. 1982(b). Control of evaporative heat loss during changes in plasma osmolality. *J Physiol* 328: 535 - 545.

Baker M. A. B. 1984. Influence of dehydration on thermoregulation in panting mammals. In: Thermal Physiology. Ed.: J.R. Hales, New York: Raven Press, pp. 407 - 412.

Barreiro, E., de la, P. B., Minguella, J., Corominas, J. M., Serrano, S., Hussain, S. N., & Gea, J. (2005). Oxidative stress and respiratory muscle dysfunction in severe chronic obstructive pulmonary disease. Am.J.Respir.Crit Care Med. 171, 1116-1124.

Barreiro, E., Schols, A. M., Polkey, M. I., Galdiz, J. B., Gosker, H. R., Swallow, E. B., Coronell, C., & Gea, J. (2008). Cytokine profile in quadriceps muscles of patients with severe COPD. Thorax 63, 100-107.

Bassett Frey MA, JB Charles, DE Houston. Weightlessness and orthostatic stress. In: Circulatory responses to the upright posture Ed: JJ Smith. CRC Press, pg 65-20. 1990.

Beckman, K. B. & Ames, B. N. (1998). The free radical theory of aging matures. Physiol Rev. 78, 547-581.

Betters, J. L., Criswell, D. S., Shanely, R. A., Van Gammeren, D., Falk, D., DeRuisseau, K. C., Deering, M., Yimlamai, T., & Powers, S. K. (2004). Trolox attenuates mechanical ventilation-induced diaphragmatic dysfunction and proteolysis. Am.J.Respir.Crit Care Med. 170, 1179-1184.

Berg HE, O Eiken, L Miklavcic, IB Mekjavic. Hip, thigh and calf muscle atrophy and bone loss after 5-week bedrest inactivity. Eur J Appl Physiol99:283-289. 2007.

Biolo, G., Agostini, F., Simunic, B., Sturma, M., Torelli, L., Preiser, J. C., Deby-Dupont, G., Magni, P., Strollo, F., di Prampero, P., Guarnieri, G., Mekjavic, I. B., Pisot, R., & Narici, M. V. (2008). Positive energy balance is associated with accelerated muscle atrophy and increased erythrocyte glutathione turnover during 5 wk of bed rest. Am.J.Clin.Nutr. 88, 950-958.

Blottner D, Salanova M, Puttmann B, Schiffl G, Felsenberg D, Buehring B and Rittweger J. Human skeletal muscle structure and function preserved by vibration muscle exercise following 55 days of bed rest. *Eur J Appl Physiol* (2006); **97**: 261-271.

Blottner D, Rittweger J, Felsenberg D, Cerretelli P and Gelfi C. Long term bed rest with and without vibration exercise countermeasures: Effects on human muscle protein dysregulation. *Proteomics* (2010).

Blüher M., Wilson-Fritch L., Leszyk J., Laustsen P.G., Corvera S. and Kahn C.R. (2004). Role of insulin action and cell size on protein expression patterns in adipocytes. *The J Biol Chem 279:31902-31909*

Bodkin, D.K., Escalera, P, Bocam K.J. A human Lunar surface base and infrastructure solution. American Institute of Aeronautics and Astronautics 2006-7336. 17 pp.

Bodine, S. C., Latres, E., Baumhueter, S., Lai, V. K., Nunez, L., Clarke, B. A., Poueymirou, W. T., Panaro, F. J., Na, E., Dharmarajan, K., Pan, Z. Q., Valenzuela, D. M., DeChiara, T. M., Stitt, T. N., Yancopoulos, G. D., & Glass, D. J. (2001). Identification of ubiquitin ligases required for skeletal muscle atrophy. Science 294, 1704-1708.

Bogert, LWJ, and Van Lieshout, JJ. (2005). Non invasive pulsatile arterial pressure and stroke volume changes from the human finger. Experimental Physiology, 90 (4): 437-446.

Buckey, JC, Andrew Gaffney, JrF, Lane, LD, Levine, BD, Watenpaugh, DE, Wright, SJ, Yancy, CW, Meyer, DM, and Blomqvist, CG. (1996). Central venous pressure in space. Journal of Applied Physiology, 81 (1): 19-25.

Bota, D. A. & Davies, K. J. (2002). Lon protease preferentially degrades oxidized mitochondrial aconitase by an ATP-stimulated mechanism. Nat.Cell Biol. 4, 674-680.

Bougssuges A, F Molenat, H Burnet et al. Operation Everest III (Comex 97): modifications of cardiac function secondary to altitude-induced hypoxia. Am J Respir Crit Care Med 161: 264-270. 2000.

Boveris, A. & Chance, B. (1973). The mitochondrial generation of hydrogen peroxide. General properties and effect of hyperbaric oxygen. Biochem.J. 134, 707-716.

Cadenas, E., Brigelius, R., & Sies, H. (1983). Paraquat-induced chemiluminescence of microsomal fractions. Biochem.Pharmacol. 32, 147-150.

Calbet JAL, C Lundby. Air to muscle O_2 delivery during exercise at altitude. *High Alt Med Biol* 10: 123-134, 2009.

Candas V., J.P. Libert, G. Brandenberger, J.C. Sagot, C. Amoros & J.M. Kahn. (1986). Hydration during exercise. Effects on thermal and cardiovascular function. *Eur J Appl Physiol* 55: 113 - 122.

Cerretelli P and Hoppeler H. Morphologic and metabolic response to chronic hypoxia: the muscle system. In: *Handbook of Physiology*, (eds. Fregly, M. J. and Blatteis, C. M.) (1996); vol. 2, pp. 1155-1181. Oxford University Press, Oxford.

Cerretelli P, Marzorati M and Marconi C. Muscle bioenergetics and metabolic control at altitude. *High Alt Med Biol* (2009); **10**: 165-174.

Chao, W. H., Askew, E. W., Roberts, D. E., Wood, S. M., & Perkins, J. B. (1999). Oxidative stress in humans during work at moderate altitude. J.Nutr. 129, 2009-2012.

Chowdhury H.H., Grilc S. and Zorec R. (2005). Insulin induces a rapid increase in membrane area in single rat adipocytes. *Adipocytes 2:131-138*

Convertino VA. Exercise and adaptation to microgravity environments. In: Handbook of Physiology, Sect 4: Environmental Physiology Volume II (Eds: Fregly & Blatteis). Oxford Univ Press. Pg 815-843. 1996.

Convertino V.A., Doerr, D.F., Eckberg, D.L., Fritsch, J.M., Vernokos-Danellis, J. Head-down bedrestr impairs vagal baroreflex responses and provokes orthostatic hypotension. J Appl Physiol 68: 1458-1464, 1990.

Crandall C.G., J.M. Johnson, V.A Convertino, P.B. Raven & K.A. Engelke. 1994. Altered thermoregulatory responses after 15 days of head down tilt. *J Appl Physiol* 77: 1863–1867.

Crawford IA. Towards an integrated scientific and social case for human space exploration. Earth, Moon, and Planets 94(3-4): 245-266. 2004

Dalle-Donne, I., Giustarini, D., Colombo, R., Rossi, R., & Milzani, A. (2003). Protein carbonylation in human diseases. Trends Mol.Med. 9, 169-176.

Desaphy JF, Pierno S, Liantonio A, Giannuzzi V, Digennaro C, Dinardo MM, Camerino GM, Ricciuti P, Brocca L, Pellegrino MA, Bottinelli R and Camerino DC. Antioxidant treatment of hindlimb-unloaded mouse counteracts fiber type transition but not atrophy of disused muscles. *Pharmacol Res* (2010); **61**: 553-563.

Deveci D, Marshall JM and Egginton S. Relationship between capillary angiogenesis, fiber type, and fiber size in chronic systemic hypoxia. *Am J Physiol Heart Circ Physiol* (2001); **281**: H241-252.

di Prampero PE. Factors limiting maximal performance in humans. *Eur J Appl Physiol* 90: 420-429, 2003.

Drost, E. M., Skwarski, K. M., Sauleda, J., Soler, N., Roca, J., Agusti, A., & MacNee, W. (2005). Oxidative stress and airway inflammation in severe exacerbations of COPD. Thorax 60, 293-300.

Dukan, S., Farewell, A., Ballesteros, M., Taddei, F., Radman, M., & Nystrom, T. (2000). Protein oxidation in response to increased transcriptional or translational errors. Proc.Natl.Acad.Sci.U.S.A 97, 5746-5749.

Eiken O, CJ Sundberg, M Esbjörnsson, A Nygren, L Kaijser. Effects of ischaemic training on force development and fibre-type composition in human skeletal muscle Clin Physiol 11: 41-49, 1991.

Eiken O, Mekjavic, I.B. The Valdoltra Bedrest Study; effects of 35 days of horizontal bedrest on the function of peripheral blood vessels, the thermoregulatory system and the function and structure of the musculoskeletal system. FOI report: FOI-R—0748—SE. 2002. pp 60.

Eiken O, Kölegård R, Mekjavic IB. Pressure-distension relationshp in arteries and arterioles in response to 5 wk of horizontal bedrest. Am J Physiol Heart Circ Physiol. 295:H1296-H1302, 2008.

Ekblom B., C.J. Greenleaf, J.E. Greenleaf & L. Hermansen. 1970. Temperature regulation during exercise dehydration in man. *Acta Physiologica Scandinavica* 79: 475 - 483.

Elliott, S. (2008). Erythropoiesis-stimulating agents and other methods to enhance oxygen transport. British Journal of Pharmacology, 154 (3): 529-541.

Erslev, AJ, Caro, J, Birgegard, G, Silver, R, and Miller, O. (1980). The biogenesis of erythropoietin. Experimental Hematology, 8S (8): 1-13.

ESA. Aurora Programme: Executive Summary, 2003.

Faucher M, Guillot C, Marqueste T, Kipson N, Mayet-Sornay MH, Desplanches D, Jammes Y and Badier M. Matched adaptations of electrophysiological, physiological, and histological properties of skeletal muscles in response to chronic hypoxia. *Pflugers Arch* (2005); **450**: 45-52.

Fehr M., Lanolde S., Lager I., Wolff M.W. and Frommer W.B. (2003). In vivo imaging of dynamics of glucose uptake in the cytosil of COS-7 cells by fluorescent nanosensors. *J Biol Chem 278:19127-33*

Fitts RH, Trappe SW, Costill DL, Gallagher PM, Creer AC, Colloton PA, Peters JR, Romatowski JG, Bain JL and Riley DA. Prolonged Space Flight-Induced Alterations in the Structure and Function of Human Skeletal Muscle Fibres. *J Physiol* (2010).

Flueck M. Plasticity of the muscle proteome to exercise at altitude. *High Alt Med Biol* 10: 183-193, 2009.

Foldager, N, Andersen, TAE, Jessen, FB, Ellegaard, P, Stadeager, R, Videbaek, R, and Norsk, P. Central venous pressure in humans during microgravity. Journal of Applied Physiology, 81 (1): 408-412.

Fortney S.M., Scneider V.S., Greenleaf, J.E. The physiology of bedrestr. In: Handbook of physiology, Sect. IV, Environmental Physiology, Vol. II (Eds: Fregley & Blatteis) Oxford University Press, 1996. pp 889-939.

Fortney S.M., E.R. Nadel, C.B. Wenger & J.R. Bove. 1981. Effect of blood volume on sweating rate and body fluids in exercising humans. *J Appl Physiol* 51:1594 -1600.

Fortney S.M. 1987. Thermoregulatory adaptations to inactivity. In; Adaptive Physiology to Stressful Environments. Eds.: S. Samueloff & M.K. Yousef. CRC Press Inc.: Boca Raton, pp. 75 - 83.

Frings-Meuthen P, Baecker N and Heer M. Low-grade metabolic acidosis may be the cause of sodium chloride-induced exaggerated bone resorption. *J Bone Miner Res* (2008); **23**: 517-524.

Ghosh, C., Dick, R. M., & Ali, S. F. (1993). Iron/ascorbate-induced lipid peroxidation changes membrane fluidity and muscarinic cholinergic receptor binding in rat frontal cortex. Neurochem.Int. 23, 479-484.

Goll, D. E., Thompson, V. F., Li, H., Wei, W., & Cong, J. (2003). The calpain system. Physiol Rev. 83, 731-801.

Golja, P, and Mekjavic, IB. (2002). The effect of hypoxia on skin blood flow in humans. *Med Razgl*, 41:169-171.

Golja, P, Eiken, O, Rodman, S, Sirok, B, and Mekjavic, IB. (2002). Core temperature circdian rhythm during 35 days of horizontal bed rest. *Journal of Gravitational Physiology*, 9 (1): P187-188.

Golja, P, and Mekjavic, IB. (2003). Effect of hypoxia on preferred hand temperature. *Aviation Space and Environmental Medicine*, 74 (5): 522-526.

Gopal AS, MJ Schnellbacher, ZQ Shen et al. Freehand three-dimensional Echocardiography for determination of left ventricular volume and mass in patients with abnormal ventricles: Comparison with magnetic resonance imaging. J Am Soc Echcardiography 10: 853-861, 1997.

Gore, CJ, Rodriguez, FA, Truijens, MJ, Townsend, NE, Stray-Gundersen, and Levine, BD. (2006). Increased serum erythropoietin but not red cell production after 4 wk of intermittent hypobaric hypoxia (4,000 – 5,000 m). Journal of Applied Physiology, 101 (5): 1386-1393.

Grassi B, M Marzorati, F Lanfranconi, A Ferri, M Longaretti, A Stucchi, P Vago, C Marconi, L Morandi. Impaired oxygen extraction in metabolic myopathies: detection and quantification by near-infrared spectroscopy. *Muscle Nerve* 35: 510-520, 2007.

Green HJ, Sutton JR, Cymerman A, Young PM and Houston CS. Operation Everest II: adaptations in human skeletal muscle. *J Appl Physiol* (1989); **66**: 2454-2461.

Greenleaf J.E. & J.D. Reese. 1980. Exercise thermoregulation after 14 days of bed rest. *J Appl Physiol* 48: 72-78.

Greilberger, J., Koidl, C., Greilberger, M., Lamprecht, M., Schroecksnadel, K., Leblhuber, F., Fuchs, D., & Oettl, K. (2008). Malondialdehyde, carbonyl proteins and albumin-disulphide as useful oxidative markers in mild cognitive impairment and Alzheimer's disease. Free Radic.Res. 42, 633-638.

Grucza R., J.L. Lecroart, G. Carette, J.J. Hauser & Y. Houdas. 1987. Effect of voluntary dehydration on thermoregulatory responses to heat in men and women. *Eur J Appl Physiol* 56:317 - 322.

Grune, T. & Davies, K. J. (2003). The proteasomal system and HNE-modified proteins. Mol.Aspects Med. 24, 195-204.

Grune, T., Merker, K., Sandig, G., & Davies, K. J. (2003). Selective degradation of oxidatively modified protein substrates by the proteasome. Biochem.Biophys.Res.Commun. 305, 709-718.

Guenette JA, I Vogiatzis, S Zakynthinos et al. Human respiratory muscle blood flow measured by near-infrared spectroscopy and indocyanine green. J Appl Physiol 104, 1202-1210. 2008.

Gunga, HC, Kirsch, KA, Roecker, L, Kohlberg, E, Tiedemann, J, Steinach, M, and Schobersberger, W. (2007). Erythropoietin regulations in humans under different environmental and experimental conditions. Respiratory Physiology and Neurobiology, 158: 287-297.

Gunga, HC, Kirsch, K, Baartz, F, Maillet, A, Gharib, C, Nalishiti, W, Rich, I, and Rocker, L. (1996). Erythropoietin under real and simulated microgravity conditions in human. Journal of Applied Physiology, 81 (2): 761-773.

Harms, MPM, Wesseling, KH, Pott, F, Jenstrup, M, Van Goudoever, J, Secher, NH, and Van Lieshout. (1999). Continuous stroke volume monitoring by modelling flow from non-invasive measurement of arterial pressure in humans under orthostatic stress. Clinical Sciences, 97: 291-301.

Heinicke, K, Heinicke, I, Schmidt, W, and Wolfarth, B. (2005). A three-week traditional altitude training increases hemoglobin mass and red cell volume in elite biathlon athletes. International Journal of Sports Medicine, 26 (5): 350-355.

Hirata K, T. Ban, Y Jinnouchi, S Kubo. Echocardiographic assessment of left ventricular function and motion at high altitude in normal subjects. Am J Cardiol 68: 1692-1697. 1991

Hufenbach, B., Seibert, G. Human space flight: achievements, benefits and future opportunities from a European perspective. Earth, Moon, and Planets 94: 185-212, 2005.

Hoppeler H and Desplanches D. Muscle structural modifications in hypoxia. *Int J Sports Med* (1992); **13 Suppl 1**: S166-168.

Hoppeler H and Vogt M. Muscle tissue adaptations to hypoxia. *J Exp Biol* (2001); **204**: 3133-3139.

Hosogai N., Fukuhara A., Oshima K., Miyata Y., Tanaka S., Segawa K., Furukawa S., Tochino Y., Komuro R., Matsuda M and Shimomura I. (2007). Adipose tissue hypoxia in obesity and its impact on adipocytokine dysregulation. *Diab 56: 901–911*

Huez S, K. Retailleau, P. Unger et al. Right and left ventricular adaptation to hypoxia: a Doppler imaging study. Am J Physiol, Heart Circ Physiol 289: H1391-H1398. 2005.

Hunter, R. B., Mitchell-Felton, H., Essig, D. A., & Kandarian, S. C. (2001). Expression of endoplasmic reticulum stress proteins during skeletal muscle disuse atrophy. Am.J.Physiol Cell Physiol 281, C1285-C1290.

Janakus J. Internal pressure in a lunar inflatable structure. In: Engineering, construction, and operations in space-III: Space '92; Proceedings of the 3rd International Conference, Denver, CO, May 31 – June 4, 1992.Vol. 2 (A93-41976 12-12), pp. 2360-2366.

Jeanrenaud B. (1978). Hyperinsulinaemia in obesity syndromes: its metabolic consequences and possible etiology. *Metab. 27:1881-1892*

Jefferson, J. A., Simoni, J., Escudero, E., Hurtado, M. E., Swenson, E. R., Wesson, D. E., Schreiner, G. F., Schoene, R. B., Johnson, R. J., & Hurtado, A. (2004). Increased oxidative stress following acute and chronic high altitude exposure. High Alt.Med.Biol. 5, 61-69.

Jelkmann, W. (1992). Erythropoietin: structure, control of production, and function. Physiological Reviews, 72, 449-489.

Ji, L. L. (1995). Exercise and oxidative stress: role of the cellular antioxidant systems. Exerc.Sport Sci.Rev. 23, 135-166.

Joanny, P., Steinberg, J., Robach, P., Richalet, J. P., Gortan, C., Gardette, B., & Jammes, Y. (2001). Operation Everest III (Comex'97): the effect of simulated sever hypobaric hypoxia on lipid peroxidation and antioxidant defence systems in human blood at rest and after maximal exercise. Resuscitation 49, 307-314.

Jones, D. P. & Kennedy, F. G. (1982). Intracellular oxygen supply during hypoxia. Am.J.Physiol 243, C247-C253.

Jones, D. P. & Mason, H. S. (1978). Metabolic hypoxia: accumulation of tyrosine metabolites in hepatocytes at low pO2. Biochem.Biophys.Res.Commun. 80, 477-483.

Jones D, Round J and de Haan A. *Skeletal muscle from molecules to movement* (2004). Churchill Livingstone, London.

Kakurin LI, Lobachik VI, Mikhailov VM and Senkevich YA. Antiorthostatic hypokinesia as a method of weightlessness simulation. *Aviat Space Environ Med* (1976); **47**: 1083.

Kano M, Kitano T, Ikemoto M, Hirasaka K, Asanoma Y, Ogawa T, Takeda S, Nonaka I, Adams GR, Baldwin KM, Oarada M, Kishi K, Nikawa T. Isolation and characterization of a novel gene sfig in rat skeletal muscle up-regulated by spaceflight (STS-90). J Med Invest. 2003 Feb;50(1-2):39-47.

Karimova, A. & Pinsky, D. J. (2001). The endothelial response to oxygen deprivation: biology and clinical implications. Intensive Care Med. 27, 19-31.

Kayser B, Hoppeler H, Claassen H and Cerretelli P. Muscle structure and performance capacity of Himalayan Sherpas. *J Appl Physiol* (1991); **70**: 1938-1942.

Khaw AV, RS von Bardeleben, C Strasser et al. Direct measurement of left ventricular outflow tract by transthoracic real-time 3D echocardiography increases accuracy in assessment of aortic valve stenosis. Int J Cardiol 136: 64-71, 2009.

Kirkham, P. & Rahman, I. (2006). Oxidative stress in asthma and COPD: antioxidants as a therapeutic strategy. Pharmacol.Ther. 111, 476-494.

Kölegård R, IB Mekjavic, O Eiken. Increased distensibbility in dependent veins following prolonged bedrest. Eur J Appl Physiol 106: 547-554. 2009.

Kondo, H., Miura, M., Kodama, J., Ahmed, S. M., & Itokawa, Y. (1992). Role of iron in oxidative stress in skeletal muscle atrophied by immobilization. Pflugers Arch. 421, 295-297.

Kondo, H., Nakagaki, I., Sasaki, S., Hori, S., & Itokawa, Y. (1993). Mechanism of oxidative stress in skeletal muscle atrophied by immobilization. Am.J.Physiol 265, E839-E844.

Kondo, H., Nishino, K., & Itokawa, Y. (1994). Hydroxyl radical generation in skeletal muscle atrophied by immobilization. FEBS Lett. 349, 169-172.

Kounalakis, SN, Keramidas, ME, Nassis, GP, and Geladas, ND. (2008). The role of muscle pump in the development of cardiovascular drift. European Journal of Applied Physiology, 103: 99-107.

Kourie, J. I. (1998). Interaction of reactive oxygen species with ion transport mechanisms. Am.J.Physiol 275, C1-24.

Krauchi, K, Cajochen, C, Werth, E, and Wirz-Justice, A. (1999). Warm feet promote the rapid onset of sleep. *Nature*, 401: 36-37.

Krauchi, K, and Wirz-Justice, A. (1994). Circadian rhythm of heat production, heart rate, and skin and core temperatureunder unmasking conditions in men. *American Journal of Physiology*, 267: R819-R829.

Krauchi, K, Cajochen, C, Werth, E, and Wirz-Justice, A. (2000). Functional link between distal vasodilation and sleep-onset latency? *American Journal of Physiology*, 278: R741-R748.

Kretzschmar, M., Muller, D., Hubscher, J., Marin, E., & Klinger, W. (1991). Influence of aging, training and acute physical exercise on plasma glutathione and lipid peroxides in man. Int.J.Sports Med. 12, 218-222.

Laufs, U., Wassmann, S., Czech, T., Munzel, T., Eisenhauer, M., Bohm, M., & Nickenig, G. (2005). Physical inactivity increases oxidative stress, endothelial dysfunction, and atherosclerosis. Arterioscler.Thromb.Vasc.Biol. 25, 809-814.

Lawler, J. M., Song, W., & Demaree, S. R. (2003). Hindlimb unloading increases oxidative stress and disrupts antioxidant capacity in skeletal muscle. Free Radic.Biol.Med. 35, 9-16.

Lawler, J. M., Song, W., & Kwak, H. B. (2006). Differential response of heat shock proteins to hindlimb unloading and reloading in the soleus. Muscle Nerve 33, 200-207.

Leach, CS, and Johnson, PC. (1984). Influence of space flight on erythrokinetics in man. Sciences, 225: 216-218.

LeBlanc A, Lin C, Shackelford L, Sinitsyn V, Evans H, Belichenko O, Schenkman B, Kozlovskaya I, Oganov V, Bakulin A, Hedrick T and Feeback D. Muscle volume, MRI relaxation times (T2), and body composition after spaceflight. *J Appl Physiol* (2000); **89**: 2158.

LeBlanc A, Schneider VS, Evans HJ, Pientok C, Rowe R and Spector E. Regional changes in muscle mass following 17 weeks of bed rest. *J Appl Physiol* (1992); **73** 2172.

LeBlanc AD, Schneider VS, Evans HJ, Engelbretson DA and Krebs JM. Bone mineral loss and recovery after 17 weeks of bed rest. *J Bone Miner Res* (1990); **5**: 843-850.

Lee S.M.C., W.J. Williams & S.M. Schneider. 2002. Role of skin blood flow and sweating rate in exercise thermoregulation after bed rest. *J Appl Physiol* 92: 2026-2034.

Leeuwenburgh, C. & Ji, L. L. (1995). Glutathione depletion in rested and exercised mice: biochemical consequence and adaptation. Arch.Biochem.Biophys. 316, 941-949.

Levine BD, JH Zuckerman, JA Pawelczyk. Cardiac atophy after bed-rest deconditioning: a nonhumoral mechanism for orthostatic intolerance. Circulation 96: 517-525, 1997.

Li, Y. P., Chen, Y., Li, A. S., & Reid, M. B. (2003). Hydrogen peroxide stimulates ubiquitin-conjugating activity and expression of genes for specific E2 and E3 proteins in skeletal muscle myotubes. Am.J.Physiol Cell Physiol 285, C806-C812.

McCarthy JJ. MicroRNA-206: the skeletal muscle-specific myomiR. Biochim Biophys Acta. 2008 Nov;1779(11):682-91. Epub 2008 Mar 12. Review. PubMed PMID: 18381085

McCarthy JJ, Esser KA, Peterson CA, Dupont-Versteegden EE. Evidence of MyomiR network regulation of beta-myosin heavy chain gene expression during skeletal muscle atrophy. Physiol Genomics. 2009 Nov 6;39(3):219-26. Epub 2009 Aug 18.

MacDougall JD, Green HJ, Sutton JR, Coates G, Cymerman A, Young P and Houston CS. Operation Everest II: structural adaptations in skeletal muscle in response to extreme simulated altitude. *Acta Physiol Scand* (1991); **142**: 421-427.

Manouras A, K Shahgaldi, R Winter et al. Measurment of left ventricular myocardial longitudinal systolic displacement using spectral and colour Doppler: time for a reassessment?. Cardiovascular Ultrasound 7: 1-12. 2009.

Mekjavic IB, O Eiken. Simulation of planetary habitats: a feasibility study. Abstract. In proceedings from “Life in Space for Life on Earth Conference, Trieste, Italy. 2010.

Mekjavic, IB, Kounalakis SN, Keramidas ME, Amon M, Debevec T, Simunic B, Pisot R, and Eiken O. (2009). Evaluation of hypoxic training protocols. Aviation Space and Environmental Medicine, 80 (3): 289.

Mekjavic, I.B., C.J. Sundberg & D. Linnarsson. 1991. Core temperature „null zone". J. Appl. Physiol. 71: 1289-1295.

Mekjavic IB, Golja P, Tipton MJ, Eiken O. Human temperature regulation during exercise and immersion after 35 days of horizontal bed-rest and recovery. Eur J Appl Physiol. 95:163-171. 2005.

Mekjavic I.B., Eiken O. (2001). Valdoltra Bedrest Study. Proposal submitted to the National Committee for Medical Ethics at the Ministry of Health of the Republic of Slovenia.

Mekjavic I.B., Eiken O., Pisot R. (2006). Valdoltra Bedrest Study 2006. Proposal submitted to, and approved by the National Committee for Medical Ethics at the Ministry of Health of the Republic of Slovenia.

Mekjavic I.B., Eiken O., Pisot R. (2007). Valdoltra Bedrest Study 2007. Proposal submitted to, and approved by the National Committee for Medical Ethics at the Ministry of Health of the Republic of Slovenia.

Mekjavic I.B., Eiken O., Pisot R. (2008). Valdoltra Bedrest Study 2008. Proposal submitted to, and approved by the National Committee for Medical Ethics at the Ministry of Health of the Republic of Slovenia.

Mekjavic I.B. (2008). Hypoxic training. Final Report to the Slovene Research Agency, and to the Ministry of Defence of the Republic of Slovenia.

Mendell WW. Meditation on the new space vision: The moon as a stepping stone mars. Acta Austronautica 57: 676-683. 2005.

Moriggi M, Vasso M, Fania C, Capitanio D, Bonifacio G, Salanova

Mendell, W.W. Meditation on the new space vision: The moon as a stepping stone to Mars. Acta Austronautica 57: 676-683. 2005.

Michelet, F., Gueguen, R., Leroy, P., Wellman, M., Nicolas, A., & Siest, G. (1995). Blood and plasma glutathione measured in healthy subjects by HPLC: relation to sex, aging, biological variables, and life habits. Clin.Chem. 41, 1509-1517.

Milledge J.S. (1972). Arterial oxygen desaturation and intestinal absorption of xylose. Brit Med J 2: 557-558.

Morrison, J. A., Jacobsen, D. W., Sprecher, D. L., Robinson, K., Khoury, P., & Daniels, S. R. (1999). Serum glutathione in adolescent males predicts parental coronary heart disease. Circulation 100, 2244-2247.

Motterlini, R., Foresti, R., Bassi, R., & Green, C. J. (2000). Curcumin, an antioxidant and anti-inflammatory agent, induces heme oxygenase-1 and protects endothelial cells against oxidative stress. Free Radic.Biol.Med. 28, 1303-1312.

Neher E. and Marty A. (1982). Discrete changes of cell membrane capacitance observed under conditions of echanced secretion in bovine adrenal chromaffin cells. *Proceedings of the National Academy of Sciences of the Unated States of America 79:6712-6716*

NASA, The Vision for Space Exploration, NP-2004-01-334-HQ, NASA Publication, Washington DC, 2004, 22 pp.

Naeije R. Pulmonary vascular function. In: Pulmonary Circulation: Diseases and Treatment (eds: Peacock & Rubin) London: Arnold. 2004.

Nikawa T, Ishidoh K, Hirasaka K, Ishihara I, Ikemoto M, Kano M, Kominami E, Nonaka I, Ogawa T, Adams GR, Baldwin KM, Yasui N, Kishi K, Takeda S. Skeletal muscle gene expression in space-flown rats. FASEB J. 2004 Mar;18(3):522-4. Epub 2004 Jan 8. PubMed PMID: 14715702.

Ortenblad, N., Madsen, K., & Djurhuus, M. S. (1997). Antioxidant status and lipid peroxidation after short-term maximal exercise in trained and untrained humans. Am.J.Physiol 272, R1258-R1263.

Pappas, KD, Gouva, CD, Katopodis, KP, Nikolopoulos, PM, Korantzopoulos, PG, Michalis, LK, Goudevenos, JA, and Siamopoulos, KC. (2008). Correction of anemia with erythropoietin in chronic kidney disease (stage 3 or 4): effects on cardiac performance. Cardiovascular Drugs and Therapy, 22 (1): 37-44.

Pastore, A., Federici, G., Bertini, E., & Piemonte, F. (2003). Analysis of glutathione: implication in redox and detoxification. Clin.Chim.Acta 333, 19-39.

Penaz, J. (1973). Photoelectric measurement of blood pressure, volume and flow in the finger. Digest of the International Conference on Medicine and Biological Engineering, Dresden, p. 104.

Platts SH, DS Martin, MB Stenger et al. Cardiovascular adaptations to long-duration head-down bed rest. Aviat Space Environ Med. 80 (Suppl 5) A29-A36. 2009.

Porcelli S, M Marzorati, F Lanfranconi, P Vago, R Pišot, B Grassi. Role of skeletal muscle impairment and brain oxygenation in limiting oxidative metabolism during exercise after bed rest. *J Appl Physiol* 109: 101-111, 2010.

Powers, S. K., Kavazis, A. N., & DeRuisseau, K. C. (2005). Mechanisms of disuse muscle atrophy: role of oxidative stress. Am.J.Physiol Regul.Integr.Comp Physiol 288, R337-R344.

Powers, S. K., Kavazis, A. N., & McClung, J. M. (2007). Oxidative stress and disuse muscle atrophy. J.Appl.Physiol 102, 2389-2397.

Primeau, A. J., Adhihetty, P. J., & Hood, D. A. (2002). Apoptosis in heart and skeletal muscle. Can.J.Appl.Physiol 27, 349-395.

Rabol R, S Larsen, MV Hoiberg et al. Regional autonomic differences in skeletal muscle mitochondrial respiration in type 2 diabetes and obesity. *J Clin Endocrinol Metab* 95: 857-863, 2010.

Rangwala S.M. and Lazar M.A. (2004). Peroxisome proliferator-activated receptor γ in diabetes and metabolism. *Trends Pharm Sci 25:331-336*

Rausch M.E., Weisberg S., Vardhana P and Tortoriello D.V. (2008). Obesity in C57BL/6J mice is characterized by adipose tissue hypoxia and cytotoxic T-cell infiltration. *Int J Obes (Lond) 32: 451–463*

Regazzetti C., Peraldi P., Gremeaux T., Najem-Lendom R., Ben-Sahra I., Cormont M., Bost F., Le Marchand-Brustel Y., Tanti J.F and Giorgetti-Peraldi S. (2009). Hypoxia decreases insulin signaling pathways in adipocytes. *Diab 58:95-103*

Rittweger J, B Simunic, G Bilancio et al. Bone loss in the lower leg during 35 days of bed rest is predominantly in the cortical compartment. Bone. 44:612-618. 2009.

Rogelj B, Giese KP. Expression and function of brain specific small RNAs. Rev Neurosci. 2004;15(3):185-98. Review.

Rogelj B, Hartmann CE, Yeo CH, Hunt SP, Giese KP. Contextual fear conditioning regulates the expression of brain-specific small nucleolar RNAs in hippocampus. Eur J Neurosci. 2003 Dec;18(11):3089-96.

Roth, E., Manhart, N., & Wessner, B. (2004). Assessing the antioxidative status in critically ill patients. Curr.Opin.Clin.Nutr.Metab Care 7, 161-168.

Rubinstein E.H. & D. Sessler. 1990. Skin-surface temperature gradients correlate with fingertip blood flow in humans. Anesthesiology 73: 541-545.

Salvadego D, S Lazzer, C Busti et al. Gas Exchange kinetics in obese adolescents. Inferences on exercise tolerance and prescription. *Am. J. Physiol. (Regul. Int. Comp. Physiol.)* 299: R1298-1305, 2010 a.

Salvadego D, R Domenis, S Lazzer et al. Does extreme muscle hypertrophy determine an impairment of skeletal muscle oxidative metabolism? 2011 Annual Meeting, American College of Sports Medicine. Denver, Colorado (USA). May 31-June 4, 2010 b.

Salvadego D, S Lazzer, M Marzorati et al. Impairment of skeletal muscle oxidative metabolism during knee-extension exercise after bed rest. Med Sci Sports Exerc 42: 5 (Supplement): S362, 2010 c.

Sastre, J., Asensi, M., Gasco, E., Pallardo, F. V., Ferrero, J. A., Furukawa, T., & Vina, J. (1992). Exhaustive physical exercise causes oxidation of glutathione status in blood: prevention by antioxidant administration. Am.J.Physiol 263, R992-R995.

Scheuring R, J Conkin, ML Gernhard, Risk assessment of physiological effects of atmospheric composition and pressure in Constellation vehicles. Acta Austronautica 63: 727-739.

Semenza G.L. (2000). HIF-1 and human disease: one highly involved factor. *Genes Dev 14:1983-1991*

Sen, C. K. (1999). Glutathione homeostasis in response to exercise training and nutritional supplements. Mol.Cell Biochem. 196, 31-42.

Sen, C. K. & Packer, L. (2000). Thiol homeostasis and supplements in physical exercise. Am.J.Clin.Nutr. 72, 653S-669S.

Siems, W., Capuozzo, E., Lucano, A., Salerno, C., & Crifo, C. (2003). High sensitivity of plasma membrane ion transport ATPases from human neutrophils towards 4-hydroxy-2,3-trans-nonenal. Life Sci. 73, 2583-2590.

Spiegelman B.M. and Flier J.S. (2001). Obesity and the regulation of energy balance. *Cell 104:531-543*

Srivastava, G., Bhatnagar, R., Viswanathan, R., & Venkitasubramanian, T. A. (1980). Microsomal & mitochondrial cytochromes in acutely hypoxic rat lung & liver. Indian J.Biochem.Biophys. 17, 130-134.

Stadtman, E. R. (1990). Metal ion-catalyzed oxidation of proteins: biochemical mechanism and biological consequences. Free Radic.Biol.Med. 9, 315-325.

Stadtman, E. R. (2001). Protein oxidation in aging and age-related diseases. Ann.N.Y.Acad.Sci. 928, 22-38.

Starke-Reed, P. E. & Oliver, C. N. (1989). Protein oxidation and proteolysis during aging and oxidative stress. Arch.Biochem.Biophys. 275, 559-567.

Stevens PM PB Miller, TD Lynch, CA Gilbert, RL Johnson, LE Lamb. Effects of lower body negative pressure on physiologic changes due to four weeks of hypoxic bed rest. Aerospace Med 466-474, 1966.

Stevenson, E. J., Giresi, P. G., Koncarevic, A., & Kandarian, S. C. (2003). Global analysis of gene expression patterns during disuse atrophy in rat skeletal muscle. J.Physiol 551, 33-48.

Subudhi AW, BR Miramon, ME Granger, RC Roach. Frontal and motor cortex oxygenation during maximal exercise in normoxia and hypoxia. *J Appl Physiol* 106: 1153-1158, 2009.

Sulzman F.M., J.S. Ferraro, C.A. Fuller, M.C. Moore-Ede, V. Klimovitsky, V. Magedov & A.M. Alpatov. 1992. Thermoregulatory responses of Rhesus monkeys during spaceflight. *Physiol Behav* 51: 585-591.

Summers RL, DS Martin, JV Meck, TG Coleman. Mechanisms of space flight-induced changes in left ventricular mass. Am J Cardiol 95: 1128-1130, 2005.

Taft RJ, Pang KC, Mercer TR, Dinger M, Mattick JS. Non-coding RNAs: regulators of disease. J Pathol. 2010 Jan;220(2):126-39. PubMed PMID: 19882673.

Taylor WE, Bhasin S, Lalani R, Datta A, Gonzalez-Cadavid NF. Alteration of gene expression profiles in skeletal muscle of rats exposed to microgravity during a spaceflight. J Gravit Physiol. 2002 Dec;9(2):61-70. PubMed PMID: 14638460.

Tidball, J. G. & Spencer, M. J. (2002). Expression of a calpastatin transgene slows muscle wasting and obviates changes in myosin isoform expression during murine muscle disuse. J.Physiol 545, 819-828.

Tonkonogi M, K Sahlin. Physical exercise and mitochondrial function in human skeletal muscle. *Exerc Sport Sci Rev* 30: 129-137, 2002.

Turrens, J. F., Freeman, B. A., & Crapo, J. D. (1982a). Hyperoxia increases H2O2 release by lung mitochondria and microsomes. Arch.Biochem.Biophys. 217, 411-421.

Turrens, J. F., Freeman, B. A., Levitt, J. G., & Crapo, J. D. (1982b). The effect of hyperoxia on superoxide production by lung submitochondrial particles. Arch.Biochem.Biophys. 217, 401-410.

Van Lieshout, JJ, Toska, K, Van Lieshout, EJ, Eriksen, M, Walloe, L, and Wesseling, KH. (2003). Beat-to-beat noninvasive stroke volume from arterial pressure and Doppler ultrasound. European Journal of Applied Physiology, 90: 131-137.

Vogiatzis I, D Athanasopoulos, H Habazettl et al. Intercostal muscle blood flow limitation in athletes during maximal exercise. J Physiol 587, 3665-3677. 2009.

Wabitsch M., Brenner R.E., Melzner I., Braun M., Möller P., Heinze E., Debatin K.M. and Hauner H. (2001). Characterization of a human preadipocyte cell strain with high capacity for adipose differentiation. *Int J Obes Relat Metab Disord. 25(1):8-15*

Ward M.P., Milledge J.S., West J.B. (2000). High Altitude Medicine and Physiology. Arnold: London.

Wayer C., Foley J.E., Bogardus C., Tataranni P.A. and Pratlay R.E. (2000). Enlarged subcutaneous abdominal adipocyte size, but not obesity itself, predicts Type II diabetes independent of insulin resistance. *Diabetol 43:1498-1506*

Wehrlin, JP, Zuest, P, Hallen, J, and Marti, B. (2006). Live high-train low for 24 days increases hemoglobin mass and red cell volume in elite endurance athletes. Journal of Applied Physiology, 100 (6): 1938-1945.

Wesseling, KH, Jansen, JR, Settels, JJ, and Schreuder, JJ. (1993). Computation of aortic flow from pressure in humans using a nonlinear, three element model. Journal of Applied Physiology, 74: 2566-2573.

Williams B.A. & R.D. Reese. 1972. Effect of bedrest on thermoregulation. Aerospace Medical Association Preprints: 140-141.

Williams AH, Valdez G, Moresi V, Qi X, McAnally J, Elliott JL, Bassel-Duby R, Sanes JR, Olson EN. MicroRNA-206 delays ALS progression and promotes regeneration of neuromuscular synapses in mice. Science. 2009 Dec 11;326(5959):1549-54.

Wood I.S., Wang B., Lorente-Cebrian S. and Trayhurn P. (2007). Hypoxia increases expression of selective facilitative glucose transporters (GLUT) and 2-deoxy-D-glucose uptake in human adipocytes. *Biochem Biophys Res Commun 361: 468–473*

Ye J., Gao Z., Yin J. and He Q. (2007). Hypoxia is a potential risk factor for chronic inflammation and adiponectin reduction in adipose tissue of ob/ob and dietary obese mice. *Am J Physiol Endocrinol Metab 293: E1118–E1128*

Yin J., Gao Z., He Q., Zhou D., Guo Z. and Ye J. (2009). Role of hypoxia in obesity-induced disorders of glucose and lipid metabolism in adipose tissue. *Am J Physiol Endocrinol metab 296:E333-E342*

Yoshikawa, T., Furukawa, Y., Wakamatsu, Y., Tanaka, H., Takemura, S., & Kondo, M. (1982). The increase of thiobarbituric acid reacting substances in rats with experimental chronic hypoxia. Experientia 38, 312-313.

V, S.M. Lee, Y. Kobzev, R.R. Gonzalez & J.E. Greenleaf. 1998. Body temperature and thermoregulation during submaximal exercise after 115-day spaceflight. Aviat Space Environ Med. 69:137-41.
